# Burden of falls associated with low bone density globally, in China and ASEAN countries: 1990–2021 trends and projections to 2035

**DOI:** 10.3389/fmed.2025.1638057

**Published:** 2025-10-20

**Authors:** Rui Huang, Yilin Teng, Roumei Wang, Ye Tong, Ming Lei, Baicheng Wan, Shaohui Zong, Gaofeng Zeng

**Affiliations:** ^1^Department of Spine Osteopathia, The First Affiliated Hospital of Guangxi Medical University, Guangxi Medical University, Nanning, Guangxi, China; ^2^Department of Medical Ultrasound, The First Affiliated Hospital of Guangxi Medical University, Nanning, Guangxi, China; ^3^Wuming Hospital of Guangxi Medical University, Nanning, Guangxi, China; ^4^College of Public Hygiene of Guangxi Medical University, Nanning, Guangxi, China

**Keywords:** falls, low bone mineral density, Association of Southeast Asian Nations-ASEAN, trends, DALYs

## Abstract

**Background:**

Falls have become a critical challenge for the global elderly population, posing a serious public health issue. Therefore, this study aims to evaluate the current burden of falls associated with low bone density (LBMD) and analyze its trends from 1990 to 2021 globally, in China, and in ASEAN (Association of Southeast Asian Nations) countries, while also projecting future trends up to 2035.

**Methods:**

We incorporated the Socio-demographic Index (SDI) to assess the influence of developmental levels, employed the estimated annual percent change (EAPC) and joinpoint regression to capture and quantify temporal trends, and applied frontier analysis to evaluate efficiency gaps and the potential for improvement across countries. In addition, we used the Bayesian Age-Period-Cohort (BAPC) model to forecast future disease burden trends.

**Results:**

From 1990 to 2021, global deaths from falls due to LBMD increased from 122,143 to 324,557, and disability-adjusted life years (DALYs) increased from 5.02 to 11.34 million. Despite this trend, ASMR remained stable (EAPC = 0.18%) and ASDR declined slightly (EAPC = −0.15%). China and ASEAN countries experienced a rising burden, accounting for 23.29% of deaths and 24.41% of DALYs in 2021. ASMR and ASDR increased with age, peaking in those aged ≥80 years, with older females bearing a higher burden. Frontier analysis identified Cambodia, Vietnam and Myanmar as having the greatest potential for improvement. By 2035, global ASMR and ASDR are projected to decline, though ASDR may show increase in countries such as Cambodia and Thailand.

**Conclusion:**

This study integrated joinpoint, frontier, and BAPC analyses, providing new insights into the association between burden of falls and low bone density globally and in China and ASEAN countries. The results indicated that the burden of falls associated with low bone density remains substantial worldwide, in China, and in ASEAN countries, particularly in low-income nations and among individuals aged 80 years and older. Therefore, it is necessary to prioritize fall prevention and bone health strategies, especially in resource-limited settings, while optimizing healthcare resource allocation and strengthening policy interventions to effectively alleviate this global health challenge.

## 1 Introduction

Accidents from falls represent a significant global public health burden. According to the World Health Organization (WHO), after traffic accidents, falls rank as the second leading cause of unintentional injury and mortality. An estimated 684,000 annual deaths are attributed to falls, with over 80% occurring in low- and middle-income countries, particularly in the Western Pacific and Southeast Asia regions (1). Globally, approximately one-third of individuals aged 65 and older experience at least one fall annually (2). Falls lead to injuries, loss of independence, and trigger a fear of falling among older adults (3). In addition, falls contribute substantially to morbidity, with 37.3 million severe incidents requiring medical attention each year, significantly impacting global disability-adjusted life years (DALYs) (4). As global demographics and policies evolve, the injury and economic burdens associated with falls are expected to exhibit regional disparities.

Low bone mineral density (LBMD), a condition characterized by increased bone resorption, decreased bone formation, or a combination of both, is a key factor contributing to an elevated risk of falls. The progression of LBMD can be classified into two stages: osteopenia and osteoporosis (5). Globally, especially in regions experiencing rapid population aging and the uneven distribution of healthcare resources, LBMD-related issues are more pronounced. Southeast Asia is one of the regions currently confronted with challenges such as demographic shifts, an increasing burden of non-communicable diseases, and healthcare system transitions (6, 7). Against this background, regional studies have further highlighted the prevalence of LBMD and its implications. It has been reported that the highest burden attributed to LBMD was observed in the Southeast Asia Region (8). Specifically, studies have shown that in the Philippines, 97.2% of adults and 95.5% of elderly individuals suffer from calcium deficiency, a key contributor to poor bone health (9). In Vietnam, nearly half of women and over one-third of men aged 50 and above meet the osteoporosis treatment criteria (10). In Singapore, over one-third of women aged 50+ was reported to have osteoporosis and the highest hip fracture incidence in Asia (11). Even in countries with abundant sunlight, such as Cambodia, the proportion of individuals with vitamin D deficiency remains high (12). These findings underscore the urgent need for further research in these regions.

The Association of Southeast Asian Nations (ASEAN), comprising Brunei, Cambodia, Indonesia, Laos, Malaysia, Myanmar, the Philippines, Singapore, Thailand, and Vietnam, represents Asia’s third-largest regional economy and the fifth-largest globally (13). Despite advancements in the China-ASEAN cooperation in disease prevention, the region’s vast geography, large populations, and socio-economic diversity present complex challenges in optimizing healthcare systems and addressing evolving health service needs.

Recent data on the burden of falls associated with LBMD in China and ASEAN countries regions remain scarce. To address this issue, this study analyzed long-term trends in LBMD-related fall burdens from 1990 to 2021, including deaths and DALYs rates, explored their association with the Socio-demographic Index (SDI), and made an attempt to apply frontier analysis in assessing potential for improvement. Using the Bayesian Age-Period-Cohort (BAPC) model, we projected potential public health challenges by 2035, offering data-driven insights to inform effective strategies for the prevention and management of LBMD-related falls in China and ASEAN countries.

## 2 Materials and methods

### 2.1 Data source

This study used data from the Global Burden of Disease Study 2021 (GBD 2021), focusing on fall-related data (1990–2021) associated with LBMD risk factors, particularly deaths and DALYs. Within the GBD framework, “risk factors” denote elements influencing the LBMD burden, quantified via attributable risk assessment. Falls, defined as injuries from unintentional downward movements resulting in lower-level impacts (14), were examined alongside LBMD, measured as femoral neck bone mineral density (g/cm^2^) via dual-X-ray absorptiometry (DXA) (8). LBMD encompasses osteopenia (*T*-score: −1 to −2.5) and osteoporosis (*T*-score ≤ −2.5) (5). We reported LBMD-attributable fall-related deaths and DALYs (1990–2021) globally, in China, and ASEAN nations (Brunei, Cambodia, Indonesia, Laos, Malaysia, Myanmar, Philippines, Singapore, Thailand, Vietnam).

### 2.2 Statistical analysis

This study assessed deaths and DALYs using age-standardized rates (ASRs per 100,000 population) with 95% uncertainty intervals (UIs) based on annual population data from 1990 to 2021 provided by the GBD study. ASRs, including age-standardized mortality rate (ASMR) and age-standardized DALY rates (ASDR), were referenced from previous studies (15, 16). The ASR values were calculated as follows:


$$\mathrm{ASR}=\frac{\sum_{i=1}^{A}\;a_{i}w_{i}}{\sum_{i=1}^{A}\;w_{i}}\times 100,000$$

where $a_{i}$ denotes the age specific rate of age group i in the population, $w_{i}$ represents the number of persons in the age group i . ASRs offer precise epidemiological assessments by adjusting for age structure variations, effectively reflecting disease trends. The ASR is determined by calculating the weighted average of the age-specific rates, where the weights are derived from the distribution of the standard population. This is done by multiplying the age-specific rates ( $a_{i}$ ) with the number of individuals ( $w_{i}$ ) in each corresponding age group, summing these products, and then dividing by the total weight of the standard population (17).

### 2.3 Joinpoint regression model

We performed joinpoint regression to assess trends in ASMR and ASDR related to low bone mineral density falls from 1990 to 2021. Piecewise regression was used to segment the data at points of significant change. The annual percent change (APC) was calculated as the geometric mean of the annual changes and was considered statistically significant if its 95% confidence interval excluded zero, with *p* < 0.05.

### 2.4 Frontier analysis and BAPC prediction

We used frontier analysis to identify the driving factors behind the burden of falls related to LBMD. Using the SDI, we developed frontier models based on ASDR and ASMR, and compared the performing nations and areas with other nations and regions. This strategy recognizes leading countries and regions, then establishes criteria and goals for others. We calculated the “effective differences” for each country and area, which represent the difference between the current and potential burdens, adjusted for the SDI (18, 19). Given the limited sample size, this frontier analysis was restricted to 11 decision-making units, and this context should be borne in mind when evaluating the results. The specific formula can be found in Appendix 1. Local weighted regression (LOESS) and local polynomial regression were applied to generate smoothed curves. We performed 1,000 bootstrap resamples to assess the improvement potential of each country. Additionally, we used the BAPC model to predict the LBMD fall burden through 2035. A key advantage of the BAPC model is the use of integrated nested Laplace approximation (INLA), which efficiently addresses challenges like mixing and convergence issues common in Markov chain Monte Carlo methods while ensuring computational efficiency. The model’s flexibility in handling time series data makes it particularly suitable for long-term disease burden predictions.

All statistical analyses were performed using R software (version 4.2.3), with a two-sided *p* < 0.05 considered statistically significant.

## 3 Results

### 3.1 Disease burden attributable to LBMD

We demonstrate the trends in outcomes of deaths and DALYs associated with falls related to LBMD globally, as well as in China and ASEAN countries. The trends in outcomes of deaths and DALYs associated with falls related to LBMD globally, as well as in China and ASEAN countries. From 1990 to 2021, the total global deaths linked to falls associated with LBMD increased from 122143.08 to 324557.11. During the same period, the ASMR remained largely stable, changing from 4.09 to 4.08, with an EAPC of 0.18%. Similarly, global DALYs rose from 5021293.29 in 1990 to 11339001.73 in 2021, while the ASDR decreased from 142.22 in 1990 to 135.96 in 2021, with an EAPC of −0.15%. In 1990, China and ASEAN countries collectively accounted for 19.14% of global deaths and 18.93% of global DALYs related to falls associated with LBMD. By 2021, these proportions increased to 23.29 and 24.41%, respectively.

**Table 1:** EAPCs and changes in burden of falls associated with LBMD in China and ASEAN countries from 1990 to 2021.

Measure	1990		2021		EAPC (95% CI)
	Number (95% UI)	ASR (95% UI)	Number (95% UI)	ASR (95% UI)	
Deaths					
Global	122143.08 (103792.56, 136340.73)	4.09 (3.42, 4.58)	324557.11 (265049.14, 371850.54)	4.08 (3.33, 4.68)	0.18 (0.10, 0.26)
China	15172.65 (12298.63, 19669.93)	3.59 (2.94, 4.56)	56030.27 (33901.22, 74700.46)	3.49 (2.09, 4.69)	0.26 (−0.13, 0.66)
Brunei	1.89 (1.47, 2.40)	2.66 (2.05, 3.30)	4.38 (3.50, 5.23)	2.19 (1.67, 2.68)	0.15 (−0.15, 0.46)
Cambodia	235.40 (187.61, 298.94)	8.35 (6.39, 10.32)	737.40 (551.14, 912.42)	9.60 (6.88, 11.98)	0.47 (0.37, 0.58)
Indonesia	2918.19 (1871.27, 3759.09)	4.61 (2.84, 6.14)	6409.16 (4671.65, 7723.05)	4.65 (3.23, 5.76)	−0.19 (−0.38, −0.00)
Laos	50.36 (35.76, 68.73)	3.68 (2.74, 4.91)	115.74 (89.48, 144.46)	3.73 (2.87, 4.60)	0.05 (0.01, 0.09)
Malaysia	201.32 (150.48, 241.98)	2.59 (1.92, 3.15)	523.61 (364.79, 628.70)	2.39 (1.65, 2.89)	−0.18 (−0.39, 0.04)
Myanmar	895.01 (677.34, 1149.44)	5.99 (4.44, 7.58)	1867.91 (1405.41, 2411.22)	5.27 (3.79, 6.76)	−0.63 (−0.73, −0.54)
Philippines	437.38 (320.33, 595.06)	2.45 (1.82, 3.26)	1402.65 (1055.70, 1671.65)	2.38 (1.78, 2.83)	0.38 (0.24, 0.52)
Singapore	23.83 (20.88, 25.74)	1.40 (1.21, 1.52)	84.86 (69.47, 94.71)	1.04 (0.85, 1.16)	−1.36 (−1.65, −1.07)
Thailand	842.51 (666.21, 1036.14)	3.37 (2.60, 4.23)	2191.58 (1527.31, 3400.38)	2.00 (1.40, 3.11)	−2.36 (−2.69, −2.03)
Vietnam	2603.56 (1380.36, 3767.54)	8.18 (4.17, 11.98)	6174.94 (2985.97, 8707.27)	8.37 (3.82, 12.01)	0.10 (0.05, 0.14)
DALYs					
Global	5021293.29 (4099632.85, 6063370.32)	142.22 (116.43, 170.89)	11339900.73 (9233705.79, 13894399.85)	135.96 (110.74, 166.56)	−0.15 (−0.17, −0.13)
China	702308.29 (565223.05, 867176.46)	106.26 (86.71, 129.50)	2131657.59 (1602677.91, 2710848.21)	111.62 (83.97, 140.87)	0.03 (−0.16, 0.21)
Brunei	113.50 (88.80, 139.65)	120.00 (94.43, 146.82)	343.05 (266.09, 434.39)	112.48 (88.32, 140.81)	−0.00 (−0.07, 0.06)
Cambodia	6524.60 (5322.52, 8177.27)	180.21 (148.26, 219.95)	21441.07 (17257.50, 26157.64)	217.75 (172.70, 263.26)	0.65 (0.56, 0.74)
Indonesia	101964.09 (78570.58, 123402.64)	128.24 (96.23, 158.02)	207541.04 (168522.85, 244110.32)	113.54 (90.86, 134.08)	−0.68 (−0.83, −0.53)
Laos	1572.73 (1184.16, 2052.82)	89.51 (68.73, 114.63)	3629.28 (2920.54, 4358.37)	93.36 (76.17, 111.66)	0.16 (0.14, 0.18)
Malaysia	6661.29 (5418.53, 7806.58)	78.87 (64.11, 92.63)	18929.43 (15287.11, 22923.97)	74.91 (60.29, 90.70)	−0.16 (−0.23, −0.08)
Myanmar	26500.78 (20754.97, 32566.88)	138.89 (110.38, 169.12)	57554.93 (46057.72, 72311.43)	136.83 (109.60, 167.77)	−0.20 (−0.26, −0.14)
Philippines	18536.17 (14676.80, 23245.13)	74.02 (59.69, 91.85)	51748.13 (42123.39, 61909.87)	72.11 (58.92, 85.71)	0.06 (−0.02, 0.15)
Singapore	1918.61 (1458.19, 2459.30)	90.53 (69.49, 114.58)	6919.08 (5088.01, 9056.94)	81.06 (59.75, 105.87)	−0.55 (−0.79, −0.32)
Thailand	27285.17 (22222.21, 32594.47)	89.94 (72.99, 106.34)	85773.48 (66633.73, 109620.15)	78.48 (61.04, 100.32)	−0.74 (−0.91, −0.56)
Vietnam	58549.09 (40217.22, 76781.39)	167.77 (113.37, 222.30)	153546.99 (106745.04, 193585.42)	184.86 (125.49, 235.70)	0.36 (0.31, 0.41)

ASR, age-standardized rate; LBMD, low bone mineral density; EAPC, estimated annual percentage change; ASEAN, Association of South East Asian Nations.

**Figure 1:**
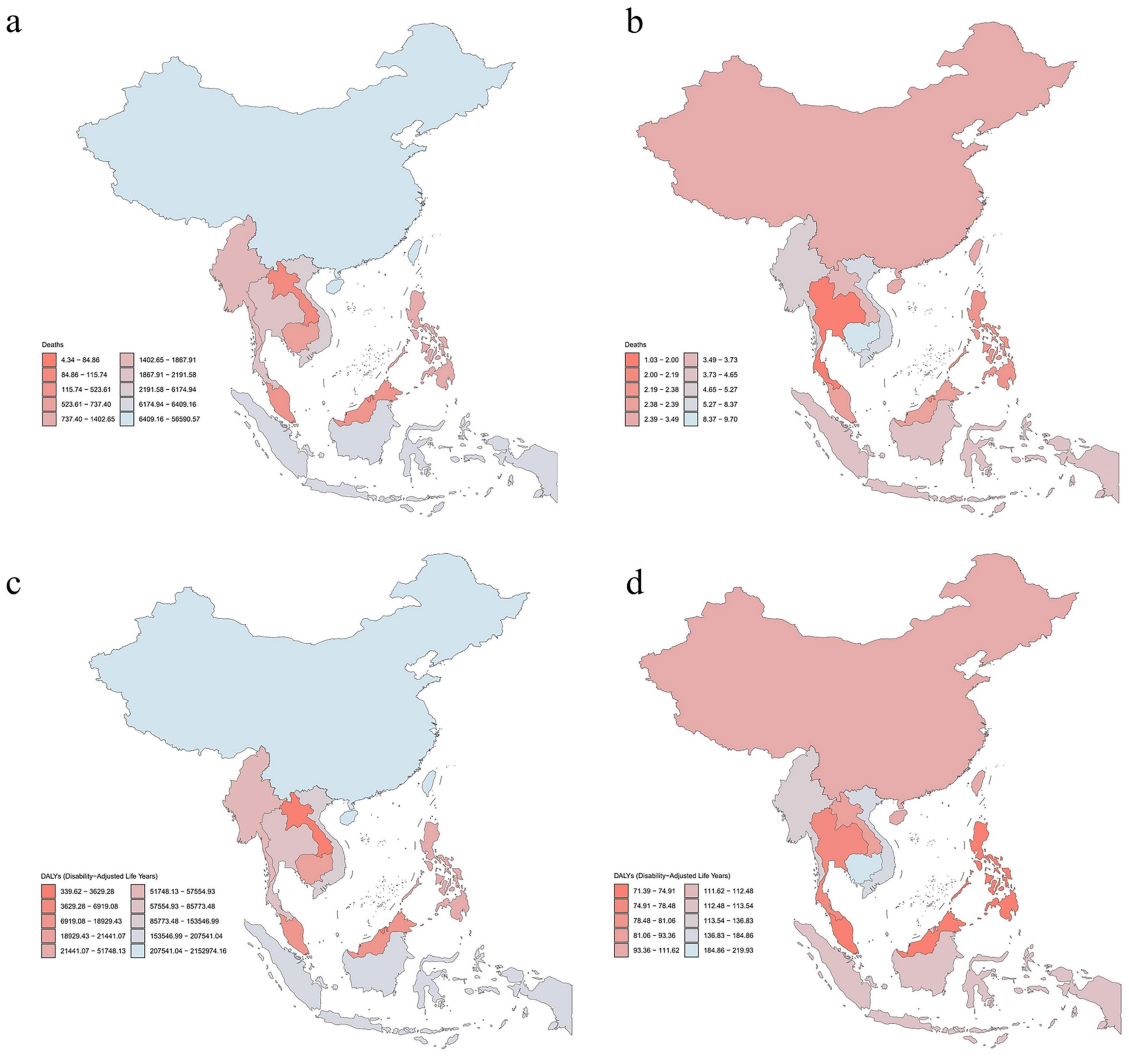
Numbers and age-standardized rates of LBMD-related deaths and DALYs in China and ASEAN in 2021. **(a)** Number of deaths. **(b)** ASMR. **(c)** Number of DALYs. **(d)** ASDR. LBMD, low bone mineral density; DALYs, disability-adjusted life years; ASEAN, Association of South East Asian Nations; ASMR, age-standardized mortality rate; ASDR, age-standardized DALY rate.

In 2021, China reported the highest number of deaths linked to falls associated with LBMD with 56030.27. Among ASEAN countries, Indonesia recorded the highest number of deaths at 6409.16, followed by Vietnam with 6174.94 and Thailand with 2191.58. Regarding DALYs in 2021, China reported 2131657.59 person-years, while Indonesia led ASEAN countries with 207541.04, followed by Vietnam (153546.99) and Thailand (85773.48). These results are presented in Table 1 and Figure 1.

### 3.2 Trends in ASMR and ASDR from falls with LBMD between 1990–2021

**Figure 2:**
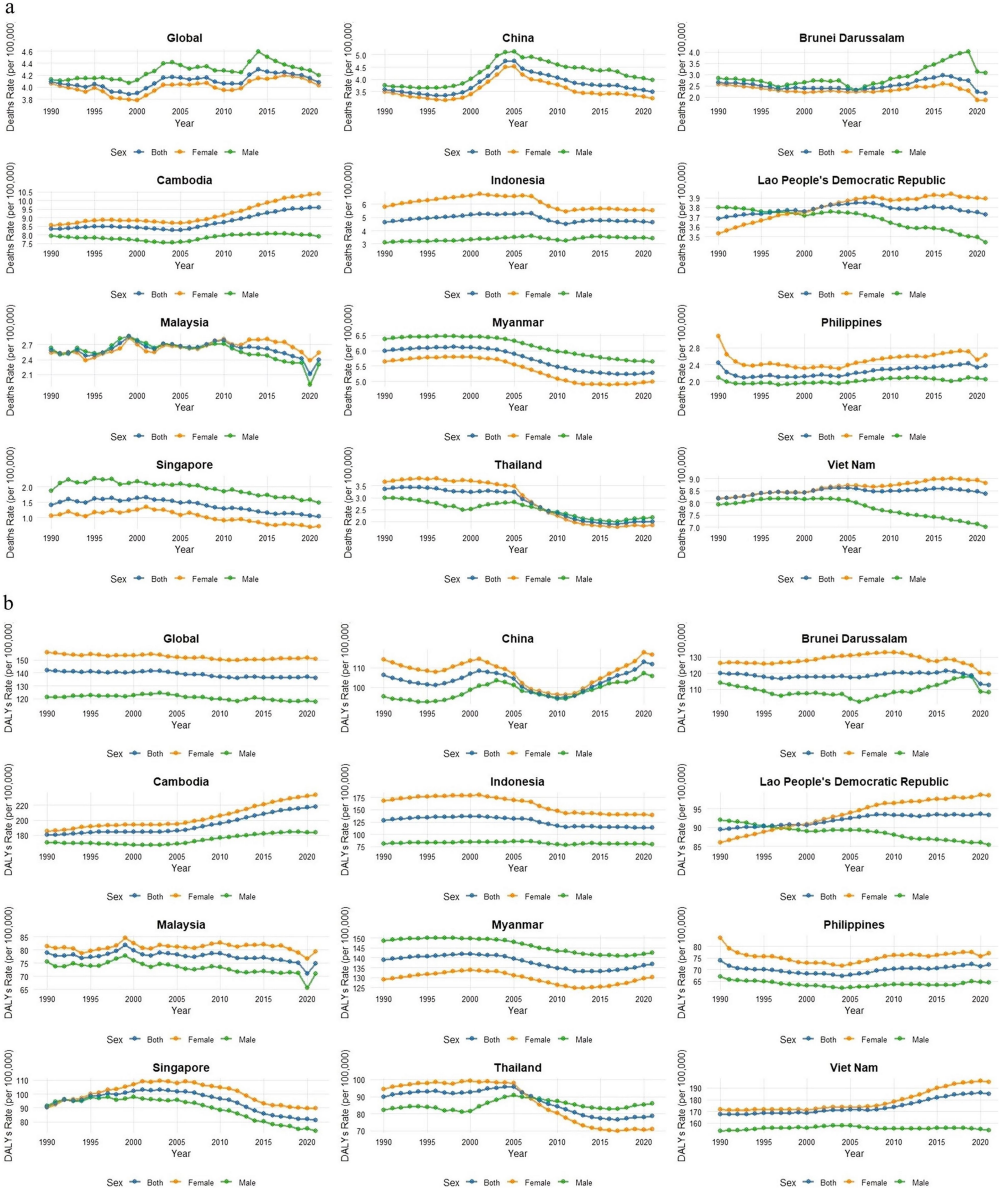
The ASMR and ASDR attributable to falls caused by LBMD globally, in China, and in ASEAN countries from 1990 to 2021. **(a)** ASMR trends by country (1990–2021). **(b)** ASDR trends by country (1990–2021). LBMD, low bone mineral density; DALYs, disability-adjusted life years; ASEAN, Association of South East Asian Nations; ASMR, age-standardized mortality rate; ASDR, age-standardized DALY rate.

Globally, from 1990 to 2021, ASMR attributable to falls exhibited a fluctuating trend, with males consistently in higher proportion than females. In China, ASMR increased overall between 1990 and 2005, peaking around 2005, and subsequently declined. In Brunei Darussalam, the overall and female ASMR showed a declining trend, whereas male rates increased, reaching a peak in 2019. Contrary to the global trend, in Cambodia, Indonesia, the Philippines, and Vietnam, ASMR in females were consistently higher than males. In the Lao People’s Democratic Republic, female ASMR rates transitioned from being lower than male rates to consistently higher after 1999, with Malaysia showing a similar transition in 2008. In Thailand, however, female ASMR rates became persistently lower than male rates after 2008. Myanmar and Singapore followed the global trend, with the overall ASMR declining (Figure 2a).

Globally, ASDR remained relatively stable and declined slightly over the study period, with female ASDR consistently exceeding that of male ASDR. In China, the ASDR rate followed a W-shaped trend, with the gender gap narrowing year by year after 2000 but showing a steady increase after 2011. Malaysia and the Philippines exhibited ASDR rates consistently below the global average, whereas Cambodia’s ASDR was higher than the global average. The trends in Brunei Darussalam and Indonesia closely aligned with the global average. In the Lao People’s Democratic Republic and Singapore, ASDR was generally lower than the global average, but the gender gap widened progressively after 2000. In Thailand, the female ASDR transitioned from being higher than male ASDR to lower after 2009, with the gender gap expanding over time. In Vietnam, ASDR cases were consistently above the global average between 1990 and 2021, exhibiting a steady upward trend (Figure 2b).

### 3.3 Joinpoint regression analysis results of ASMR and ASDR from falls attributed to LBMD between 1990–2021

Globally, ASMR declined from 1990 to 2000 (APC = −0.51%), increased during 2000–2003 (APC = 2.01%), rose slightly from 2003 to 2019 (APC = 0.18%), and decreased after 2019 (APC = −1.43%). In China, ASMR markedly increased during 1998–2004 (APC = 6.72%) but declined in other periods. Thailand showed continuous declines from 1990 to 2016, followed by an increase after 2016 (APC = 1.26%). In Singapore, ASMR consistently decreased after 2001 (APC = −2.41, −1.76%). Malaysia exhibited a significant decline in ASMR during 2015–2021 (APC = −2.40%). ASMR in Brunei Darussalam increased between 2007 and 2017 (APC = 2.53%). Cambodia showed a decline in ASMR only during 1996–2005 (APC = −0.30%), while other periods showed increase. ASMR in Indonesia declined during 2006–2010 (APC = −4.53%) and 2014–2021 (APC = −0.40%). ASMR in Lao PDR increased before 2004 (APC = 0.22, 0.46%) but declined thereafter (APC = −0.13, −0.36%). ASMR in the Philippines increased after 2004 (APC = 1.41, 0.36%). It declined in Vietnam during 2005–2008 (APC = −0.54%) and 2017–2021 (APC = −0.62%), with increases in other periods (Supplementary Table S3). Globally, DALY rates decreased during 1990–1999 (APC = −0.13%) and 2002–2011 (APC = −0.40%), with no significant changes in other periods. Malaysia, Singapore, and Indonesia experienced continuous declines in DALY rates after 1998, 2001, and 2000, respectively. DALY in Cambodia, Vietnam, and Lao PDR showed overall increasing trends, it exhibited mixed trends of rises and falls in China, Myanmar, Thailand, and Brunei Darussalam. DALY in the Philippines declined before 2004 (APC = −2.81, −0.39%) but increased continuously thereafter (APC = 0.86, 0.25%) (Supplementary Table S4).

### 3.4 Age- and sex-stratified analysis of LBMD-related falls burden from 1990 to 2021

**Figure 3:**
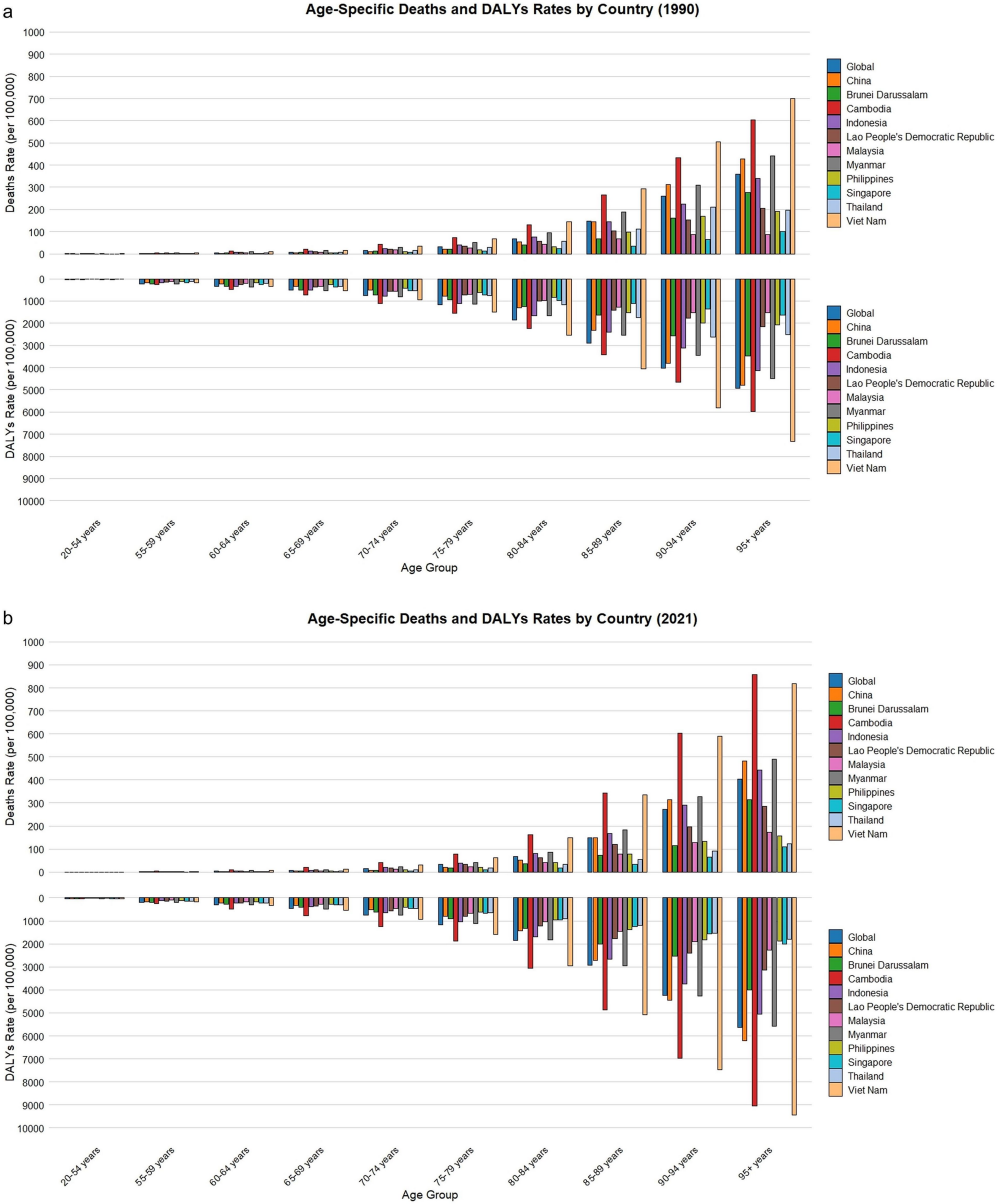
Changes in DALYs and death rates due to falls caused by LBMD for different age groups globally, in China, and in ASEAN countries in 1990 and 2021. **(a)** Age-specific deaths and DALYs rates by country (1990). **(b)** Age-specific deaths and DALYs rates by country (2021). The top of each bar represents the specific burden for each country in the respective age group. LBMD, low bone mineral density; ASEAN, Association of South East Asian Nations.

Global data showed that ASMR and ASDR rose with age in both 1990 and 2021, remaining low under 65 but sharply increasing at ≥80 years. Nationally, China and most ASEAN countries exhibited age-related rises, with higher rates in those ≥85 years in 2021 than in 1990. Within ASEAN countries, Vietnam, Myanmar, and Cambodia showed a particularly high burden among the oldest groups, while Singapore and Thailand remained relatively stable with minimal changes (Figures 3a,b). A further sex-stratified analysis of the disease burden across age groups revealed that, at the global level, the number of deaths was higher in males than in females among individuals younger than 60 years, whereas in those aged 60 years and above, females had more deaths than males. In China, this sex-related shift occurred at 75 years, with males having more deaths before this age and females surpassing males thereafter. A similar trend was observed in most ASEAN countries, where the burden gradually shifted from male predominance in younger age groups to female predominance in older age groups. However, Singapore exhibited a distinct pattern, with males consistently having more deaths than females until the age of 95 years, beyond which this trend reversed. Globally, the number of DALYs was higher in males than in females among individuals younger than 50 years. However, in those aged 50 years and above, females bore a greater burden, with an increasing disparity between sexes. This trend was also evident in China and ASEAN countries, although the specific age thresholds varied. Notably, even in Singapore—where males consistently had more deaths than females up to the age of 95 years—the number of DALYs was greater in females across all age groups (Supplementary Figures S3, S4).

### 3.5 Relationship between SDI and ASR of burden of falls associated with LBMD in China and ASEAN countries and frontier analysis

**Figure 4:**
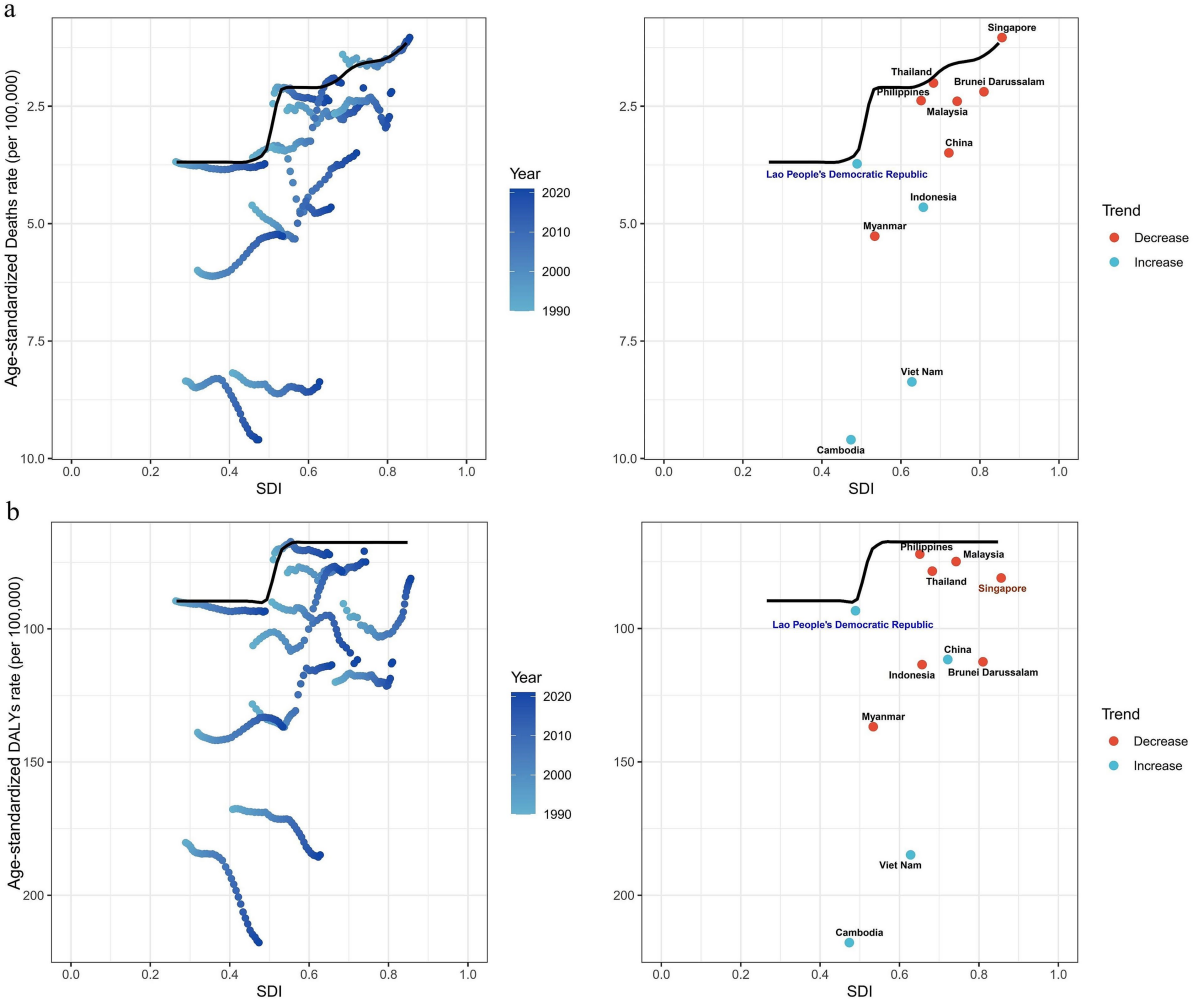
Frontier analysis of SDI and burden of falls associated with LBMD in 2021. **(a)** Frontier analysis based on ASMR. **(b)** Frontier analysis based on ASDR. Countries are represented by solid points, and those exhibiting relatively large efficiency differences are marked in black text. The frontier boundary is delineated by a solid black line. Countries with a low SDI (<0.5) and a decline in efficiency are highlighted in blue text, while those with a high SDI (>0.85) and relatively higher efficiency differences compared to their level of development are marked in red text. Red dots represent a reduction in the burden of falls associated with LBMD from 1990 to 2021, while blue dots indicate an increase in the same period. LBMD, low bone mineral density; SDI: Socio-demographic Index.

This study further investigated the relationship between regional variations in SDI levels, ASMR and ASDR. The results showed that as SDI increased, the relationship between ASMR and SDI followed an overall downward trend. In the SDI range of 0.3 to 0.5, there was considerable variability in death rates between countries, with Vietnam and Cambodia exhibiting higher ASMR levels compared to the global average, China, and other ASEAN countries. As SDI continued to increase, particularly in countries with an SDI greater than 0.5, ASMR in high-SDI nations were generally lower and more concentrated. Singapore had an ASMR lower than the global average and remained consistently lower than other countries (Supplementary Figure S2a). Furthermore, we could observe an overall negative correlation between SDI and ASDR. However, the trends for Vietnam and Cambodia diverged from the overall pattern. Between 1990 and 2021, despite increases in SDI, the ASDR in these countries showed an upward trajectory and consistently remained higher than the global average, China, and other ASEAN nations. In addition, similar to ASMR, there was a large disparity in ASDR among countries within the SDI range of 0.3 to 0.5, whereas in countries with a higher SDI (>0.5), ASDR tended to be more concentrated. Notably, some high-SDI countries, such as Brunei Darussalam, despite having relatively high SDI values, still exhibited relatively high ASDR (Supplementary Figure S2b). Based on the above observations, we conducted a further frontier analysis to explore the potential for improvement in the burden of falls associated with LBMD, considering the level of national development (Figures 4a,b). The three regions with the largest potential for improvement in terms of actual efficiency differences were Cambodia, Vietnam and Myanmar. Laos, with a low SDI, was identified as a frontier country. Additionally, Singapore has already reached the optimal level in managing the ASMR burden of falls associated with LBMD. Despite the low SDI of Laos, it demonstrated remarkable effectiveness in controlling falls associated with LBMD.

### 3.6 Predicted global ASMR and ASDR from 1990 to 2035 based on the BAPC model

**Figure 5:**
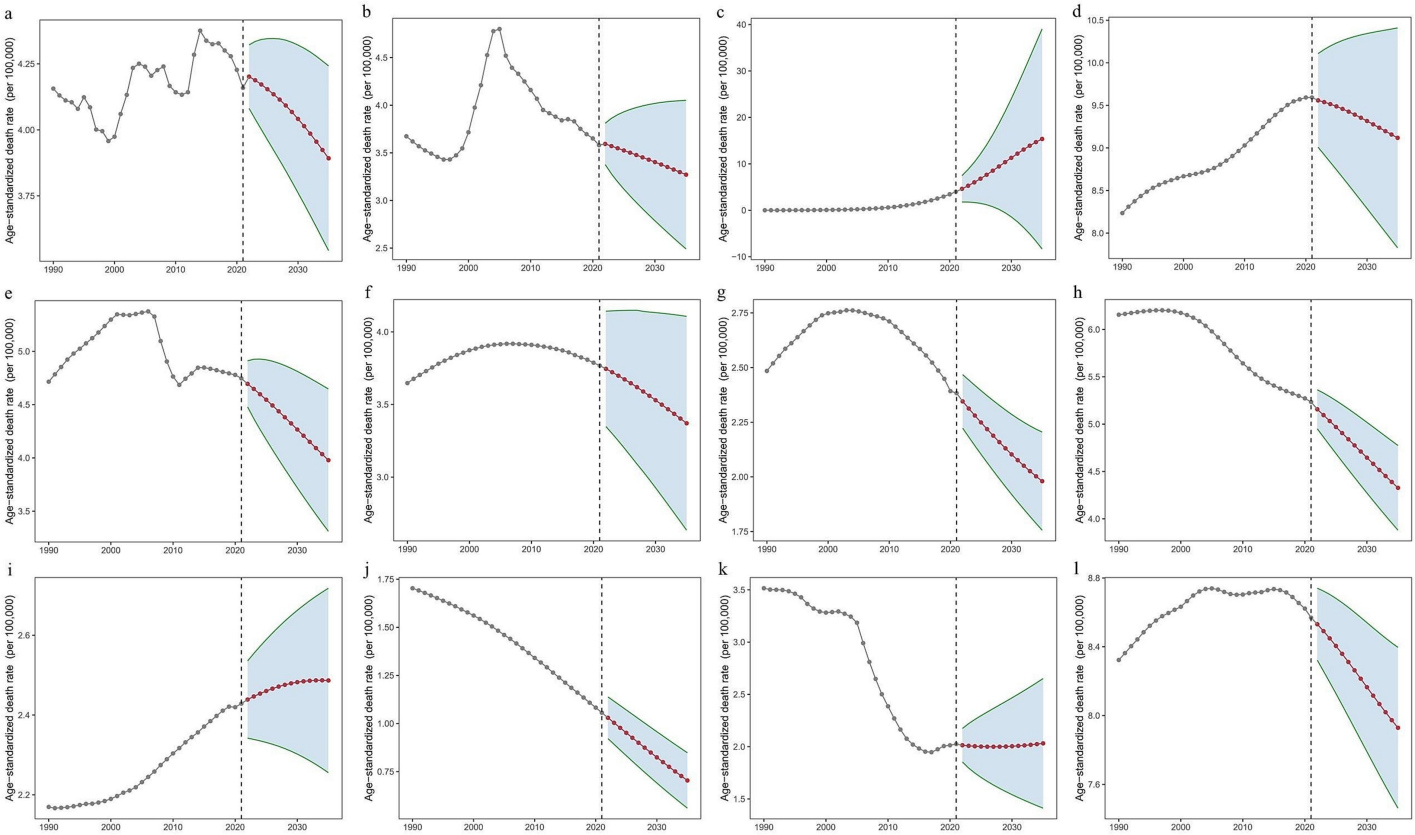
Predicted ASMR globally from 1990 to 2035, based on the BAPC model. **(a)** Worldwide. **(b)** China. **(c)** Brunei Darussalam. **(d)** Cambodia. **(e)** Indonesia. **(f)** Lao People’s Democratic Republic. **(g)** Malaysia. **(h)** Myanmar. **(i)** Philippines. **(j)** Singapore. **(k)** Thailand. **(l)** Vietnam. BAPC, Bayesian Age-Period-Cohort.

On the basis of the BAPC model, this study predicted the ASMR and ASDR from 2022 to 2035. The forecast analysis revealed a general downward trend in both ASMR and ASDR globally and across most ASEAN regions. Specifically, by 2035, global ASMR and ASDR are projected to decrease to 3.89 per 100,000 population (95% CI: 3.54–4.24) and 111.50 per 100,000 population (95% CI: −192.5–415.52), respectively (see Figure 5; Supplementary Figure S1 and Supplementary Tables S1, S2). This suggests that by 2035, the number of deaths globally due to falls related to LBMD is expected to increase to approximately 347,249 (95% CI: 316,026–378,473). In China and Thailand, ASMR is predicted to decrease slowly after 2021; however, beyond 2021, Brunei and the Philippines are expected to experience a slight increase in ASMR. The ASMR trend in other ASEAN countries aligns closely with the global pattern. Among ASEAN nations, Singapore is projected to reach the lowest ASMR by 2035, with a value of 0.70 per 100,000 population (95% CI: 0.56–0.85), showing the most stable and notable decline. Cambodia and Thailand, however, are forecasted to experience an increase in ASDR after 2021, with Cambodia’s rate projected to rise to 222.63 (95% CI: 203.65–241.60) and Thailand’s rate to 85.59 (95% CI: 72.00–99.18) by 2035. Detailed predictions of ASMR and ASDR from 2022 to 2035 are presented in Supplementary Tables S1, S2.

## 4 Discussion

Previous research in China has demonstrated that, between 1990 and 2019, mortality and DALY rates of falls attributable to low bone mineral density increased among adults aged 60 years and older, with the steepest rises in those aged 80 years and above (5). These findings provide critical evidence for understanding the health challenges faced by the elderly population in China. Building on this evidence, the present study further extended the analysis to examine trends in the burden of LBMD-related falls from 1990 to 2021 across the global, Chinese, and ASEAN contexts. By 2021, approximately 324,557 global deaths and 11,339,900 DALYs were attributed to these falls. The burden was primarily concentrated in those aged 55 and older, particularly individuals aged 80+, with significant increases in both ASMR and ASDR, underscoring the global public health impact. From 1990 to 2021, the global ASMR increased by 0.18 EAPC, while DALYs decreased by −0.15 EAPC. This pattern, consistent with joinpoint regression analysis, reflects the aging population’s rising fall-related mortality risk, despite reductions in the overall DALY burden due to effective interventions. While death rates have risen, improvements in public health and prevention have mitigated the overall health burden, particularly in developed countries. Our findings also highlight that ASMR is consistently higher in males, likely due to greater risk-taking behaviors and occupational hazards (20, 21).

Although the global trend shows positive changes, the trends in ASMR and ASDR in China and ASEAN countries do not fully align with the global patterns. This discrepancy reflects the complex interplay of factors such as socioeconomic development, healthcare levels, and population aging, and underscores the uneven progress across countries and regions in addressing the challenges associated with falls associated with LBMD. For instance, diverging from the global trend, the ASMR for women is higher than that for men in Cambodia, Indonesia, the Philippines, and Vietnam. This may be linked to gender inequalities in education, health, living conditions, and sanitation in these countries (22). We also observed that China’s ASMR peaked around 2005 and has since gradually declined. Since the 1990s, China has strengthened its efforts in osteoporosis prevention and treatment, incorporating these into the national “9th Five-Year,” “15th Five-Year,” and “11th Five-Year” supporting projects. In 2002, the former Ministry of Health conducted China’s first prospective epidemiological survey of osteoporosis prevalence in 13 cities. The following year, the Ministry of Health approved the introduction of international bone density measurement training to enhance the country’s diagnostic capacity for osteoporosis. In 2008, the China Health Promotion Foundation, in collaboration with the International Osteoporosis Foundation, released the “Osteoporosis Prevention and Treatment China White Paper” and signed a memorandum of cooperation, advancing international cooperation in osteoporosis prevention and treatment to a new level (23). However, the increase in DALYs reflects the growing burden of long-term disability caused by falls, exacerbated by population aging, particularly with the rising proportion of those aged 80 and above. On the other hand, between 1990 and 2021, ASMRs in middle- and high-income countries, like Thailand, and Singapore as a developed country, showed a downward trend, while in lower-middle-income countries such as Cambodia and developing nations like Vietnam, mortality rates steadily increased, with their DALY burdens also continuing to rise. In some lower-middle-income countries in the Asia-Pacific region, osteoporosis has yet to be included in national action plans, coupled with a lack of relevant epidemiological data, social cost information, and quality-of-life data. In fact, most patients in these countries have not undergone bone density screening or treatment. Furthermore, the slow progress in the development and implementation of osteoporosis-related health policies, coupled with insufficient healthcare resources in terms of availability, accessibility, affordability, and sustainability, presents significant challenges for managing osteoporosis in developing economies (24). In Myanmar, as another developing country, the ASMR is higher than the global average, but there is an overall fluctuating downward trend. According to early reports from Myanmar in 2012, osteoporosis was still an underrecognized health issue in the country, with a limited availability and accessibility of standard diagnostic tools and medications. Diagnostic and treatment inadequacies were widespread in clinical practice (25). In 2023, Myanmar released the “Myanmar Clinical Practice Guideline for the Management of Osteoporosis and Fragility Fractures,” aiming to fully implement strategies for the prevention and treatment of osteoporosis, thereby advancing the management of osteoporosis in the country (26).

To further explore the driving factors behind the above changes, we conducted a correlation analysis and frontier analysis between SDI, ASMR, and ASDR. From a regional development perspective, a significant association between SDI and both ASMR and ASDR burdens was observed. In countries with low to middle SDI (e.g., Vietnam, Cambodia), despite the economic improvements, the burden of LBMD-related diseases continues to rise, reflecting the uneven distribution of public health resources and medical services, which limits the ability of relevant regions to effectively prevent, screen, and treat diseases, thereby exacerbating the burden. In addition, in countries with low SDI, micronutrient deficiencies and rapid population aging also serve as significant drivers of the increasing disease burden. Deficiencies in micronutrients, such as inadequate calcium and vitamin D intake, impair bone health and elevate the risk of low bone mineral density. Meanwhile, the rapid aging of the population further escalates the overall burden of LBMD and related fall events. In contrast, high-SDI countries (e.g., Singapore) exhibit relatively low and stable mortality and DALY rates. It is noteworthy that despite significant improvements in LBMD-related burdens in many high-income countries, some high-SDI nations, such as Brunei, still show a relatively high burden. This trend may be influenced by multiple factors, including population-level lifestyle characteristics, such as nutritional habits affecting bone health, as well as system-level challenges, such as the possibility that preventive healthcare services for osteoporosis and fall control may not be fully implemented even in high-income countries. In addition, differences in data collection and reporting quality may also partly account for the observed increase in the disease burden. We conducted frontier analysis to investigate the potential for further improvement in managing the LBMD fall burden across different countries. Unlike traditional correlation analysis, frontier analysis identifies the theoretical minimum disease burden a country can achieve at its SDI level. The results of the frontier analysis revealed that, despite its lower SDI, Laos has demonstrated remarkable capability in controlling the burden of LBMD-related falls. Over the past two decades, Laos has significantly reduced poverty and hunger and made notable progress in education and health outcomes. The government of Laos has emphasized the need for healthcare system reform to provide high-quality health services and address the growing and evolving demands. To this end, it has formulated the “2013–2025 Health Sector Reform Strategy” (27). Our frontier analysis further demonstrated that Singapore has achieved the best practice in managing mortality related to falls associated with LBMD. The country boasts a comprehensive public health policy, intervention measures, and healthcare system focused on fall prevention and osteoporosis management. For instance, the Ministry of Health (MOH) in Singapore has established a dedicated fall prevention grant aimed at promoting translational research and innovation. This initiative facilitates the accurate and cost-effective identification of high-risk elderly populations, reduces their fall risk—including the effective prevention of recurrent falls—and ensures that these elderly individuals can live safely at home (i.e., non-institutionalized care) (28). Moreover, since July 2023, Singapore has implemented the “Healthier SG” national strategy, which represents a nationwide preventive healthcare system. This strategy marks a significant shift towards a more balanced healthcare framework, aiming to optimize the health and well-being of the population (29). At the same time, the results also indicate that high-SDI countries such as Singapore exemplify how comprehensive preventive strategies can be successfully integrated into national health systems, while low-SDI countries such as Laos demonstrate that targeted reforms and the efficient use of limited resources can still lead to substantial progress.

In the analysis of different age groups, the burden among individuals aged 80 and above was particularly prominent. This group not only faces a higher mortality rate but also experiences significant long-term pressure on the healthcare system due to disabilities resulting from falls. This phenomenon is closely related to LBMD as an age-associated disease characteristic. Studies indicate that for elderly individuals under the age of 70, fall risks are mainly influenced by external factors, such as ill-fitting shoes, insufficient lighting, and slippery floors. However, for the elderly aged 80 and above, the primary causes of falls include osteoporosis, sarcopenia, sleep disorders, multiple comorbidities, and frailty, all of which lead to a decline in intrinsic abilities and functional capacity (14). During the aging process, imbalances in bone remodeling, increased apoptosis of bone cells, and a decline in bone mass and mechanical properties significantly elevate the risk of fractures (30). Studies have shown that falls are the direct cause of 95% of hip fractures, with 20% of these leading to death (31–33). Despite the serious consequences of falls, they are often regarded as an inevitable part of aging. However, many falls are actually preventable. Therefore, strengthening health management for the elderly, particularly for those with LBMD and the elderly population, should be a key focus of future public health policies aimed at fall prevention. Besides the LBMD, fall risk in older adults may also be closely associated with other factors such as the lack of physical activity, cognitive decline, and environmental safety. First, insufficient physical activity is a key contributor to fall risk. Engaging in appropriate physical exercise can enhance muscle strength and improve balance, effectively reducing the likelihood of falls (34). Second, cognitive decline is another critical factor influencing fall risk. Impaired cognitive function may weaken environmental awareness, thereby increasing susceptibility to falls. Notably, in older adults, cognitive decline has been significantly associated with fall incidence (35). Third, environmental safety also plays a crucial role in fall prevention. Evidence indicates that the location of falls varies across age groups, with older adults more likely to fall at home (36, 37). Household hazards such as obstacles and poor lighting conditions can elevate fall risk (38). Therefore, improving environmental safety is an essential preventive measure. For older adults, a multidimensional intervention strategy—including systematic osteoporosis management, targeted rehabilitation training, and adaptive environmental modifications—can reduce fall risk and improve overall quality of life. In addition to age, sex differences merit careful consideration: the patterns of disease burden following falls appear to differ between men and women. Women are more likely to experience long-term functional impairment and elevated DALY rates, largely attributable to osteoporosis, sarcopenia, and multiple comorbidities, with these effects potentially compounded by limited access to social support resources (39, 40). By contrast, the burden observed in men may be more closely associated with comorbidity profiles, injury patterns, and variations in healthcare utilization (41). Collectively, these physiological and social determinants provide a plausible explanation for the observed sex disparities.

Projections based on the BAPC model suggest that global, Chinese, and most ASEAN ASMR and ASDR burdens will decline by 2035. However, countries like the Philippines and Brunei may see a slight rise in ASMR, while Cambodia and Thailand are expected to experience an increase in DALY burdens. These trends may stem from factors such as population aging, limited healthcare resources, rising health inequities, and growing chronic disease burdens. Future public health policies should prioritize strengthening prevention, early screening, and bone health education for the elderly, particularly in low-income countries.

This study has several limitations. First, although the GBD methodology is widely regarded as reliable, its estimates depend on national health records, which vary in quality and availability. In data-scarce regions, the model relies on covariates and data from wealthier areas, introducing uncertainty. In some countries with underdeveloped monitoring systems, falls and osteoporosis may be underreported or misclassified, which could be related to limited diagnostic capacity, insufficient surveillance infrastructure, and factors influencing healthcare-seeking behavior, which lead to data inaccuracies. Second, while the BAPC model offers flexible parameter estimation, its reliance on assumptions and sensitivity to data quality may lead to instability or inaccuracies, particularly when dealing with noisy data or small sample sizes. Projections for 2035, derived from historical trajectory analysis and methodological frameworks, may not fully account for the complexity of emerging risk factors, including dynamic policy shifts, socio-cultural changes, and unforeseen public health crises, all of which could significantly influence disease epidemiological trends. Finally, the sample size in our frontier analysis is limited, and applying this approach to China and the ASEAN countries may have constraints. In particular, the explanation of “efficiency differences” relies on simplified assumptions, and more detailed statistical data are needed to strengthen the interpretation of frontier results. Future studies should refine the methodology and incorporate richer datasets to improve reliability.

Despite the above limitations, this study provides novel and valuable evidence; it is the first to describe the burden of falls associated with LBMD in global, Chinese and ASEAN populations from 1990 to 2021, and to project future trends up to 2035, providing key insights for the formulation of national health policies, long-term health investments, strategic planning, and priority-setting. Furthermore, this study highlights the disparities in fall burden across different countries and populations, particularly the unique challenges faced by regions with low SDI and elderly groups.

## 5 Conclusion

Our analyses based on joinpoint, frontier and BAPC models indicated that the burden of falls attributable to low bone density remains substantial worldwide, with China and ASEAN countries contributing a considerable proportion. The impact is especially pronounced in low-income settings and among adults aged 80 years and older, particularly women. These findings emphasize the importance of prioritizing fall prevention and bone health strategies, optimizing healthcare resource allocation, and reinforcing policy interventions to effectively reduce disparities and mitigate this persistent global health challenge.

## Data Availability

The original contributions presented in the study are included in the article/Supplementary material, further inquiries can be directed to the corresponding authors.
