# Supplementary material for: Burden of falls associated with low bone density globally, in China and ASEAN countries: 1990–2021 trends and projections to 2035
Source: Front Med (Lausanne). 2025 Oct 20;12:1638057. doi: 10.3389/fmed.2025.1638057 (PMC12580195; doi:10.3389/fmed.2025.1638057)
Supplement: Supplementary file 1 [file Table_1.docx]

Supplementary Material

**Table S1: Prediction results of BAPC mortality in the world, China and ASEAN countries to 2035**

|  | **Val** | **SD** | **Time** | **Group** | **Low_50** | **Up_50** | **Low_60** | **Up_60** | **Low_70** | **Up_70** | **Low_80** | **Up_80** | **Low_95** | **Up_95** |
| --- | --- | --- | --- | --- | --- | --- | --- | --- | --- | --- | --- | --- | --- | --- |
| **Global** | | | | | | | | | | | | | | |
| 1 | 4.156445 | 0.012429 | 1990 | ASR | 4.148068 | 4.164822 | 4.145993 | 4.166898 | 4.143569 | 4.169322 | 4.140512 | 4.172379 | 4.132085 | 4.180806 |
| 2 | 4.130639 | 0.011992 | 1991 | ASR | 4.122557 | 4.138721 | 4.120554 | 4.140724 | 4.118216 | 4.143062 | 4.115266 | 4.146012 | 4.107135 | 4.154143 |
| 3 | 4.111768 | 0.011715 | 1992 | ASR | 4.103873 | 4.119664 | 4.101916 | 4.121621 | 4.099632 | 4.123905 | 4.09675 | 4.126787 | 4.088807 | 4.13473 |
| 4 | 4.104643 | 0.011495 | 1993 | ASR | 4.096895 | 4.11239 | 4.094975 | 4.11431 | 4.092734 | 4.116551 | 4.089906 | 4.119379 | 4.082113 | 4.127173 |
| 5 | 4.079755 | 0.011256 | 1994 | ASR | 4.072168 | 4.087341 | 4.070288 | 4.089221 | 4.068093 | 4.091416 | 4.065324 | 4.094185 | 4.057693 | 4.101817 |
| 6 | 4.123562 | 0.011139 | 1995 | ASR | 4.116054 | 4.13107 | 4.114194 | 4.13293 | 4.112022 | 4.135102 | 4.109281 | 4.137842 | 4.101729 | 4.145395 |
| 7 | 4.085466 | 0.010909 | 1996 | ASR | 4.078113 | 4.092818 | 4.076291 | 4.09464 | 4.074164 | 4.096767 | 4.071481 | 4.09945 | 4.064085 | 4.106846 |
| 8 | 4.001256 | 0.010619 | 1997 | ASR | 3.994099 | 4.008413 | 3.992326 | 4.010187 | 3.990255 | 4.012257 | 3.987643 | 4.01487 | 3.980444 | 4.022069 |
| 9 | 3.995233 | 0.010443 | 1998 | ASR | 3.988194 | 4.002272 | 3.98645 | 4.004016 | 3.984414 | 4.006052 | 3.981845 | 4.008621 | 3.974764 | 4.015702 |
| 10 | 3.957814 | 0.010232 | 1999 | ASR | 3.950918 | 3.96471 | 3.949209 | 3.966419 | 3.947214 | 3.968414 | 3.944697 | 3.970931 | 3.93776 | 3.977868 |
| 11 | 3.974192 | 0.010096 | 2000 | ASR | 3.967388 | 3.980997 | 3.965702 | 3.982683 | 3.963733 | 3.984652 | 3.961249 | 3.987135 | 3.954404 | 3.99398 |
| 12 | 4.060149 | 0.010054 | 2001 | ASR | 4.053372 | 4.066925 | 4.051693 | 4.068604 | 4.049733 | 4.070564 | 4.04726 | 4.073037 | 4.040443 | 4.079854 |
| 13 | 4.132585 | 0.009999 | 2002 | ASR | 4.125846 | 4.139325 | 4.124177 | 4.140994 | 4.122227 | 4.142944 | 4.119767 | 4.145404 | 4.112988 | 4.152183 |
| 14 | 4.234802 | 0.00999 | 2003 | ASR | 4.228069 | 4.241535 | 4.226401 | 4.243203 | 4.224453 | 4.245151 | 4.221995 | 4.247609 | 4.215222 | 4.254382 |
| 15 | 4.250815 | 0.009862 | 2004 | ASR | 4.244168 | 4.257462 | 4.242521 | 4.259109 | 4.240598 | 4.261032 | 4.238172 | 4.263458 | 4.231485 | 4.270145 |
| 16 | 4.239514 | 0.009683 | 2005 | ASR | 4.232987 | 4.24604 | 4.23137 | 4.247657 | 4.229482 | 4.249545 | 4.2271 | 4.251927 | 4.220535 | 4.258492 |
| 17 | 4.205111 | 0.009478 | 2006 | ASR | 4.198723 | 4.211499 | 4.19714 | 4.213082 | 4.195292 | 4.21493 | 4.19296 | 4.217262 | 4.186534 | 4.223688 |
| 18 | 4.226504 | 0.009342 | 2007 | ASR | 4.220208 | 4.2328 | 4.218648 | 4.23436 | 4.216826 | 4.236182 | 4.214528 | 4.23848 | 4.208194 | 4.244814 |
| 19 | 4.240057 | 0.009194 | 2008 | ASR | 4.23386 | 4.246253 | 4.232325 | 4.247788 | 4.230532 | 4.249581 | 4.22827 | 4.251843 | 4.222037 | 4.258076 |
| 20 | 4.16654 | 0.008941 | 2009 | ASR | 4.160514 | 4.172566 | 4.159021 | 4.174059 | 4.157277 | 4.175803 | 4.155078 | 4.178002 | 4.149016 | 4.184064 |
| 21 | 4.142392 | 0.008743 | 2010 | ASR | 4.136499 | 4.148285 | 4.135039 | 4.149745 | 4.133334 | 4.15145 | 4.131183 | 4.153601 | 4.125255 | 4.159529 |
| 22 | 4.133066 | 0.008563 | 2011 | ASR | 4.127295 | 4.138838 | 4.125865 | 4.140268 | 4.124195 | 4.141938 | 4.122088 | 4.144045 | 4.116282 | 4.14985 |
| 23 | 4.142684 | 0.008413 | 2012 | ASR | 4.137014 | 4.148354 | 4.135609 | 4.149759 | 4.133968 | 4.1514 | 4.131898 | 4.15347 | 4.126194 | 4.159174 |
| 24 | 4.284625 | 0.008422 | 2013 | ASR | 4.278948 | 4.290301 | 4.277542 | 4.291708 | 4.275899 | 4.29335 | 4.273827 | 4.295422 | 4.268117 | 4.301132 |
| 25 | 4.375992 | 0.008378 | 2014 | ASR | 4.370345 | 4.381639 | 4.368946 | 4.383038 | 4.367312 | 4.384672 | 4.365251 | 4.386733 | 4.359571 | 4.392414 |
| 26 | 4.338052 | 0.008184 | 2015 | ASR | 4.332536 | 4.343568 | 4.331169 | 4.344934 | 4.329573 | 4.34653 | 4.32756 | 4.348543 | 4.322012 | 4.354092 |
| 27 | 4.324798 | 0.008019 | 2016 | ASR | 4.319393 | 4.330202 | 4.318054 | 4.331541 | 4.31649 | 4.333105 | 4.314517 | 4.335078 | 4.309081 | 4.340514 |
| 28 | 4.32781 | 0.007877 | 2017 | ASR | 4.322501 | 4.33312 | 4.321186 | 4.334435 | 4.31965 | 4.335971 | 4.317712 | 4.337909 | 4.312371 | 4.343249 |
| 29 | 4.30095 | 0.007711 | 2018 | ASR | 4.295753 | 4.306147 | 4.294465 | 4.307435 | 4.292961 | 4.308939 | 4.291064 | 4.310836 | 4.285836 | 4.316064 |
| 30 | 4.27909 | 0.007556 | 2019 | ASR | 4.273997 | 4.284183 | 4.272735 | 4.285444 | 4.271262 | 4.286918 | 4.269403 | 4.288777 | 4.26428 | 4.2939 |
| 31 | 4.227389 | 0.007399 | 2020 | ASR | 4.222401 | 4.232376 | 4.221166 | 4.233611 | 4.219723 | 4.235054 | 4.217903 | 4.236875 | 4.212886 | 4.241891 |
| 32 | 4.159292 | 0.007279 | 2021 | ASR | 4.154386 | 4.164198 | 4.15317 | 4.165414 | 4.15175 | 4.166833 | 4.14996 | 4.168624 | 4.145024 | 4.17356 |
| 33 | 4.201717 | 0.061366 | 2022 | ASR | 4.160357 | 4.243078 | 4.150109 | 4.253326 | 4.138143 | 4.265292 | 4.123047 | 4.280388 | 4.081441 | 4.321994 |
| 34 | 4.188115 | 0.075039 | 2023 | ASR | 4.137539 | 4.238691 | 4.125008 | 4.251223 | 4.110375 | 4.265856 | 4.091915 | 4.284315 | 4.041039 | 4.335191 |
| 35 | 4.172049 | 0.08699 | 2024 | ASR | 4.113418 | 4.23068 | 4.09889 | 4.245207 | 4.081927 | 4.26217 | 4.060528 | 4.283569 | 4.001549 | 4.342548 |
| 36 | 4.153988 | 0.097806 | 2025 | ASR | 4.088066 | 4.219909 | 4.071733 | 4.236243 | 4.05266 | 4.255315 | 4.0286 | 4.279376 | 3.962287 | 4.345688 |
| 37 | 4.134957 | 0.107839 | 2026 | ASR | 4.062274 | 4.20764 | 4.044265 | 4.225649 | 4.023236 | 4.246678 | 3.996708 | 4.273206 | 3.923593 | 4.346321 |
| 38 | 4.114786 | 0.117319 | 2027 | ASR | 4.035713 | 4.193859 | 4.016121 | 4.213451 | 3.993243 | 4.236328 | 3.964383 | 4.265189 | 3.884841 | 4.344731 |
| 39 | 4.092471 | 0.126272 | 2028 | ASR | 4.007364 | 4.177579 | 3.986276 | 4.198666 | 3.961653 | 4.223289 | 3.93059 | 4.254352 | 3.844978 | 4.339965 |
| 40 | 4.067791 | 0.134749 | 2029 | ASR | 3.97697 | 4.158611 | 3.954467 | 4.181114 | 3.928191 | 4.20739 | 3.895043 | 4.240539 | 3.803684 | 4.331898 |
| 41 | 4.041555 | 0.142809 | 2030 | ASR | 3.945302 | 4.137808 | 3.921453 | 4.161657 | 3.893605 | 4.189505 | 3.858474 | 4.224636 | 3.761649 | 4.321461 |
| 42 | 4.014308 | 0.150523 | 2031 | ASR | 3.912856 | 4.115761 | 3.887718 | 4.140898 | 3.858366 | 4.17025 | 3.821337 | 4.207279 | 3.719283 | 4.309334 |
| 43 | 3.985616 | 0.157953 | 2032 | ASR | 3.879156 | 4.092076 | 3.852778 | 4.118454 | 3.821977 | 4.149255 | 3.783121 | 4.188112 | 3.676029 | 4.295203 |
| 44 | 3.955261 | 0.165068 | 2033 | ASR | 3.844005 | 4.066517 | 3.816439 | 4.094084 | 3.78425 | 4.126272 | 3.743644 | 4.166879 | 3.631727 | 4.278795 |
| 45 | 3.923815 | 0.1719 | 2034 | ASR | 3.807954 | 4.039676 | 3.779247 | 4.068383 | 3.745727 | 4.101904 | 3.703439 | 4.144191 | 3.586891 | 4.26074 |
| 46 | 3.892379 | 0.178501 | 2035 | ASR | 3.77207 | 4.012689 | 3.74226 | 4.042499 | 3.707452 | 4.077307 | 3.663541 | 4.121218 | 3.542517 | 4.242242 |
| **China** | | | | | | | | | | | | | | |
| 1 | 3.673457 | 0.036728 | 1990 | ASR | 3.648702 | 3.698212 | 3.642569 | 3.704345 | 3.635407 | 3.711507 | 3.626372 | 3.720542 | 3.60147 | 3.745444 |
| 2 | 3.619887 | 0.03392 | 1991 | ASR | 3.597024 | 3.642749 | 3.59136 | 3.648414 | 3.584745 | 3.655028 | 3.576401 | 3.663372 | 3.553403 | 3.68637 |
| 3 | 3.569606 | 0.032286 | 1992 | ASR | 3.547846 | 3.591367 | 3.542454 | 3.596759 | 3.536158 | 3.603054 | 3.528216 | 3.610997 | 3.506327 | 3.632886 |
| 4 | 3.525476 | 0.030966 | 1993 | ASR | 3.504605 | 3.546347 | 3.499434 | 3.551519 | 3.493396 | 3.557557 | 3.485778 | 3.565175 | 3.464783 | 3.58617 |
| 5 | 3.492757 | 0.029851 | 1994 | ASR | 3.472637 | 3.512877 | 3.467652 | 3.517862 | 3.461831 | 3.523683 | 3.454487 | 3.531026 | 3.434248 | 3.551265 |
| 6 | 3.457943 | 0.02884 | 1995 | ASR | 3.438504 | 3.477381 | 3.433688 | 3.482197 | 3.428064 | 3.487821 | 3.42097 | 3.494916 | 3.401416 | 3.514469 |
| 7 | 3.429727 | 0.027979 | 1996 | ASR | 3.410869 | 3.448584 | 3.406196 | 3.453257 | 3.40074 | 3.458713 | 3.393857 | 3.465596 | 3.374888 | 3.484566 |
| 8 | 3.430545 | 0.027342 | 1997 | ASR | 3.412116 | 3.448974 | 3.40755 | 3.45354 | 3.402218 | 3.458872 | 3.395492 | 3.465598 | 3.376954 | 3.484136 |
| 9 | 3.473774 | 0.026972 | 1998 | ASR | 3.455595 | 3.491953 | 3.45109 | 3.496458 | 3.445831 | 3.501717 | 3.439195 | 3.508352 | 3.420908 | 3.52664 |
| 10 | 3.5472 | 0.026782 | 1999 | ASR | 3.529149 | 3.565251 | 3.524676 | 3.569723 | 3.519454 | 3.574946 | 3.512865 | 3.581534 | 3.494707 | 3.599692 |
| 11 | 3.71546 | 0.027027 | 2000 | ASR | 3.697244 | 3.733675 | 3.69273 | 3.738189 | 3.68746 | 3.743459 | 3.680812 | 3.750108 | 3.662488 | 3.768432 |
| 12 | 3.97531 | 0.027653 | 2001 | ASR | 3.956672 | 3.993948 | 3.952054 | 3.998566 | 3.946662 | 4.003959 | 3.939859 | 4.010761 | 3.92111 | 4.02951 |
| 13 | 4.210036 | 0.028157 | 2002 | ASR | 4.191059 | 4.229014 | 4.186357 | 4.233716 | 4.180866 | 4.239207 | 4.173939 | 4.246133 | 4.154849 | 4.265224 |
| 14 | 4.526192 | 0.029165 | 2003 | ASR | 4.506535 | 4.545849 | 4.501664 | 4.550719 | 4.495977 | 4.556406 | 4.488803 | 4.563581 | 4.469029 | 4.583355 |
| 15 | 4.77747 | 0.029722 | 2004 | ASR | 4.757437 | 4.797503 | 4.752473 | 4.802466 | 4.746678 | 4.808262 | 4.739366 | 4.815574 | 4.719214 | 4.835726 |
| 16 | 4.800379 | 0.029268 | 2005 | ASR | 4.780653 | 4.820106 | 4.775765 | 4.824994 | 4.770058 | 4.830701 | 4.762858 | 4.837901 | 4.743014 | 4.857745 |
| 17 | 4.520298 | 0.027469 | 2006 | ASR | 4.501784 | 4.538812 | 4.497197 | 4.5434 | 4.49184 | 4.548756 | 4.485083 | 4.555513 | 4.466459 | 4.574137 |
| 18 | 4.393581 | 0.026267 | 2007 | ASR | 4.375877 | 4.411285 | 4.37149 | 4.415671 | 4.366368 | 4.420793 | 4.359907 | 4.427255 | 4.342098 | 4.445064 |
| 19 | 4.330055 | 0.025251 | 2008 | ASR | 4.313035 | 4.347074 | 4.308818 | 4.351291 | 4.303894 | 4.356215 | 4.297682 | 4.362427 | 4.280562 | 4.379547 |
| 20 | 4.249833 | 0.024228 | 2009 | ASR | 4.233503 | 4.266162 | 4.229457 | 4.270208 | 4.224733 | 4.274933 | 4.218773 | 4.280893 | 4.202347 | 4.297319 |
| 21 | 4.159605 | 0.023202 | 2010 | ASR | 4.143966 | 4.175243 | 4.140092 | 4.179118 | 4.135567 | 4.183642 | 4.129859 | 4.18935 | 4.114128 | 4.205081 |
| 22 | 4.068342 | 0.02222 | 2011 | ASR | 4.053366 | 4.083319 | 4.049655 | 4.08703 | 4.045322 | 4.091363 | 4.039856 | 4.096829 | 4.02479 | 4.111894 |
| 23 | 3.950031 | 0.021183 | 2012 | ASR | 3.935754 | 3.964309 | 3.932216 | 3.967847 | 3.928085 | 3.971977 | 3.922874 | 3.977188 | 3.908512 | 3.991551 |
| 24 | 3.916322 | 0.020423 | 2013 | ASR | 3.902557 | 3.930087 | 3.899146 | 3.933498 | 3.895164 | 3.937481 | 3.89014 | 3.942505 | 3.876293 | 3.956352 |
| 25 | 3.87977 | 0.0197 | 2014 | ASR | 3.866492 | 3.893048 | 3.863202 | 3.896337 | 3.859361 | 3.900179 | 3.854515 | 3.905025 | 3.841158 | 3.918381 |
| 26 | 3.841259 | 0.019004 | 2015 | ASR | 3.82845 | 3.854068 | 3.825277 | 3.857242 | 3.821571 | 3.860948 | 3.816896 | 3.865623 | 3.804011 | 3.878508 |
| 27 | 3.852968 | 0.018476 | 2016 | ASR | 3.840516 | 3.865421 | 3.83743 | 3.868506 | 3.833828 | 3.872109 | 3.829283 | 3.876654 | 3.816756 | 3.889181 |
| 28 | 3.830992 | 0.017912 | 2017 | ASR | 3.81892 | 3.843065 | 3.815929 | 3.846056 | 3.812436 | 3.849549 | 3.80803 | 3.853955 | 3.795886 | 3.866099 |
| 29 | 3.75175 | 0.017234 | 2018 | ASR | 3.740134 | 3.763366 | 3.737256 | 3.766244 | 3.733895 | 3.769605 | 3.729656 | 3.773845 | 3.717971 | 3.78553 |
| 30 | 3.695939 | 0.016637 | 2019 | ASR | 3.684725 | 3.707152 | 3.681947 | 3.709931 | 3.678702 | 3.713175 | 3.67461 | 3.717268 | 3.66333 | 3.728548 |
| 31 | 3.651669 | 0.016107 | 2020 | ASR | 3.640813 | 3.662525 | 3.638123 | 3.665215 | 3.634982 | 3.668356 | 3.63102 | 3.672319 | 3.620099 | 3.683239 |
| 32 | 3.58129 | 0.015662 | 2021 | ASR | 3.570734 | 3.591846 | 3.568119 | 3.594462 | 3.565064 | 3.597516 | 3.561212 | 3.601369 | 3.550593 | 3.611987 |
| 33 | 3.593423 | 0.11056 | 2022 | ASR | 3.518906 | 3.667941 | 3.500443 | 3.686404 | 3.478884 | 3.707963 | 3.451686 | 3.735161 | 3.376726 | 3.810121 |
| 34 | 3.57095 | 0.148705 | 2023 | ASR | 3.470723 | 3.671178 | 3.445889 | 3.696011 | 3.416892 | 3.725009 | 3.380311 | 3.76159 | 3.279489 | 3.862412 |
| 35 | 3.548187 | 0.179769 | 2024 | ASR | 3.427022 | 3.669351 | 3.397001 | 3.699372 | 3.361946 | 3.734427 | 3.317722 | 3.778651 | 3.195839 | 3.900534 |
| 36 | 3.525061 | 0.206911 | 2025 | ASR | 3.385603 | 3.664519 | 3.351049 | 3.699073 | 3.310702 | 3.739421 | 3.259802 | 3.790321 | 3.119516 | 3.930606 |
| 37 | 3.501458 | 0.231478 | 2026 | ASR | 3.345442 | 3.657475 | 3.306785 | 3.696132 | 3.261647 | 3.74127 | 3.204703 | 3.798214 | 3.047761 | 3.955156 |
| 38 | 3.477416 | 0.254199 | 2027 | ASR | 3.306086 | 3.648746 | 3.263634 | 3.691197 | 3.214066 | 3.740766 | 3.151533 | 3.803299 | 2.979186 | 3.975645 |
| 39 | 3.452916 | 0.275433 | 2028 | ASR | 3.267274 | 3.638558 | 3.221276 | 3.684555 | 3.167567 | 3.738265 | 3.09981 | 3.806021 | 2.913066 | 3.992765 |
| 40 | 3.428168 | 0.295454 | 2029 | ASR | 3.229032 | 3.627304 | 3.179691 | 3.676645 | 3.122078 | 3.734258 | 3.049396 | 3.80694 | 2.849079 | 4.007258 |
| 41 | 3.403131 | 0.314458 | 2030 | ASR | 3.191187 | 3.615075 | 3.138672 | 3.66759 | 3.077353 | 3.728909 | 2.999996 | 3.806266 | 2.786794 | 4.019468 |
| 42 | 3.377563 | 0.332578 | 2031 | ASR | 3.153406 | 3.60172 | 3.097865 | 3.657261 | 3.033013 | 3.722114 | 2.951199 | 3.803928 | 2.725711 | 4.029415 |
| 43 | 3.351348 | 0.349918 | 2032 | ASR | 3.115503 | 3.587192 | 3.057067 | 3.645629 | 2.988833 | 3.713862 | 2.902753 | 3.799942 | 2.665509 | 4.037186 |
| 44 | 3.324656 | 0.366528 | 2033 | ASR | 3.077617 | 3.571696 | 3.016407 | 3.632906 | 2.944934 | 3.704379 | 2.854768 | 3.794544 | 2.606262 | 4.04305 |
| 45 | 3.297825 | 0.382489 | 2034 | ASR | 3.040027 | 3.555623 | 2.976151 | 3.619499 | 2.901566 | 3.694084 | 2.807473 | 3.788176 | 2.548146 | 4.047504 |
| 46 | 3.271063 | 0.397889 | 2035 | ASR | 3.002885 | 3.53924 | 2.936438 | 3.605687 | 2.85885 | 3.683276 | 2.760969 | 3.781156 | 2.4912 | 4.050925 |
| **Brunei Darussalam** | | | | | | | | | | | | | | |
| 1 | 0.007464 | 0.009497 | 1990 | ASR | 0.001062 | 0.013865 | -0.00052 | 0.015451 | -0.00238 | 0.017303 | -0.00471 | 0.019639 | -0.01115 | 0.026078 |
| 2 | 0.009455 | 0.011479 | 1991 | ASR | 0.001718 | 0.017192 | -0.0002 | 0.019109 | -0.00244 | 0.021347 | -0.00526 | 0.024171 | -0.01304 | 0.031954 |
| 3 | 0.01196 | 0.013842 | 1992 | ASR | 0.002631 | 0.02129 | 0.000319 | 0.023601 | -0.00238 | 0.0263 | -0.00579 | 0.029706 | -0.01517 | 0.03909 |
| 4 | 0.015106 | 0.016648 | 1993 | ASR | 0.003885 | 0.026327 | 0.001105 | 0.029107 | -0.00214 | 0.032354 | -0.00624 | 0.036449 | -0.01752 | 0.047737 |
| 5 | 0.019052 | 0.019971 | 1994 | ASR | 0.005592 | 0.032512 | 0.002257 | 0.035847 | -0.00164 | 0.039741 | -0.00655 | 0.044654 | -0.02009 | 0.058194 |
| 6 | 0.023993 | 0.023891 | 1995 | ASR | 0.00789 | 0.040095 | 0.0039 | 0.044085 | -0.00076 | 0.048744 | -0.00664 | 0.054621 | -0.02283 | 0.070819 |
| 7 | 0.030171 | 0.0285 | 1996 | ASR | 0.010962 | 0.049379 | 0.006202 | 0.054139 | 0.000645 | 0.059696 | -0.00637 | 0.066707 | -0.02569 | 0.08603 |
| 8 | 0.037873 | 0.033887 | 1997 | ASR | 0.015034 | 0.060713 | 0.009375 | 0.066372 | 0.002767 | 0.07298 | -0.00557 | 0.081316 | -0.02854 | 0.104291 |
| 9 | 0.04745 | 0.040147 | 1998 | ASR | 0.020391 | 0.074509 | 0.013687 | 0.081214 | 0.005858 | 0.089043 | -0.00402 | 0.098919 | -0.03124 | 0.126139 |
| 10 | 0.05934 | 0.04739 | 1999 | ASR | 0.027399 | 0.09128 | 0.019485 | 0.099194 | 0.010244 | 0.108435 | -0.00141 | 0.120093 | -0.03354 | 0.152223 |
| 11 | 0.074074 | 0.055728 | 2000 | ASR | 0.036513 | 0.111635 | 0.027207 | 0.120941 | 0.01634 | 0.131808 | 0.002631 | 0.145517 | -0.03515 | 0.183301 |
| 12 | 0.092299 | 0.065272 | 2001 | ASR | 0.048305 | 0.136292 | 0.037405 | 0.147193 | 0.024677 | 0.159921 | 0.00862 | 0.175978 | -0.03563 | 0.220232 |
| 13 | 0.114761 | 0.0761 | 2002 | ASR | 0.06347 | 0.166053 | 0.050761 | 0.178762 | 0.035921 | 0.193601 | 0.017201 | 0.212322 | -0.0344 | 0.263918 |
| 14 | 0.142348 | 0.088273 | 2003 | ASR | 0.082852 | 0.201844 | 0.068111 | 0.216585 | 0.050898 | 0.233799 | 0.029183 | 0.255514 | -0.03067 | 0.315363 |
| 15 | 0.176161 | 0.101859 | 2004 | ASR | 0.107508 | 0.244814 | 0.090497 | 0.261824 | 0.070635 | 0.281687 | 0.045577 | 0.306744 | -0.02348 | 0.375805 |
| 16 | 0.217512 | 0.116924 | 2005 | ASR | 0.138705 | 0.296319 | 0.119179 | 0.315845 | 0.096379 | 0.338645 | 0.067616 | 0.367409 | -0.01166 | 0.446683 |
| 17 | 0.267965 | 0.133493 | 2006 | ASR | 0.17799 | 0.357939 | 0.155697 | 0.380232 | 0.129666 | 0.406263 | 0.096827 | 0.439102 | 0.006319 | 0.52961 |
| 18 | 0.329242 | 0.151478 | 2007 | ASR | 0.227146 | 0.431338 | 0.201849 | 0.456635 | 0.172311 | 0.486173 | 0.135048 | 0.523436 | 0.032346 | 0.626138 |
| 19 | 0.403316 | 0.170743 | 2008 | ASR | 0.288234 | 0.518397 | 0.25972 | 0.546911 | 0.226425 | 0.580206 | 0.184422 | 0.622209 | 0.068658 | 0.737973 |
| 20 | 0.49345 | 0.191702 | 2009 | ASR | 0.364243 | 0.622658 | 0.332229 | 0.654672 | 0.294847 | 0.692054 | 0.247688 | 0.739213 | 0.117714 | 0.869187 |
| 21 | 0.601363 | 0.213639 | 2010 | ASR | 0.45737 | 0.745356 | 0.421692 | 0.781034 | 0.380033 | 0.822693 | 0.327477 | 0.875249 | 0.18263 | 1.020096 |
| 22 | 0.731586 | 0.237698 | 2011 | ASR | 0.571377 | 0.891794 | 0.531682 | 0.931489 | 0.485331 | 0.97784 | 0.426857 | 1.036314 | 0.265698 | 1.197473 |
| 23 | 0.884736 | 0.263534 | 2012 | ASR | 0.707114 | 1.062359 | 0.663104 | 1.106369 | 0.611715 | 1.157758 | 0.546885 | 1.222587 | 0.368209 | 1.401264 |
| 24 | 1.065284 | 0.292319 | 2013 | ASR | 0.868261 | 1.262307 | 0.819443 | 1.311124 | 0.762441 | 1.368127 | 0.69053 | 1.440037 | 0.492338 | 1.63823 |
| 25 | 1.277253 | 0.326126 | 2014 | ASR | 1.057444 | 1.497062 | 1.002981 | 1.551525 | 0.939387 | 1.61512 | 0.85916 | 1.695347 | 0.638046 | 1.91646 |
| 26 | 1.524967 | 0.368664 | 2015 | ASR | 1.276487 | 1.773446 | 1.21492 | 1.835013 | 1.143031 | 1.906903 | 1.052339 | 1.997594 | 0.802385 | 2.247548 |
| 27 | 1.817853 | 0.426711 | 2016 | ASR | 1.530249 | 2.105456 | 1.458989 | 2.176717 | 1.37578 | 2.259926 | 1.270809 | 2.364897 | 0.981499 | 2.654207 |
| 28 | 2.150903 | 0.504229 | 2017 | ASR | 1.811052 | 2.490753 | 1.726846 | 2.574959 | 1.628522 | 2.673284 | 1.504481 | 2.797324 | 1.162614 | 3.139191 |
| 29 | 2.53024 | 0.607676 | 2018 | ASR | 2.120666 | 2.939813 | 2.019184 | 3.041295 | 1.900687 | 3.159792 | 1.751199 | 3.30928 | 1.339195 | 3.721285 |
| 30 | 2.960835 | 0.743438 | 2019 | ASR | 2.459758 | 3.461913 | 2.335604 | 3.586067 | 2.190633 | 3.731037 | 2.007748 | 3.913923 | 1.503696 | 4.417974 |
| 31 | 3.443147 | 0.91816 | 2020 | ASR | 2.824307 | 4.061987 | 2.670974 | 4.21532 | 2.491933 | 4.394362 | 2.266066 | 4.620229 | 1.643553 | 5.242742 |
| 32 | 3.985638 | 1.140997 | 2021 | ASR | 3.216606 | 4.75467 | 3.02606 | 4.945216 | 2.803565 | 5.167711 | 2.52288 | 5.448396 | 1.749284 | 6.221992 |
| 33 | 4.614813 | 1.443001 | 2022 | ASR | 3.64223 | 5.587396 | 3.401249 | 5.828377 | 3.119864 | 6.109762 | 2.764886 | 6.46474 | 1.786531 | 7.443095 |
| 34 | 5.286148 | 1.792355 | 2023 | ASR | 4.078101 | 6.494196 | 3.778778 | 6.793519 | 3.429268 | 7.143028 | 2.988349 | 7.583948 | 1.773132 | 8.799164 |
| 35 | 6.015553 | 2.210712 | 2024 | ASR | 4.525533 | 7.505573 | 4.156344 | 7.874762 | 3.725255 | 8.305851 | 3.18142 | 8.849687 | 1.682557 | 10.34855 |
| 36 | 6.8024 | 2.706238 | 2025 | ASR | 4.978396 | 8.626405 | 4.526454 | 9.078346 | 3.998737 | 9.606063 | 3.333003 | 10.2718 | 1.498173 | 12.10663 |
| 37 | 7.64381 | 3.287183 | 2026 | ASR | 5.428249 | 9.859372 | 4.879289 | 10.40833 | 4.238289 | 11.04933 | 3.429642 | 11.85798 | 1.200932 | 14.08669 |
| 38 | 8.529507 | 3.957751 | 2027 | ASR | 5.861983 | 11.19703 | 5.201038 | 11.85798 | 4.429277 | 12.62974 | 3.45567 | 13.60334 | 0.772315 | 16.2867 |
| 39 | 9.442745 | 4.715157 | 2028 | ASR | 6.264729 | 12.62076 | 5.477297 | 13.40819 | 4.557842 | 14.32765 | 3.397913 | 15.48758 | 0.201036 | 18.68445 |
| 40 | 10.36777 | 5.556722 | 2029 | ASR | 6.622542 | 14.113 | 5.69457 | 15.04098 | 4.611009 | 16.12454 | 3.244055 | 17.49149 | -0.5234 | 21.25895 |
| 41 | 11.29083 | 6.480514 | 2030 | ASR | 6.922967 | 15.6587 | 5.840721 | 16.74095 | 4.577021 | 18.00465 | 2.982814 | 19.59885 | -1.41097 | 23.99264 |
| 42 | 12.20055 | 7.484456 | 2031 | ASR | 7.156024 | 17.24507 | 5.90612 | 18.49498 | 4.446651 | 19.95444 | 2.605475 | 21.79562 | -2.46899 | 26.87008 |
| 43 | 13.08041 | 8.561062 | 2032 | ASR | 7.310254 | 18.85057 | 5.880557 | 20.28026 | 4.21115 | 21.94967 | 2.105129 | 24.05569 | -3.69927 | 29.86009 |
| 44 | 13.9102 | 9.694687 | 2033 | ASR | 7.375985 | 20.44442 | 5.756972 | 22.06344 | 3.866509 | 23.9539 | 1.481616 | 26.33879 | -5.09138 | 32.91179 |
| 45 | 14.67397 | 10.87238 | 2034 | ASR | 7.345985 | 22.00196 | 5.530298 | 23.81764 | 3.410183 | 25.93776 | 0.735578 | 28.61236 | -6.6359 | 35.98384 |
| 46 | 15.36056 | 12.08417 | 2035 | ASR | 7.215825 | 23.50529 | 5.197769 | 25.52334 | 2.841356 | 27.87976 | -0.13135 | 30.85246 | -8.32442 | 39.04553 |
| **Cambodia** | | | | | | | | | | | | | | |
| 1 | 8.23471 | 0.317667 | 1990 | ASR | 8.020603 | 8.448818 | 7.967552 | 8.501868 | 7.905607 | 8.563813 | 7.827461 | 8.641959 | 7.612083 | 8.857337 |
| 2 | 8.310168 | 0.287316 | 1991 | ASR | 8.116516 | 8.503819 | 8.068535 | 8.5518 | 8.012508 | 8.607827 | 7.941828 | 8.678507 | 7.747028 | 8.873307 |
| 3 | 8.373814 | 0.263666 | 1992 | ASR | 8.196103 | 8.551525 | 8.152071 | 8.595557 | 8.100656 | 8.646972 | 8.035795 | 8.711834 | 7.857029 | 8.890599 |
| 4 | 8.43509 | 0.245661 | 1993 | ASR | 8.269515 | 8.600666 | 8.228489 | 8.641691 | 8.180585 | 8.689595 | 8.120152 | 8.750028 | 7.953594 | 8.916586 |
| 5 | 8.486822 | 0.231773 | 1994 | ASR | 8.330607 | 8.643037 | 8.291901 | 8.681743 | 8.246706 | 8.726938 | 8.189689 | 8.783954 | 8.032548 | 8.941096 |
| 6 | 8.532331 | 0.221138 | 1995 | ASR | 8.383284 | 8.681378 | 8.346354 | 8.718309 | 8.303232 | 8.761431 | 8.248832 | 8.815831 | 8.0989 | 8.965762 |
| 7 | 8.567673 | 0.212926 | 1996 | ASR | 8.424161 | 8.711184 | 8.388602 | 8.746743 | 8.347082 | 8.788264 | 8.294702 | 8.840643 | 8.150338 | 8.985007 |
| 8 | 8.596343 | 0.206674 | 1997 | ASR | 8.457045 | 8.735642 | 8.42253 | 8.770156 | 8.382228 | 8.810458 | 8.331387 | 8.8613 | 8.191261 | 9.001425 |
| 9 | 8.621788 | 0.201986 | 1998 | ASR | 8.485649 | 8.757926 | 8.451918 | 8.791657 | 8.41253 | 8.831045 | 8.362842 | 8.880733 | 8.225896 | 9.017679 |
| 10 | 8.646067 | 0.198536 | 1999 | ASR | 8.512253 | 8.77988 | 8.479098 | 8.813036 | 8.440383 | 8.85175 | 8.391543 | 8.90059 | 8.256936 | 9.035198 |
| 11 | 8.667561 | 0.195991 | 2000 | ASR | 8.535463 | 8.799659 | 8.502733 | 8.83239 | 8.464514 | 8.870608 | 8.416301 | 8.918822 | 8.283419 | 9.051704 |
| 12 | 8.682414 | 0.194112 | 2001 | ASR | 8.551582 | 8.813246 | 8.519165 | 8.845662 | 8.481313 | 8.883514 | 8.433562 | 8.931266 | 8.301954 | 9.062874 |
| 13 | 8.695988 | 0.192796 | 2002 | ASR | 8.566044 | 8.825932 | 8.533847 | 8.858129 | 8.496252 | 8.895724 | 8.448824 | 8.943152 | 8.318108 | 9.073868 |
| 14 | 8.71297 | 0.191879 | 2003 | ASR | 8.583644 | 8.842297 | 8.5516 | 8.874341 | 8.514183 | 8.911757 | 8.466981 | 8.958959 | 8.336887 | 9.089053 |
| 15 | 8.735441 | 0.191208 | 2004 | ASR | 8.606567 | 8.864316 | 8.574635 | 8.896248 | 8.537349 | 8.933533 | 8.490312 | 8.980571 | 8.360673 | 9.11021 |
| 16 | 8.763684 | 0.190542 | 2005 | ASR | 8.635259 | 8.892109 | 8.603438 | 8.92393 | 8.566282 | 8.961085 | 8.519409 | 9.007959 | 8.390222 | 9.137146 |
| 17 | 8.804685 | 0.189753 | 2006 | ASR | 8.676792 | 8.932579 | 8.645103 | 8.964268 | 8.608101 | 9.001269 | 8.561422 | 9.047949 | 8.43277 | 9.176601 |
| 18 | 8.85113 | 0.188885 | 2007 | ASR | 8.723821 | 8.978438 | 8.692278 | 9.009982 | 8.655445 | 9.046815 | 8.608979 | 9.09328 | 8.480915 | 9.221344 |
| 19 | 8.905487 | 0.187948 | 2008 | ASR | 8.778811 | 9.032164 | 8.747423 | 9.063551 | 8.710773 | 9.100201 | 8.664538 | 9.146436 | 8.53711 | 9.273865 |
| 20 | 8.965038 | 0.187068 | 2009 | ASR | 8.838955 | 9.091122 | 8.807715 | 9.122362 | 8.771236 | 9.15884 | 8.725218 | 9.204859 | 8.598386 | 9.331691 |
| 21 | 9.030257 | 0.186333 | 2010 | ASR | 8.904669 | 9.155846 | 8.873551 | 9.186964 | 8.837216 | 9.223299 | 8.791378 | 9.269137 | 8.665044 | 9.395471 |
| 22 | 9.100607 | 0.185807 | 2011 | ASR | 8.975373 | 9.225842 | 8.944343 | 9.256871 | 8.908111 | 9.293104 | 8.862402 | 9.338813 | 8.736425 | 9.46479 |
| 23 | 9.170818 | 0.185571 | 2012 | ASR | 9.045743 | 9.295893 | 9.014753 | 9.326884 | 8.978566 | 9.36307 | 8.932916 | 9.408721 | 8.807098 | 9.534538 |
| 24 | 9.245804 | 0.185774 | 2013 | ASR | 9.120593 | 9.371016 | 9.089568 | 9.40204 | 9.053343 | 9.438266 | 9.007642 | 9.483966 | 8.881688 | 9.609921 |
| 25 | 9.319565 | 0.186422 | 2014 | ASR | 9.193916 | 9.445213 | 9.162784 | 9.476346 | 9.126431 | 9.512698 | 9.080571 | 9.558558 | 8.954177 | 9.684952 |
| 26 | 9.387511 | 0.187542 | 2015 | ASR | 9.261108 | 9.513914 | 9.229789 | 9.545234 | 9.193218 | 9.581804 | 9.147083 | 9.62794 | 9.019929 | 9.755093 |
| 27 | 9.446328 | 0.18914 | 2016 | ASR | 9.318848 | 9.573808 | 9.287261 | 9.605395 | 9.250379 | 9.642277 | 9.20385 | 9.688806 | 9.075613 | 9.817042 |
| 28 | 9.505262 | 0.191727 | 2017 | ASR | 9.376038 | 9.634486 | 9.34402 | 9.666504 | 9.306633 | 9.703891 | 9.259468 | 9.751056 | 9.129478 | 9.881046 |
| 29 | 9.546943 | 0.195128 | 2018 | ASR | 9.415426 | 9.678459 | 9.38284 | 9.711045 | 9.34479 | 9.749095 | 9.296789 | 9.797096 | 9.164492 | 9.929393 |
| 30 | 9.570929 | 0.200346 | 2019 | ASR | 9.435896 | 9.705962 | 9.402438 | 9.73942 | 9.36337 | 9.778488 | 9.314085 | 9.827773 | 9.17825 | 9.963607 |
| 31 | 9.592599 | 0.209941 | 2020 | ASR | 9.451099 | 9.734099 | 9.416039 | 9.769159 | 9.375101 | 9.810097 | 9.323455 | 9.861743 | 9.181116 | 10.00408 |
| 32 | 9.592467 | 0.227697 | 2021 | ASR | 9.438999 | 9.745935 | 9.400974 | 9.78396 | 9.356573 | 9.828361 | 9.300559 | 9.884375 | 9.14618 | 10.03875 |
| 33 | 9.558645 | 0.280346 | 2022 | ASR | 9.369691 | 9.747598 | 9.322873 | 9.794416 | 9.268206 | 9.849084 | 9.199241 | 9.918049 | 9.009166 | 10.10812 |
| 34 | 9.538681 | 0.315336 | 2023 | ASR | 9.326144 | 9.751218 | 9.273483 | 9.803879 | 9.211992 | 9.865369 | 9.13442 | 9.942942 | 8.920622 | 10.15674 |
| 35 | 9.515237 | 0.348325 | 2024 | ASR | 9.280466 | 9.750008 | 9.222296 | 9.808178 | 9.154372 | 9.876102 | 9.068684 | 9.96179 | 8.83252 | 10.19795 |
| 36 | 9.488496 | 0.37984 | 2025 | ASR | 9.232484 | 9.744509 | 9.16905 | 9.807942 | 9.094982 | 9.882011 | 9.001541 | 9.975452 | 8.744009 | 10.23298 |
| 37 | 9.458667 | 0.410247 | 2026 | ASR | 9.182161 | 9.735174 | 9.11365 | 9.803685 | 9.033652 | 9.883683 | 8.932731 | 9.984604 | 8.654583 | 10.26275 |
| 38 | 9.426083 | 0.439779 | 2027 | ASR | 9.129672 | 9.722495 | 9.056229 | 9.795938 | 8.970472 | 9.881695 | 8.862286 | 9.989881 | 8.564116 | 10.28805 |
| 39 | 9.391279 | 0.468527 | 2028 | ASR | 9.075492 | 9.707066 | 8.997249 | 9.78531 | 8.905886 | 9.876673 | 8.790628 | 9.99193 | 8.472967 | 10.30959 |
| 40 | 9.354689 | 0.496625 | 2029 | ASR | 9.019964 | 9.689414 | 8.937027 | 9.77235 | 8.840186 | 9.869192 | 8.718016 | 9.991362 | 8.381304 | 10.32807 |
| 41 | 9.316664 | 0.524232 | 2030 | ASR | 8.963332 | 9.669997 | 8.875785 | 9.757543 | 8.77356 | 9.859769 | 8.644599 | 9.98873 | 8.28917 | 10.34416 |
| 42 | 9.277546 | 0.5515 | 2031 | ASR | 8.905835 | 9.649257 | 8.813735 | 9.741358 | 8.706192 | 9.8489 | 8.570523 | 9.984569 | 8.196606 | 10.35849 |
| 43 | 9.237719 | 0.578547 | 2032 | ASR | 8.847779 | 9.62766 | 8.751161 | 9.724277 | 8.638345 | 9.837094 | 8.496022 | 9.979417 | 8.103767 | 10.37167 |
| 44 | 9.197687 | 0.60541 | 2033 | ASR | 8.78964 | 9.605734 | 8.688537 | 9.706837 | 8.570482 | 9.824892 | 8.421551 | 9.973823 | 8.011083 | 10.38429 |
| 45 | 9.157888 | 0.63219 | 2034 | ASR | 8.731792 | 9.583984 | 8.626217 | 9.68956 | 8.50294 | 9.812837 | 8.347421 | 9.968356 | 7.918796 | 10.39698 |
| 46 | 9.118684 | 0.65902 | 2035 | ASR | 8.674504 | 9.562863 | 8.564448 | 9.67292 | 8.435939 | 9.801429 | 8.27382 | 9.963548 | 7.827004 | 10.41036 |
| **Indonesia** | | | | | | | | | | | | | | |
| 1 | 4.714962 | 0.073767 | 1990 | ASR | 4.665243 | 4.764681 | 4.652924 | 4.777 | 4.63854 | 4.791384 | 4.620393 | 4.809531 | 4.570379 | 4.859545 |
| 2 | 4.784043 | 0.065294 | 1991 | ASR | 4.740035 | 4.828051 | 4.729131 | 4.838955 | 4.716399 | 4.851688 | 4.700337 | 4.86775 | 4.656068 | 4.912019 |
| 3 | 4.8531 | 0.063257 | 1992 | ASR | 4.810464 | 4.895735 | 4.7999 | 4.906299 | 4.787565 | 4.918634 | 4.772004 | 4.934196 | 4.729116 | 4.977084 |
| 4 | 4.922817 | 0.062589 | 1993 | ASR | 4.880632 | 4.965002 | 4.87018 | 4.975454 | 4.857975 | 4.987659 | 4.842578 | 5.003056 | 4.800143 | 5.045491 |
| 5 | 4.978883 | 0.062146 | 1994 | ASR | 4.936997 | 5.02077 | 4.926619 | 5.031148 | 4.9145 | 5.043267 | 4.899212 | 5.058555 | 4.857077 | 5.10069 |
| 6 | 5.024905 | 0.06176 | 1995 | ASR | 4.983279 | 5.066531 | 4.972965 | 5.076845 | 4.960922 | 5.088888 | 4.945729 | 5.104081 | 4.903856 | 5.145954 |
| 7 | 5.074707 | 0.061557 | 1996 | ASR | 5.033217 | 5.116197 | 5.022937 | 5.126477 | 5.010933 | 5.13848 | 4.99579 | 5.153624 | 4.954054 | 5.195359 |
| 8 | 5.123664 | 0.061299 | 1997 | ASR | 5.082348 | 5.16498 | 5.072112 | 5.175217 | 5.060158 | 5.18717 | 5.045079 | 5.20225 | 5.003518 | 5.24381 |
| 9 | 5.176897 | 0.061105 | 1998 | ASR | 5.135713 | 5.218082 | 5.125508 | 5.228287 | 5.113593 | 5.240202 | 5.098561 | 5.255234 | 5.057132 | 5.296663 |
| 10 | 5.237643 | 0.061011 | 1999 | ASR | 5.196522 | 5.278764 | 5.186333 | 5.288953 | 5.174436 | 5.30085 | 5.159427 | 5.315859 | 5.118062 | 5.357224 |
| 11 | 5.297882 | 0.060976 | 2000 | ASR | 5.256784 | 5.33898 | 5.246601 | 5.349163 | 5.234711 | 5.361053 | 5.21971 | 5.376053 | 5.178369 | 5.417395 |
| 12 | 5.347165 | 0.06095 | 2001 | ASR | 5.306085 | 5.388245 | 5.295907 | 5.398424 | 5.284021 | 5.410309 | 5.269028 | 5.425303 | 5.227704 | 5.466627 |
| 13 | 5.342531 | 0.060322 | 2002 | ASR | 5.301874 | 5.383188 | 5.2918 | 5.393262 | 5.280037 | 5.405024 | 5.265198 | 5.419864 | 5.224299 | 5.460762 |
| 14 | 5.33942 | 0.059809 | 2003 | ASR | 5.299109 | 5.379731 | 5.289121 | 5.389719 | 5.277459 | 5.401382 | 5.262746 | 5.416095 | 5.222195 | 5.456645 |
| 15 | 5.350222 | 0.059471 | 2004 | ASR | 5.310139 | 5.390305 | 5.300207 | 5.400237 | 5.28861 | 5.411834 | 5.273981 | 5.426464 | 5.233659 | 5.466785 |
| 16 | 5.363957 | 0.059246 | 2005 | ASR | 5.324025 | 5.403889 | 5.314131 | 5.413783 | 5.302578 | 5.425336 | 5.288003 | 5.43991 | 5.247834 | 5.480079 |
| 17 | 5.374939 | 0.05927 | 2006 | ASR | 5.334992 | 5.414887 | 5.325094 | 5.424785 | 5.313536 | 5.436343 | 5.298956 | 5.450923 | 5.258771 | 5.491108 |
| 18 | 5.326201 | 0.058925 | 2007 | ASR | 5.286485 | 5.365916 | 5.276645 | 5.375756 | 5.265155 | 5.387247 | 5.250659 | 5.401742 | 5.210708 | 5.441693 |
| 19 | 5.096216 | 0.056147 | 2008 | ASR | 5.058372 | 5.134059 | 5.048996 | 5.143436 | 5.038047 | 5.154385 | 5.024235 | 5.168197 | 4.986167 | 5.206265 |
| 20 | 4.904671 | 0.054571 | 2009 | ASR | 4.86789 | 4.941452 | 4.858777 | 4.950565 | 4.848135 | 4.961206 | 4.834711 | 4.974631 | 4.797712 | 5.01163 |
| 21 | 4.763385 | 0.053546 | 2010 | ASR | 4.727295 | 4.799475 | 4.718353 | 4.808417 | 4.707911 | 4.818858 | 4.694739 | 4.832031 | 4.658435 | 4.868335 |
| 22 | 4.685177 | 0.052951 | 2011 | ASR | 4.649488 | 4.720866 | 4.640645 | 4.729708 | 4.63032 | 4.740034 | 4.617294 | 4.75306 | 4.581393 | 4.78896 |
| 23 | 4.742776 | 0.052484 | 2012 | ASR | 4.707402 | 4.77815 | 4.698637 | 4.786915 | 4.688403 | 4.797149 | 4.675492 | 4.81006 | 4.639908 | 4.845644 |
| 24 | 4.792762 | 0.052468 | 2013 | ASR | 4.757399 | 4.828126 | 4.748637 | 4.836888 | 4.738406 | 4.847119 | 4.725499 | 4.860026 | 4.689926 | 4.895599 |
| 25 | 4.84598 | 0.052617 | 2014 | ASR | 4.810516 | 4.881444 | 4.801729 | 4.890231 | 4.791469 | 4.900491 | 4.778525 | 4.913435 | 4.742851 | 4.949109 |
| 26 | 4.847818 | 0.052309 | 2015 | ASR | 4.812561 | 4.883074 | 4.803826 | 4.89181 | 4.793626 | 4.90201 | 4.780758 | 4.914878 | 4.745292 | 4.950344 |
| 27 | 4.836333 | 0.051885 | 2016 | ASR | 4.801362 | 4.871304 | 4.792698 | 4.879969 | 4.78258 | 4.890087 | 4.769816 | 4.90285 | 4.734638 | 4.938029 |
| 28 | 4.823089 | 0.051467 | 2017 | ASR | 4.7884 | 4.857778 | 4.779805 | 4.866373 | 4.769769 | 4.876409 | 4.757108 | 4.88907 | 4.722213 | 4.923965 |
| 29 | 4.806688 | 0.051045 | 2018 | ASR | 4.772284 | 4.841092 | 4.763759 | 4.849616 | 4.753806 | 4.85957 | 4.741249 | 4.872127 | 4.70664 | 4.906736 |
| 30 | 4.793316 | 0.050699 | 2019 | ASR | 4.759145 | 4.827488 | 4.750679 | 4.835954 | 4.740792 | 4.845841 | 4.72832 | 4.858313 | 4.693946 | 4.892687 |
| 31 | 4.779099 | 0.050794 | 2020 | ASR | 4.744865 | 4.813334 | 4.736382 | 4.821817 | 4.726477 | 4.831721 | 4.713982 | 4.844217 | 4.679544 | 4.878655 |
| 32 | 4.745966 | 0.054532 | 2021 | ASR | 4.709212 | 4.782721 | 4.700105 | 4.791828 | 4.689471 | 4.802462 | 4.676056 | 4.815876 | 4.639083 | 4.852849 |
| 33 | 4.694864 | 0.110238 | 2022 | ASR | 4.620563 | 4.769164 | 4.602153 | 4.787574 | 4.580657 | 4.80907 | 4.553538 | 4.836189 | 4.478796 | 4.910931 |
| 34 | 4.647328 | 0.142137 | 2023 | ASR | 4.551528 | 4.743128 | 4.527791 | 4.766865 | 4.500075 | 4.794582 | 4.465109 | 4.829547 | 4.36874 | 4.925916 |
| 35 | 4.597578 | 0.168403 | 2024 | ASR | 4.484074 | 4.711081 | 4.455951 | 4.739205 | 4.423112 | 4.772043 | 4.381685 | 4.81347 | 4.267508 | 4.927648 |
| 36 | 4.545805 | 0.191263 | 2025 | ASR | 4.416893 | 4.674716 | 4.384953 | 4.706657 | 4.347656 | 4.743953 | 4.300606 | 4.791003 | 4.17093 | 4.920679 |
| 37 | 4.492311 | 0.211759 | 2026 | ASR | 4.349585 | 4.635036 | 4.314221 | 4.6704 | 4.272928 | 4.711693 | 4.220836 | 4.763786 | 4.077263 | 4.907358 |
| 38 | 4.437394 | 0.230475 | 2027 | ASR | 4.282053 | 4.592734 | 4.243564 | 4.631223 | 4.198621 | 4.676166 | 4.141924 | 4.732863 | 3.985662 | 4.889125 |
| 39 | 4.381195 | 0.24769 | 2028 | ASR | 4.214252 | 4.548138 | 4.172888 | 4.589502 | 4.124589 | 4.637802 | 4.063657 | 4.698733 | 3.895723 | 4.866667 |
| 40 | 4.324091 | 0.263628 | 2029 | ASR | 4.146406 | 4.501776 | 4.10238 | 4.545802 | 4.050973 | 4.59721 | 3.98612 | 4.662062 | 3.80738 | 4.840802 |
| 41 | 4.26632 | 0.278472 | 2030 | ASR | 4.07863 | 4.454011 | 4.032125 | 4.500516 | 3.977823 | 4.554818 | 3.909319 | 4.623322 | 3.720514 | 4.812126 |
| 42 | 4.208231 | 0.292387 | 2031 | ASR | 4.011162 | 4.405299 | 3.962333 | 4.454128 | 3.905318 | 4.511143 | 3.833391 | 4.583071 | 3.635152 | 4.781309 |
| 43 | 4.150177 | 0.30551 | 2032 | ASR | 3.944264 | 4.356091 | 3.893243 | 4.407111 | 3.833669 | 4.466685 | 3.758514 | 4.541841 | 3.551378 | 4.748976 |
| 44 | 4.092396 | 0.317892 | 2033 | ASR | 3.878138 | 4.306655 | 3.82505 | 4.359743 | 3.763061 | 4.421732 | 3.684859 | 4.499933 | 3.469329 | 4.715464 |
| 45 | 4.03525 | 0.32961 | 2034 | ASR | 3.813093 | 4.257408 | 3.758048 | 4.312453 | 3.693774 | 4.376727 | 3.61269 | 4.457811 | 3.389214 | 4.681287 |
| 46 | 3.978976 | 0.340749 | 2035 | ASR | 3.749311 | 4.208641 | 3.692406 | 4.265546 | 3.62596 | 4.331992 | 3.542135 | 4.415817 | 3.311107 | 4.646845 |
| **Lao People's Democratic Republic** | | | | | | | | | | | | | | |
| 1 | 3.647323 | 0.329052 | 1990 | ASR | 3.425542 | 3.869104 | 3.370591 | 3.924056 | 3.306426 | 3.988221 | 3.225479 | 4.069168 | 3.002382 | 4.292265 |
| 2 | 3.676675 | 0.302824 | 1991 | ASR | 3.472572 | 3.880779 | 3.422 | 3.93135 | 3.362949 | 3.990401 | 3.288454 | 4.064896 | 3.08314 | 4.270211 |
| 3 | 3.703397 | 0.278488 | 1992 | ASR | 3.515697 | 3.891098 | 3.469189 | 3.937606 | 3.414884 | 3.991911 | 3.346376 | 4.060419 | 3.157561 | 4.249234 |
| 4 | 3.729378 | 0.256366 | 1993 | ASR | 3.556587 | 3.902169 | 3.513774 | 3.944982 | 3.463783 | 3.994973 | 3.400717 | 4.058039 | 3.2269 | 4.231855 |
| 5 | 3.755012 | 0.236636 | 1994 | ASR | 3.595519 | 3.914504 | 3.556001 | 3.954022 | 3.509857 | 4.000166 | 3.451645 | 4.058378 | 3.291206 | 4.218817 |
| 6 | 3.78066 | 0.219298 | 1995 | ASR | 3.632853 | 3.928466 | 3.59623 | 3.965089 | 3.553467 | 4.007852 | 3.499519 | 4.0618 | 3.350835 | 4.210484 |
| 7 | 3.801585 | 0.203913 | 1996 | ASR | 3.664148 | 3.939022 | 3.630094 | 3.973075 | 3.590331 | 4.012838 | 3.540169 | 4.063001 | 3.401916 | 4.201254 |
| 8 | 3.821449 | 0.19064 | 1997 | ASR | 3.692958 | 3.949941 | 3.661121 | 3.981777 | 3.623947 | 4.018952 | 3.577049 | 4.065849 | 3.447796 | 4.195103 |
| 9 | 3.84118 | 0.179424 | 1998 | ASR | 3.720249 | 3.962112 | 3.690285 | 3.992076 | 3.655297 | 4.027063 | 3.611159 | 4.071202 | 3.48951 | 4.192851 |
| 10 | 3.856657 | 0.169869 | 1999 | ASR | 3.742165 | 3.971149 | 3.713797 | 3.999517 | 3.680672 | 4.032642 | 3.638885 | 4.07443 | 3.523713 | 4.189601 |
| 11 | 3.87357 | 0.162026 | 2000 | ASR | 3.764365 | 3.982776 | 3.737306 | 4.009834 | 3.705711 | 4.041429 | 3.665853 | 4.081288 | 3.555999 | 4.191141 |
| 12 | 3.884788 | 0.155468 | 2001 | ASR | 3.780003 | 3.989573 | 3.75404 | 4.015536 | 3.723723 | 4.045852 | 3.685478 | 4.084097 | 3.580071 | 4.189504 |
| 13 | 3.89688 | 0.150279 | 2002 | ASR | 3.795592 | 3.998168 | 3.770495 | 4.023265 | 3.74119 | 4.052569 | 3.704222 | 4.089538 | 3.602332 | 4.191428 |
| 14 | 3.904662 | 0.145836 | 2003 | ASR | 3.806369 | 4.002956 | 3.782014 | 4.02731 | 3.753576 | 4.055749 | 3.7177 | 4.091624 | 3.618823 | 4.190501 |
| 15 | 3.91144 | 0.142681 | 2004 | ASR | 3.815273 | 4.007607 | 3.791446 | 4.031435 | 3.763623 | 4.059258 | 3.728523 | 4.094357 | 3.631786 | 4.191095 |
| 16 | 3.914873 | 0.140154 | 2005 | ASR | 3.820409 | 4.009336 | 3.797003 | 4.032742 | 3.769673 | 4.060072 | 3.735195 | 4.09455 | 3.64017 | 4.189575 |
| 17 | 3.918256 | 0.138245 | 2006 | ASR | 3.82508 | 4.011433 | 3.801993 | 4.03452 | 3.775035 | 4.061478 | 3.741027 | 4.095486 | 3.647297 | 4.189216 |
| 18 | 3.918046 | 0.136899 | 2007 | ASR | 3.825776 | 4.010316 | 3.802914 | 4.033178 | 3.776219 | 4.059874 | 3.742541 | 4.093551 | 3.649724 | 4.186369 |
| 19 | 3.915676 | 0.136139 | 2008 | ASR | 3.823918 | 4.007434 | 3.801182 | 4.030169 | 3.774635 | 4.056716 | 3.741145 | 4.090206 | 3.648842 | 4.182509 |
| 20 | 3.913172 | 0.135925 | 2009 | ASR | 3.821558 | 4.004786 | 3.798859 | 4.027485 | 3.772354 | 4.053991 | 3.738916 | 4.087429 | 3.646758 | 4.179586 |
| 21 | 3.910415 | 0.13699 | 2010 | ASR | 3.818085 | 4.002746 | 3.795207 | 4.025624 | 3.768494 | 4.052337 | 3.734795 | 4.086036 | 3.641916 | 4.178915 |
| 22 | 3.905332 | 0.138819 | 2011 | ASR | 3.811768 | 3.998896 | 3.788585 | 4.022079 | 3.761515 | 4.049148 | 3.727366 | 4.083298 | 3.633247 | 4.177417 |
| 23 | 3.898504 | 0.140557 | 2012 | ASR | 3.803769 | 3.993239 | 3.780296 | 4.016712 | 3.752887 | 4.044121 | 3.71831 | 4.078698 | 3.623013 | 4.173995 |
| 24 | 3.891909 | 0.142627 | 2013 | ASR | 3.795779 | 3.98804 | 3.77196 | 4.011858 | 3.744148 | 4.039671 | 3.709061 | 4.074757 | 3.61236 | 4.171458 |
| 25 | 3.881873 | 0.145069 | 2014 | ASR | 3.784097 | 3.97965 | 3.75987 | 4.003876 | 3.731582 | 4.032165 | 3.695895 | 4.067852 | 3.597538 | 4.166208 |
| 26 | 3.87195 | 0.148009 | 2015 | ASR | 3.772192 | 3.971708 | 3.747474 | 3.996425 | 3.718613 | 4.025287 | 3.682202 | 4.061697 | 3.581852 | 4.162047 |
| 27 | 3.858751 | 0.151353 | 2016 | ASR | 3.756739 | 3.960763 | 3.731463 | 3.986039 | 3.701949 | 4.015553 | 3.664716 | 4.052786 | 3.562099 | 4.155403 |
| 28 | 3.840107 | 0.153921 | 2017 | ASR | 3.736364 | 3.94385 | 3.710659 | 3.969555 | 3.680645 | 3.999569 | 3.64278 | 4.037434 | 3.538422 | 4.141793 |
| 29 | 3.824029 | 0.159658 | 2018 | ASR | 3.716419 | 3.931638 | 3.689756 | 3.958301 | 3.658623 | 3.989434 | 3.619347 | 4.02871 | 3.511099 | 4.136959 |
| 30 | 3.808361 | 0.166663 | 2019 | ASR | 3.69603 | 3.920692 | 3.668197 | 3.948525 | 3.635698 | 3.981025 | 3.594699 | 4.022024 | 3.481701 | 4.135022 |
| 31 | 3.787341 | 0.174611 | 2020 | ASR | 3.669653 | 3.905029 | 3.640493 | 3.934189 | 3.606444 | 3.968238 | 3.563489 | 4.011192 | 3.445103 | 4.129579 |
| 32 | 3.767805 | 0.184571 | 2021 | ASR | 3.643405 | 3.892206 | 3.612581 | 3.923029 | 3.57659 | 3.959021 | 3.531186 | 4.004425 | 3.406047 | 4.129564 |
| 33 | 3.745339 | 0.202588 | 2022 | ASR | 3.608794 | 3.881883 | 3.574962 | 3.915715 | 3.535457 | 3.95522 | 3.48562 | 4.005057 | 3.348265 | 4.142412 |
| 34 | 3.722256 | 0.215518 | 2023 | ASR | 3.576997 | 3.867515 | 3.541005 | 3.903506 | 3.498979 | 3.945532 | 3.445962 | 3.998549 | 3.299841 | 4.14467 |
| 35 | 3.698069 | 0.228801 | 2024 | ASR | 3.543857 | 3.852281 | 3.505647 | 3.890491 | 3.461031 | 3.935107 | 3.404746 | 3.991392 | 3.249618 | 4.14652 |
| 36 | 3.672821 | 0.24239 | 2025 | ASR | 3.50945 | 3.836192 | 3.468971 | 3.876671 | 3.421705 | 3.923937 | 3.362077 | 3.983565 | 3.197737 | 4.147905 |
| 37 | 3.646536 | 0.256192 | 2026 | ASR | 3.473863 | 3.81921 | 3.431079 | 3.861994 | 3.381121 | 3.911952 | 3.318098 | 3.974975 | 3.1444 | 4.148673 |
| 38 | 3.619262 | 0.270095 | 2027 | ASR | 3.437219 | 3.801306 | 3.392113 | 3.846412 | 3.339444 | 3.89908 | 3.273001 | 3.965524 | 3.089877 | 4.148648 |
| 39 | 3.589397 | 0.281536 | 2028 | ASR | 3.399642 | 3.779152 | 3.352626 | 3.826169 | 3.297727 | 3.881068 | 3.228469 | 3.950326 | 3.037588 | 4.141207 |
| 40 | 3.559595 | 0.294358 | 2029 | ASR | 3.361197 | 3.757992 | 3.312039 | 3.80715 | 3.254639 | 3.86455 | 3.182227 | 3.936962 | 2.982652 | 4.136537 |
| 41 | 3.529423 | 0.307795 | 2030 | ASR | 3.321969 | 3.736877 | 3.270567 | 3.788279 | 3.210547 | 3.848299 | 3.13483 | 3.924017 | 2.926145 | 4.132702 |
| 42 | 3.498634 | 0.321351 | 2031 | ASR | 3.282044 | 3.715224 | 3.228378 | 3.76889 | 3.165715 | 3.831553 | 3.086663 | 3.910605 | 2.868787 | 4.128481 |
| 43 | 3.467288 | 0.334962 | 2032 | ASR | 3.241523 | 3.693052 | 3.185585 | 3.748991 | 3.120267 | 3.814309 | 3.037866 | 3.89671 | 2.810762 | 4.123814 |
| 44 | 3.435466 | 0.348608 | 2033 | ASR | 3.200504 | 3.670428 | 3.142286 | 3.728646 | 3.074308 | 3.796624 | 2.98855 | 3.882382 | 2.752194 | 4.118739 |
| 45 | 3.4032 | 0.36218 | 2034 | ASR | 3.159091 | 3.647309 | 3.098607 | 3.707793 | 3.027982 | 3.778418 | 2.938886 | 3.867515 | 2.693328 | 4.113072 |
| 46 | 3.370539 | 0.375692 | 2035 | ASR | 3.117322 | 3.623755 | 3.054581 | 3.686496 | 2.981321 | 3.759756 | 2.888901 | 3.852176 | 2.634181 | 4.106896 |
| **Malaysia** | | | | | | | | | | | | | | |
| 1 | 2.484823 | 0.07638 | 1990 | ASR | 2.433342 | 2.536303 | 2.420587 | 2.549058 | 2.405693 | 2.563953 | 2.386903 | 2.582742 | 2.335117 | 2.634528 |
| 2 | 2.519441 | 0.071813 | 1991 | ASR | 2.471039 | 2.567843 | 2.459046 | 2.579836 | 2.445042 | 2.593839 | 2.427376 | 2.611505 | 2.378687 | 2.660195 |
| 3 | 2.553475 | 0.068156 | 1992 | ASR | 2.507538 | 2.599411 | 2.496156 | 2.610793 | 2.482865 | 2.624084 | 2.466099 | 2.64085 | 2.41989 | 2.68706 |
| 4 | 2.586148 | 0.065253 | 1993 | ASR | 2.542167 | 2.630129 | 2.53127 | 2.641026 | 2.518545 | 2.65375 | 2.502493 | 2.669803 | 2.458251 | 2.714044 |
| 5 | 2.61157 | 0.062809 | 1994 | ASR | 2.569237 | 2.653904 | 2.558748 | 2.664393 | 2.5465 | 2.676641 | 2.531049 | 2.692092 | 2.488464 | 2.734676 |
| 6 | 2.638928 | 0.060947 | 1995 | ASR | 2.597849 | 2.680006 | 2.587671 | 2.690185 | 2.575787 | 2.702069 | 2.560793 | 2.717062 | 2.519471 | 2.758385 |
| 7 | 2.666536 | 0.059515 | 1996 | ASR | 2.626423 | 2.706649 | 2.616484 | 2.716588 | 2.604879 | 2.728194 | 2.590238 | 2.742835 | 2.549887 | 2.783186 |
| 8 | 2.692819 | 0.058431 | 1997 | ASR | 2.653436 | 2.732201 | 2.643678 | 2.741959 | 2.632284 | 2.753353 | 2.61791 | 2.767727 | 2.578294 | 2.807343 |
| 9 | 2.718415 | 0.057657 | 1998 | ASR | 2.679554 | 2.757276 | 2.669925 | 2.766905 | 2.658682 | 2.778148 | 2.644499 | 2.792332 | 2.605407 | 2.831423 |
| 10 | 2.73935 | 0.057019 | 1999 | ASR | 2.700919 | 2.777781 | 2.691397 | 2.787303 | 2.680279 | 2.798422 | 2.666252 | 2.812448 | 2.627593 | 2.851107 |
| 11 | 2.748505 | 0.056288 | 2000 | ASR | 2.710567 | 2.786443 | 2.701167 | 2.795843 | 2.690191 | 2.806819 | 2.676344 | 2.820666 | 2.638181 | 2.858829 |
| 12 | 2.752204 | 0.055616 | 2001 | ASR | 2.714719 | 2.789689 | 2.705431 | 2.798977 | 2.694586 | 2.809822 | 2.680904 | 2.823503 | 2.643197 | 2.861211 |
| 13 | 2.755373 | 0.055109 | 2002 | ASR | 2.71823 | 2.792516 | 2.709027 | 2.801719 | 2.69828 | 2.812466 | 2.684724 | 2.826022 | 2.64736 | 2.863386 |
| 14 | 2.762127 | 0.054799 | 2003 | ASR | 2.725193 | 2.799062 | 2.716041 | 2.808213 | 2.705356 | 2.818899 | 2.691875 | 2.832379 | 2.654722 | 2.869533 |
| 15 | 2.761671 | 0.05444 | 2004 | ASR | 2.724978 | 2.798363 | 2.715887 | 2.807455 | 2.705271 | 2.818071 | 2.691879 | 2.831463 | 2.654968 | 2.868373 |
| 16 | 2.757035 | 0.054021 | 2005 | ASR | 2.720624 | 2.793445 | 2.711603 | 2.802467 | 2.701069 | 2.813001 | 2.687779 | 2.82629 | 2.651153 | 2.862916 |
| 17 | 2.750027 | 0.053559 | 2006 | ASR | 2.713928 | 2.786126 | 2.704984 | 2.79507 | 2.69454 | 2.805514 | 2.681364 | 2.818689 | 2.645051 | 2.855002 |
| 18 | 2.741621 | 0.053152 | 2007 | ASR | 2.705797 | 2.777446 | 2.696921 | 2.786322 | 2.686556 | 2.796687 | 2.673481 | 2.809762 | 2.637444 | 2.845799 |
| 19 | 2.73431 | 0.052827 | 2008 | ASR | 2.698705 | 2.769916 | 2.689883 | 2.778738 | 2.679581 | 2.789039 | 2.666586 | 2.802035 | 2.630769 | 2.837852 |
| 20 | 2.725273 | 0.052534 | 2009 | ASR | 2.689865 | 2.760681 | 2.681091 | 2.769454 | 2.670847 | 2.779698 | 2.657924 | 2.792621 | 2.622306 | 2.82824 |
| 21 | 2.711368 | 0.052155 | 2010 | ASR | 2.676216 | 2.746521 | 2.667506 | 2.755231 | 2.657336 | 2.765401 | 2.644506 | 2.778231 | 2.609145 | 2.813592 |
| 22 | 2.687375 | 0.051555 | 2011 | ASR | 2.652627 | 2.722123 | 2.644017 | 2.730733 | 2.633964 | 2.740786 | 2.621281 | 2.753469 | 2.586326 | 2.788424 |
| 23 | 2.662615 | 0.051071 | 2012 | ASR | 2.628193 | 2.697037 | 2.619664 | 2.705566 | 2.609705 | 2.715525 | 2.597142 | 2.728089 | 2.562515 | 2.762715 |
| 24 | 2.636334 | 0.050658 | 2013 | ASR | 2.602191 | 2.670477 | 2.593731 | 2.678937 | 2.583853 | 2.688815 | 2.571391 | 2.701277 | 2.537045 | 2.735623 |
| 25 | 2.610088 | 0.050314 | 2014 | ASR | 2.576177 | 2.644 | 2.567774 | 2.652402 | 2.557963 | 2.662214 | 2.545586 | 2.674591 | 2.511473 | 2.708704 |
| 26 | 2.585083 | 0.050123 | 2015 | ASR | 2.551301 | 2.618866 | 2.54293 | 2.627237 | 2.533156 | 2.637011 | 2.520826 | 2.649341 | 2.486843 | 2.683324 |
| 27 | 2.555654 | 0.050025 | 2016 | ASR | 2.521937 | 2.589371 | 2.513583 | 2.597725 | 2.503828 | 2.60748 | 2.491522 | 2.619787 | 2.457604 | 2.653704 |
| 28 | 2.522802 | 0.050104 | 2017 | ASR | 2.489032 | 2.556573 | 2.480665 | 2.56494 | 2.470894 | 2.57471 | 2.458569 | 2.587036 | 2.424598 | 2.621007 |
| 29 | 2.486398 | 0.050434 | 2018 | ASR | 2.452405 | 2.520391 | 2.443983 | 2.528813 | 2.434148 | 2.538648 | 2.421741 | 2.551055 | 2.387547 | 2.585249 |
| 30 | 2.448809 | 0.051217 | 2019 | ASR | 2.414289 | 2.483329 | 2.405736 | 2.491883 | 2.395748 | 2.50187 | 2.383149 | 2.514469 | 2.348424 | 2.549195 |
| 31 | 2.392273 | 0.052338 | 2020 | ASR | 2.356997 | 2.427549 | 2.348257 | 2.436289 | 2.338051 | 2.446495 | 2.325176 | 2.45937 | 2.289691 | 2.494855 |
| 32 | 2.383346 | 0.055163 | 2021 | ASR | 2.346167 | 2.420526 | 2.336954 | 2.429738 | 2.326198 | 2.440495 | 2.312628 | 2.454065 | 2.275228 | 2.491465 |
| 33 | 2.345423 | 0.062432 | 2022 | ASR | 2.303344 | 2.387502 | 2.292918 | 2.397928 | 2.280744 | 2.410102 | 2.265386 | 2.425461 | 2.223057 | 2.467789 |
| 34 | 2.312803 | 0.066432 | 2023 | ASR | 2.268028 | 2.357578 | 2.256934 | 2.368672 | 2.24398 | 2.381627 | 2.227638 | 2.397969 | 2.182597 | 2.44301 |
| 35 | 2.280624 | 0.070337 | 2024 | ASR | 2.233217 | 2.328032 | 2.221471 | 2.339778 | 2.207755 | 2.353494 | 2.190452 | 2.370797 | 2.142763 | 2.418486 |
| 36 | 2.249095 | 0.074188 | 2025 | ASR | 2.199092 | 2.299097 | 2.186702 | 2.311487 | 2.172236 | 2.325954 | 2.153985 | 2.344204 | 2.103686 | 2.394503 |
| 37 | 2.218449 | 0.078023 | 2026 | ASR | 2.165861 | 2.271036 | 2.152831 | 2.284066 | 2.137617 | 2.299281 | 2.118423 | 2.318474 | 2.065523 | 2.371374 |
| 38 | 2.188464 | 0.081853 | 2027 | ASR | 2.133295 | 2.243632 | 2.119626 | 2.257302 | 2.103665 | 2.273263 | 2.083529 | 2.293399 | 2.028033 | 2.348895 |
| 39 | 2.159175 | 0.085665 | 2028 | ASR | 2.101437 | 2.216913 | 2.087131 | 2.231219 | 2.070426 | 2.247924 | 2.049352 | 2.268998 | 1.991272 | 2.327079 |
| 40 | 2.130655 | 0.089485 | 2029 | ASR | 2.070342 | 2.190968 | 2.055398 | 2.205912 | 2.037949 | 2.223362 | 2.015935 | 2.245375 | 1.955264 | 2.306046 |
| 41 | 2.103039 | 0.093355 | 2030 | ASR | 2.040118 | 2.165961 | 2.024528 | 2.181551 | 2.006324 | 2.199755 | 1.983358 | 2.222721 | 1.920063 | 2.286015 |
| 42 | 2.076527 | 0.097318 | 2031 | ASR | 2.010934 | 2.142119 | 1.994682 | 2.158372 | 1.975705 | 2.177349 | 1.951765 | 2.201289 | 1.885783 | 2.267271 |
| 43 | 2.050973 | 0.101395 | 2032 | ASR | 1.982633 | 2.119313 | 1.9657 | 2.136246 | 1.945928 | 2.156018 | 1.920985 | 2.180961 | 1.85224 | 2.249706 |
| 44 | 2.026417 | 0.105586 | 2033 | ASR | 1.955252 | 2.097582 | 1.937619 | 2.115215 | 1.91703 | 2.135804 | 1.891056 | 2.161778 | 1.819469 | 2.233365 |
| 45 | 2.002896 | 0.109922 | 2034 | ASR | 1.928808 | 2.076983 | 1.910451 | 2.09534 | 1.889016 | 2.116775 | 1.861976 | 2.143816 | 1.787448 | 2.218343 |
| 46 | 1.980474 | 0.114449 | 2035 | ASR | 1.903336 | 2.057613 | 1.884223 | 2.076726 | 1.861905 | 2.099043 | 1.833751 | 2.127198 | 1.756154 | 2.204794 |
| **Myanmar** | | | | | | | | | | | | | | |
| 1 | 6.156046 | 0.134748 | 1990 | ASR | 6.065226 | 6.246866 | 6.042723 | 6.269369 | 6.016447 | 6.295645 | 5.983299 | 6.328793 | 5.89194 | 6.420152 |
| 2 | 6.164203 | 0.119663 | 1991 | ASR | 6.083551 | 6.244856 | 6.063567 | 6.26484 | 6.040233 | 6.288174 | 6.010796 | 6.317611 | 5.929664 | 6.398742 |
| 3 | 6.17412 | 0.109534 | 1992 | ASR | 6.100294 | 6.247945 | 6.082002 | 6.266237 | 6.060643 | 6.287597 | 6.033698 | 6.314542 | 5.959434 | 6.388806 |
| 4 | 6.183805 | 0.102702 | 1993 | ASR | 6.114584 | 6.253026 | 6.097433 | 6.270177 | 6.077407 | 6.290204 | 6.052142 | 6.315469 | 5.98251 | 6.385101 |
| 5 | 6.192119 | 0.098048 | 1994 | ASR | 6.126034 | 6.258204 | 6.10966 | 6.274578 | 6.090541 | 6.293697 | 6.066421 | 6.317817 | 5.999944 | 6.384294 |
| 6 | 6.198324 | 0.094846 | 1995 | ASR | 6.134398 | 6.26225 | 6.118558 | 6.278089 | 6.100063 | 6.296584 | 6.076731 | 6.319916 | 6.012426 | 6.384222 |
| 7 | 6.202268 | 0.092613 | 1996 | ASR | 6.139847 | 6.264689 | 6.12438 | 6.280156 | 6.106321 | 6.298215 | 6.083538 | 6.320998 | 6.020746 | 6.38379 |
| 8 | 6.202729 | 0.091001 | 1997 | ASR | 6.141394 | 6.264063 | 6.126197 | 6.27926 | 6.108452 | 6.297006 | 6.086066 | 6.319392 | 6.024367 | 6.38109 |
| 9 | 6.199475 | 0.08978 | 1998 | ASR | 6.138963 | 6.259986 | 6.12397 | 6.27498 | 6.106463 | 6.292487 | 6.084377 | 6.314572 | 6.023506 | 6.375443 |
| 10 | 6.19062 | 0.088753 | 1999 | ASR | 6.130801 | 6.250439 | 6.115979 | 6.265261 | 6.098672 | 6.282567 | 6.076839 | 6.304401 | 6.016665 | 6.364575 |
| 11 | 6.174934 | 0.087798 | 2000 | ASR | 6.115758 | 6.234109 | 6.101096 | 6.248771 | 6.083975 | 6.265892 | 6.062377 | 6.28749 | 6.002851 | 6.347017 |
| 12 | 6.153034 | 0.086848 | 2001 | ASR | 6.094498 | 6.211569 | 6.079994 | 6.226073 | 6.063059 | 6.243008 | 6.041694 | 6.264373 | 5.982811 | 6.323256 |
| 13 | 6.124691 | 0.085851 | 2002 | ASR | 6.066828 | 6.182555 | 6.052491 | 6.196892 | 6.03575 | 6.213633 | 6.014631 | 6.234752 | 5.956424 | 6.292959 |
| 14 | 6.086824 | 0.084679 | 2003 | ASR | 6.02975 | 6.143898 | 6.015609 | 6.158039 | 5.999096 | 6.174552 | 5.978265 | 6.195383 | 5.920852 | 6.252796 |
| 15 | 6.038589 | 0.083316 | 2004 | ASR | 5.982434 | 6.094744 | 5.96852 | 6.108658 | 5.952274 | 6.124905 | 5.931778 | 6.145401 | 5.875289 | 6.201889 |
| 16 | 5.979933 | 0.08181 | 2005 | ASR | 5.924793 | 6.035073 | 5.911131 | 6.048735 | 5.895178 | 6.064688 | 5.875053 | 6.084813 | 5.819586 | 6.14028 |
| 17 | 5.916551 | 0.080331 | 2006 | ASR | 5.862408 | 5.970694 | 5.848993 | 5.98411 | 5.833328 | 5.999774 | 5.813567 | 6.019536 | 5.759102 | 6.074 |
| 18 | 5.847305 | 0.078923 | 2007 | ASR | 5.794111 | 5.900499 | 5.78093 | 5.913679 | 5.76554 | 5.929069 | 5.746125 | 5.948484 | 5.692616 | 6.001994 |
| 19 | 5.777576 | 0.077746 | 2008 | ASR | 5.725175 | 5.829977 | 5.712191 | 5.842961 | 5.697031 | 5.858121 | 5.677905 | 5.877247 | 5.625193 | 5.929959 |
| 20 | 5.708642 | 0.076696 | 2009 | ASR | 5.656949 | 5.760336 | 5.64414 | 5.773144 | 5.629185 | 5.7881 | 5.610317 | 5.806967 | 5.558317 | 5.858967 |
| 21 | 5.641843 | 0.075661 | 2010 | ASR | 5.590848 | 5.692839 | 5.578212 | 5.705474 | 5.563458 | 5.720228 | 5.544846 | 5.738841 | 5.493547 | 5.790139 |
| 22 | 5.584936 | 0.074661 | 2011 | ASR | 5.534615 | 5.635258 | 5.522147 | 5.647726 | 5.507588 | 5.662285 | 5.489221 | 5.680652 | 5.438601 | 5.731272 |
| 23 | 5.525931 | 0.073662 | 2012 | ASR | 5.476283 | 5.575579 | 5.463982 | 5.58788 | 5.449618 | 5.602244 | 5.431497 | 5.620365 | 5.381554 | 5.670308 |
| 24 | 5.480164 | 0.072613 | 2013 | ASR | 5.431222 | 5.529105 | 5.419096 | 5.541232 | 5.404936 | 5.555391 | 5.387073 | 5.573254 | 5.337841 | 5.622486 |
| 25 | 5.440099 | 0.07154 | 2014 | ASR | 5.391882 | 5.488317 | 5.379934 | 5.500265 | 5.365984 | 5.514215 | 5.348385 | 5.531814 | 5.299881 | 5.580318 |
| 26 | 5.407127 | 0.070599 | 2015 | ASR | 5.359543 | 5.454711 | 5.347753 | 5.466501 | 5.333987 | 5.480268 | 5.316619 | 5.497635 | 5.268753 | 5.545502 |
| 27 | 5.376242 | 0.069854 | 2016 | ASR | 5.32916 | 5.423323 | 5.317495 | 5.434989 | 5.303873 | 5.44861 | 5.286689 | 5.465794 | 5.239328 | 5.513155 |
| 28 | 5.348678 | 0.069469 | 2017 | ASR | 5.301856 | 5.395499 | 5.290254 | 5.407101 | 5.276708 | 5.420647 | 5.259619 | 5.437736 | 5.212519 | 5.484836 |
| 29 | 5.322871 | 0.069676 | 2018 | ASR | 5.275909 | 5.369833 | 5.264273 | 5.381469 | 5.250687 | 5.395056 | 5.233546 | 5.412196 | 5.186306 | 5.459437 |
| 30 | 5.298117 | 0.070933 | 2019 | ASR | 5.250308 | 5.345925 | 5.238462 | 5.357771 | 5.22463 | 5.371603 | 5.207181 | 5.389052 | 5.159089 | 5.437145 |
| 31 | 5.271946 | 0.074219 | 2020 | ASR | 5.221922 | 5.32197 | 5.209528 | 5.334365 | 5.195055 | 5.348837 | 5.176797 | 5.367095 | 5.126476 | 5.417416 |
| 32 | 5.235073 | 0.081204 | 2021 | ASR | 5.180341 | 5.289804 | 5.16678 | 5.303366 | 5.150945 | 5.3192 | 5.130969 | 5.339177 | 5.075913 | 5.394233 |
| 33 | 5.157043 | 0.104934 | 2022 | ASR | 5.086317 | 5.227769 | 5.068793 | 5.245293 | 5.048331 | 5.265755 | 5.022517 | 5.291569 | 4.951372 | 5.362714 |
| 34 | 5.095403 | 0.118721 | 2023 | ASR | 5.015385 | 5.17542 | 4.995559 | 5.195247 | 4.972408 | 5.218397 | 4.943203 | 5.247602 | 4.86271 | 5.328095 |
| 35 | 5.032852 | 0.131191 | 2024 | ASR | 4.944429 | 5.121275 | 4.92252 | 5.143184 | 4.896938 | 5.168766 | 4.864665 | 5.201039 | 4.775718 | 5.289986 |
| 36 | 4.969473 | 0.142674 | 2025 | ASR | 4.873311 | 5.065635 | 4.849485 | 5.089462 | 4.821663 | 5.117283 | 4.786566 | 5.152381 | 4.689833 | 5.249113 |
| 37 | 4.905346 | 0.153386 | 2026 | ASR | 4.801964 | 5.008728 | 4.776348 | 5.034343 | 4.746438 | 5.064253 | 4.708705 | 5.101986 | 4.60471 | 5.205982 |
| 38 | 4.840582 | 0.16346 | 2027 | ASR | 4.73041 | 4.950754 | 4.703112 | 4.978052 | 4.671237 | 5.009927 | 4.631026 | 5.050138 | 4.5202 | 5.160964 |
| 39 | 4.775553 | 0.172943 | 2028 | ASR | 4.65899 | 4.892116 | 4.630108 | 4.920998 | 4.596385 | 4.954722 | 4.553841 | 4.997266 | 4.436586 | 5.114521 |
| 40 | 4.710467 | 0.181903 | 2029 | ASR | 4.587864 | 4.833069 | 4.557486 | 4.863447 | 4.522015 | 4.898918 | 4.477266 | 4.943667 | 4.353936 | 5.066997 |
| 41 | 4.645436 | 0.190424 | 2030 | ASR | 4.517091 | 4.773782 | 4.48529 | 4.805583 | 4.448157 | 4.842715 | 4.401313 | 4.88956 | 4.272206 | 5.018667 |
| 42 | 4.580577 | 0.198579 | 2031 | ASR | 4.446735 | 4.714419 | 4.413572 | 4.747582 | 4.374849 | 4.786304 | 4.325999 | 4.835155 | 4.191363 | 4.969791 |
| 43 | 4.516032 | 0.206421 | 2032 | ASR | 4.376903 | 4.65516 | 4.342431 | 4.689632 | 4.302179 | 4.729884 | 4.251399 | 4.780664 | 4.111446 | 4.920618 |
| 44 | 4.45211 | 0.213961 | 2033 | ASR | 4.3079 | 4.59632 | 4.272169 | 4.632052 | 4.230446 | 4.673774 | 4.177812 | 4.726409 | 4.032746 | 4.871475 |
| 45 | 4.388993 | 0.221237 | 2034 | ASR | 4.239879 | 4.538108 | 4.202933 | 4.575054 | 4.159791 | 4.618195 | 4.105367 | 4.67262 | 3.955368 | 4.822619 |
| 46 | 4.326755 | 0.228303 | 2035 | ASR | 4.172879 | 4.480631 | 4.134752 | 4.518758 | 4.090233 | 4.563277 | 4.034071 | 4.61944 | 3.879281 | 4.774229 |
| **Philippines** | | | | | | | | | | | | | | |
| 1 | 2.169503 | 0.065563 | 1990 | ASR | 2.125313 | 2.213693 | 2.114364 | 2.224642 | 2.10158 | 2.237427 | 2.085451 | 2.253555 | 2.040999 | 2.298007 |
| 2 | 2.166473 | 0.057551 | 1991 | ASR | 2.127683 | 2.205262 | 2.118072 | 2.214873 | 2.106849 | 2.226096 | 2.092692 | 2.240253 | 2.053672 | 2.279273 |
| 3 | 2.167501 | 0.051242 | 1992 | ASR | 2.132964 | 2.202038 | 2.124407 | 2.210596 | 2.114415 | 2.220588 | 2.101809 | 2.233193 | 2.067068 | 2.267935 |
| 4 | 2.168832 | 0.046312 | 1993 | ASR | 2.137618 | 2.200046 | 2.129884 | 2.20778 | 2.120853 | 2.216811 | 2.109461 | 2.228204 | 2.078062 | 2.259603 |
| 5 | 2.171211 | 0.042571 | 1994 | ASR | 2.142518 | 2.199903 | 2.135409 | 2.207013 | 2.127107 | 2.215314 | 2.116635 | 2.225786 | 2.087772 | 2.254649 |
| 6 | 2.174362 | 0.03982 | 1995 | ASR | 2.147524 | 2.2012 | 2.140874 | 2.20785 | 2.133109 | 2.215615 | 2.123313 | 2.225411 | 2.096316 | 2.252409 |
| 7 | 2.177189 | 0.037846 | 1996 | ASR | 2.151681 | 2.202697 | 2.14536 | 2.209018 | 2.13798 | 2.216398 | 2.12867 | 2.225708 | 2.10301 | 2.251368 |
| 8 | 2.177689 | 0.036431 | 1997 | ASR | 2.153135 | 2.202243 | 2.147051 | 2.208327 | 2.139947 | 2.215431 | 2.130985 | 2.224393 | 2.106284 | 2.249094 |
| 9 | 2.18058 | 0.035491 | 1998 | ASR | 2.156659 | 2.2045 | 2.150732 | 2.210427 | 2.143811 | 2.217348 | 2.135081 | 2.226079 | 2.111018 | 2.250141 |
| 10 | 2.184367 | 0.03486 | 1999 | ASR | 2.160871 | 2.207863 | 2.15505 | 2.213684 | 2.148252 | 2.220482 | 2.139677 | 2.229057 | 2.116042 | 2.252692 |
| 11 | 2.189757 | 0.034431 | 2000 | ASR | 2.16655 | 2.212963 | 2.1608 | 2.218713 | 2.154086 | 2.225427 | 2.145616 | 2.233897 | 2.122272 | 2.257242 |
| 12 | 2.196971 | 0.034139 | 2001 | ASR | 2.173961 | 2.219981 | 2.16826 | 2.225682 | 2.161603 | 2.232339 | 2.153204 | 2.240738 | 2.130058 | 2.263884 |
| 13 | 2.205243 | 0.033941 | 2002 | ASR | 2.182367 | 2.228119 | 2.176699 | 2.233787 | 2.170081 | 2.240406 | 2.161731 | 2.248755 | 2.13872 | 2.271767 |
| 14 | 2.210935 | 0.033748 | 2003 | ASR | 2.188189 | 2.233681 | 2.182553 | 2.239317 | 2.175972 | 2.245898 | 2.16767 | 2.2542 | 2.144789 | 2.277081 |
| 15 | 2.218913 | 0.033579 | 2004 | ASR | 2.196281 | 2.241545 | 2.190674 | 2.247153 | 2.184126 | 2.253701 | 2.175865 | 2.261961 | 2.153099 | 2.284728 |
| 16 | 2.231779 | 0.03344 | 2005 | ASR | 2.20924 | 2.254318 | 2.203656 | 2.259902 | 2.197135 | 2.266423 | 2.188908 | 2.27465 | 2.166236 | 2.297322 |
| 17 | 2.244894 | 0.033299 | 2006 | ASR | 2.222451 | 2.267337 | 2.21689 | 2.272898 | 2.210396 | 2.279392 | 2.202205 | 2.287583 | 2.179628 | 2.31016 |
| 18 | 2.258256 | 0.033182 | 2007 | ASR | 2.235892 | 2.280621 | 2.23035 | 2.286162 | 2.22388 | 2.292632 | 2.215717 | 2.300795 | 2.19322 | 2.323292 |
| 19 | 2.274544 | 0.033114 | 2008 | ASR | 2.252225 | 2.296863 | 2.246695 | 2.302393 | 2.240238 | 2.308851 | 2.232092 | 2.316997 | 2.20964 | 2.339448 |
| 20 | 2.28925 | 0.033057 | 2009 | ASR | 2.266969 | 2.311531 | 2.261449 | 2.317051 | 2.255003 | 2.323497 | 2.246871 | 2.331629 | 2.224458 | 2.354042 |
| 21 | 2.303769 | 0.033019 | 2010 | ASR | 2.281514 | 2.326024 | 2.276 | 2.331538 | 2.269561 | 2.337976 | 2.261439 | 2.346099 | 2.239052 | 2.368486 |
| 22 | 2.317075 | 0.032978 | 2011 | ASR | 2.294848 | 2.339302 | 2.28934 | 2.344809 | 2.28291 | 2.35124 | 2.274797 | 2.359352 | 2.252438 | 2.381711 |
| 23 | 2.331589 | 0.032978 | 2012 | ASR | 2.309362 | 2.353816 | 2.303854 | 2.359323 | 2.297424 | 2.365753 | 2.289311 | 2.373866 | 2.266952 | 2.396225 |
| 24 | 2.344616 | 0.03299 | 2013 | ASR | 2.322381 | 2.366851 | 2.316872 | 2.372361 | 2.310439 | 2.378794 | 2.302323 | 2.386909 | 2.279956 | 2.409276 |
| 25 | 2.356486 | 0.033022 | 2014 | ASR | 2.33423 | 2.378743 | 2.328715 | 2.384257 | 2.322276 | 2.390697 | 2.314152 | 2.39882 | 2.291764 | 2.421208 |
| 26 | 2.371173 | 0.03316 | 2015 | ASR | 2.348824 | 2.393523 | 2.343286 | 2.399061 | 2.33682 | 2.405527 | 2.328662 | 2.413685 | 2.30618 | 2.436167 |
| 27 | 2.384586 | 0.033383 | 2016 | ASR | 2.362085 | 2.407086 | 2.35651 | 2.412661 | 2.350001 | 2.419171 | 2.341788 | 2.427383 | 2.319155 | 2.450017 |
| 28 | 2.397837 | 0.033754 | 2017 | ASR | 2.375087 | 2.420587 | 2.36945 | 2.426224 | 2.362868 | 2.432806 | 2.354564 | 2.441109 | 2.331679 | 2.463994 |
| 29 | 2.41098 | 0.034342 | 2018 | ASR | 2.387833 | 2.434126 | 2.382098 | 2.439861 | 2.375401 | 2.446558 | 2.366953 | 2.455006 | 2.343668 | 2.478291 |
| 30 | 2.421118 | 0.035245 | 2019 | ASR | 2.397363 | 2.444874 | 2.391477 | 2.45076 | 2.384604 | 2.457632 | 2.375934 | 2.466303 | 2.352038 | 2.490199 |
| 31 | 2.419598 | 0.036638 | 2020 | ASR | 2.394904 | 2.444291 | 2.388785 | 2.45041 | 2.381641 | 2.457554 | 2.372628 | 2.466567 | 2.347788 | 2.491408 |
| 32 | 2.428711 | 0.039491 | 2021 | ASR | 2.402094 | 2.455328 | 2.395499 | 2.461923 | 2.387799 | 2.469623 | 2.378084 | 2.479338 | 2.351309 | 2.506113 |
| 33 | 2.438869 | 0.049353 | 2022 | ASR | 2.405605 | 2.472133 | 2.397363 | 2.480375 | 2.38774 | 2.489999 | 2.375599 | 2.50214 | 2.342138 | 2.535601 |
| 34 | 2.446612 | 0.054953 | 2023 | ASR | 2.409574 | 2.483651 | 2.400397 | 2.492828 | 2.389681 | 2.503544 | 2.376162 | 2.517062 | 2.338904 | 2.554321 |
| 35 | 2.453749 | 0.060408 | 2024 | ASR | 2.413034 | 2.494464 | 2.402946 | 2.504552 | 2.391167 | 2.516332 | 2.376306 | 2.531192 | 2.33535 | 2.572149 |
| 36 | 2.460256 | 0.06577 | 2025 | ASR | 2.415928 | 2.504585 | 2.404944 | 2.515569 | 2.392119 | 2.528394 | 2.37594 | 2.544573 | 2.331348 | 2.589165 |
| 37 | 2.466141 | 0.07108 | 2026 | ASR | 2.418233 | 2.514049 | 2.406363 | 2.52592 | 2.392502 | 2.53978 | 2.375017 | 2.557266 | 2.326824 | 2.605458 |
| 38 | 2.471354 | 0.07636 | 2027 | ASR | 2.419887 | 2.52282 | 2.407135 | 2.535573 | 2.392244 | 2.550463 | 2.37346 | 2.569247 | 2.321688 | 2.62102 |
| 39 | 2.475803 | 0.081593 | 2028 | ASR | 2.42081 | 2.530797 | 2.407184 | 2.544423 | 2.391273 | 2.560334 | 2.371201 | 2.580406 | 2.315881 | 2.635726 |
| 40 | 2.479463 | 0.086786 | 2029 | ASR | 2.420969 | 2.537957 | 2.406475 | 2.55245 | 2.389552 | 2.569373 | 2.368202 | 2.590723 | 2.309361 | 2.649564 |
| 41 | 2.482365 | 0.091962 | 2030 | ASR | 2.420382 | 2.544347 | 2.405025 | 2.559705 | 2.387092 | 2.577637 | 2.36447 | 2.60026 | 2.30212 | 2.66261 |
| 42 | 2.484576 | 0.097143 | 2031 | ASR | 2.419102 | 2.550051 | 2.402879 | 2.566273 | 2.383936 | 2.585216 | 2.360038 | 2.609114 | 2.294175 | 2.674977 |
| 43 | 2.486118 | 0.102343 | 2032 | ASR | 2.417139 | 2.555098 | 2.400048 | 2.572189 | 2.380091 | 2.592146 | 2.354914 | 2.617323 | 2.285526 | 2.686711 |
| 44 | 2.486972 | 0.107547 | 2033 | ASR | 2.414486 | 2.559459 | 2.396525 | 2.57742 | 2.375553 | 2.598391 | 2.349097 | 2.624848 | 2.27618 | 2.697765 |
| 45 | 2.487153 | 0.112762 | 2034 | ASR | 2.411151 | 2.563155 | 2.39232 | 2.581986 | 2.370331 | 2.603975 | 2.342592 | 2.631714 | 2.266139 | 2.708167 |
| 46 | 2.48672 | 0.11801 | 2035 | ASR | 2.407181 | 2.566259 | 2.387474 | 2.585967 | 2.364462 | 2.608979 | 2.335431 | 2.63801 | 2.25542 | 2.718021 |
| **Singapore** | | | | | | | | | | | | | | |
| 1 | 1.702921 | 0.124597 | 1990 | ASR | 1.618943 | 1.7869 | 1.598135 | 1.807707 | 1.573839 | 1.832004 | 1.543188 | 1.862655 | 1.458711 | 1.947131 |
| 2 | 1.691655 | 0.115238 | 1991 | ASR | 1.613984 | 1.769326 | 1.59474 | 1.788571 | 1.572268 | 1.811042 | 1.543919 | 1.839391 | 1.465788 | 1.917522 |
| 3 | 1.678695 | 0.106751 | 1992 | ASR | 1.606745 | 1.750646 | 1.588918 | 1.768473 | 1.568101 | 1.78929 | 1.54184 | 1.81555 | 1.469463 | 1.887928 |
| 4 | 1.665573 | 0.099178 | 1993 | ASR | 1.598728 | 1.732419 | 1.582165 | 1.748982 | 1.562825 | 1.768321 | 1.538428 | 1.792719 | 1.471185 | 1.859962 |
| 5 | 1.651902 | 0.092418 | 1994 | ASR | 1.589612 | 1.714192 | 1.574178 | 1.729626 | 1.556157 | 1.747647 | 1.533422 | 1.770382 | 1.470762 | 1.833042 |
| 6 | 1.637992 | 0.086406 | 1995 | ASR | 1.579754 | 1.69623 | 1.565324 | 1.71066 | 1.548475 | 1.727509 | 1.527219 | 1.748765 | 1.468636 | 1.807348 |
| 7 | 1.623745 | 0.081065 | 1996 | ASR | 1.569107 | 1.678383 | 1.555569 | 1.691921 | 1.539762 | 1.707729 | 1.51982 | 1.727671 | 1.464858 | 1.782633 |
| 8 | 1.609516 | 0.076352 | 1997 | ASR | 1.558054 | 1.660977 | 1.545304 | 1.673728 | 1.530415 | 1.688616 | 1.511632 | 1.707399 | 1.459866 | 1.759166 |
| 9 | 1.592998 | 0.0721 | 1998 | ASR | 1.544403 | 1.641593 | 1.532363 | 1.653634 | 1.518303 | 1.667693 | 1.500567 | 1.68543 | 1.451683 | 1.734313 |
| 10 | 1.577212 | 0.068382 | 1999 | ASR | 1.531123 | 1.623302 | 1.519703 | 1.634722 | 1.506369 | 1.648056 | 1.489547 | 1.664878 | 1.443184 | 1.711241 |
| 11 | 1.56095 | 0.065082 | 2000 | ASR | 1.517084 | 1.604815 | 1.506216 | 1.615684 | 1.493525 | 1.628375 | 1.477514 | 1.644385 | 1.433389 | 1.688511 |
| 12 | 1.543512 | 0.062146 | 2001 | ASR | 1.501626 | 1.585399 | 1.491247 | 1.595777 | 1.479129 | 1.607896 | 1.463841 | 1.623184 | 1.421706 | 1.665319 |
| 13 | 1.523973 | 0.059449 | 2002 | ASR | 1.483904 | 1.564041 | 1.473976 | 1.573969 | 1.462383 | 1.585562 | 1.447759 | 1.600186 | 1.407453 | 1.640493 |
| 14 | 1.504526 | 0.057099 | 2003 | ASR | 1.466042 | 1.543011 | 1.456507 | 1.552546 | 1.445372 | 1.563681 | 1.431326 | 1.577727 | 1.392613 | 1.61644 |
| 15 | 1.482346 | 0.054909 | 2004 | ASR | 1.445338 | 1.519355 | 1.436168 | 1.528525 | 1.425461 | 1.539232 | 1.411953 | 1.552739 | 1.374725 | 1.589968 |
| 16 | 1.460136 | 0.052994 | 2005 | ASR | 1.424418 | 1.495854 | 1.415568 | 1.504704 | 1.405234 | 1.515038 | 1.392198 | 1.528074 | 1.356268 | 1.564004 |
| 17 | 1.439845 | 0.051406 | 2006 | ASR | 1.405197 | 1.474492 | 1.396613 | 1.483077 | 1.386588 | 1.493101 | 1.373943 | 1.505747 | 1.339089 | 1.5406 |
| 18 | 1.416026 | 0.049917 | 2007 | ASR | 1.382382 | 1.44967 | 1.374046 | 1.458007 | 1.364312 | 1.46774 | 1.352032 | 1.48002 | 1.318188 | 1.513864 |
| 19 | 1.391071 | 0.048612 | 2008 | ASR | 1.358306 | 1.423835 | 1.350188 | 1.431953 | 1.340709 | 1.441432 | 1.32875 | 1.453391 | 1.295792 | 1.486349 |
| 20 | 1.366251 | 0.047561 | 2009 | ASR | 1.334195 | 1.398307 | 1.326252 | 1.406249 | 1.316978 | 1.415523 | 1.305278 | 1.427223 | 1.273032 | 1.45947 |
| 21 | 1.340784 | 0.046713 | 2010 | ASR | 1.3093 | 1.372269 | 1.301499 | 1.38007 | 1.292389 | 1.389179 | 1.280898 | 1.400671 | 1.249227 | 1.432342 |
| 22 | 1.317011 | 0.046166 | 2011 | ASR | 1.285895 | 1.348127 | 1.278185 | 1.355836 | 1.269183 | 1.364839 | 1.257826 | 1.376195 | 1.226525 | 1.407496 |
| 23 | 1.291864 | 0.045794 | 2012 | ASR | 1.260999 | 1.322729 | 1.253351 | 1.330377 | 1.244421 | 1.339307 | 1.233156 | 1.350572 | 1.202107 | 1.381621 |
| 24 | 1.264543 | 0.045567 | 2013 | ASR | 1.233831 | 1.295255 | 1.226221 | 1.302865 | 1.217335 | 1.311751 | 1.206126 | 1.32296 | 1.175231 | 1.353855 |
| 25 | 1.238295 | 0.045603 | 2014 | ASR | 1.207559 | 1.269031 | 1.199943 | 1.276647 | 1.191051 | 1.285539 | 1.179832 | 1.296758 | 1.148914 | 1.327676 |
| 26 | 1.2121 | 0.045864 | 2015 | ASR | 1.181188 | 1.243012 | 1.173529 | 1.250672 | 1.164585 | 1.259615 | 1.153303 | 1.270897 | 1.122207 | 1.301993 |
| 27 | 1.185402 | 0.046319 | 2016 | ASR | 1.154183 | 1.216621 | 1.146448 | 1.224357 | 1.137416 | 1.233389 | 1.126022 | 1.244783 | 1.094617 | 1.276187 |
| 28 | 1.160649 | 0.047082 | 2017 | ASR | 1.128916 | 1.192383 | 1.121053 | 1.200245 | 1.111872 | 1.209426 | 1.10029 | 1.221009 | 1.068368 | 1.25293 |
| 29 | 1.134745 | 0.048014 | 2018 | ASR | 1.102383 | 1.167106 | 1.094365 | 1.175124 | 1.085003 | 1.184487 | 1.073191 | 1.196298 | 1.040638 | 1.228851 |
| 30 | 1.108212 | 0.049127 | 2019 | ASR | 1.0751 | 1.141324 | 1.066896 | 1.149528 | 1.057316 | 1.159108 | 1.045231 | 1.171193 | 1.011923 | 1.204501 |
| 31 | 1.082421 | 0.050498 | 2020 | ASR | 1.048385 | 1.116457 | 1.039952 | 1.12489 | 1.030105 | 1.134737 | 1.017682 | 1.14716 | 0.983445 | 1.181398 |
| 32 | 1.056012 | 0.052061 | 2021 | ASR | 1.020923 | 1.091101 | 1.012228 | 1.099795 | 1.002077 | 1.109947 | 0.98927 | 1.122754 | 0.953972 | 1.158051 |
| 33 | 1.029748 | 0.0552 | 2022 | ASR | 0.992543 | 1.066952 | 0.983324 | 1.076171 | 0.97256 | 1.086935 | 0.958981 | 1.100514 | 0.921555 | 1.13794 |
| 34 | 1.003574 | 0.05707 | 2023 | ASR | 0.965109 | 1.042039 | 0.955578 | 1.05157 | 0.94445 | 1.062698 | 0.93041 | 1.076738 | 0.891717 | 1.115431 |
| 35 | 0.977453 | 0.058867 | 2024 | ASR | 0.937777 | 1.01713 | 0.927946 | 1.026961 | 0.916467 | 1.03844 | 0.901986 | 1.052921 | 0.862074 | 1.092833 |
| 36 | 0.951434 | 0.060589 | 2025 | ASR | 0.910597 | 0.992271 | 0.900479 | 1.002389 | 0.888664 | 1.014204 | 0.873759 | 1.029109 | 0.83268 | 1.070188 |
| 37 | 0.925556 | 0.062234 | 2026 | ASR | 0.88361 | 0.967502 | 0.873217 | 0.977895 | 0.861082 | 0.99003 | 0.845772 | 1.00534 | 0.803578 | 1.047534 |
| 38 | 0.89985 | 0.063799 | 2027 | ASR | 0.856849 | 0.94285 | 0.846195 | 0.953504 | 0.833754 | 0.965945 | 0.818059 | 0.98164 | 0.774804 | 1.024895 |
| 39 | 0.874335 | 0.065278 | 2028 | ASR | 0.830338 | 0.918332 | 0.819436 | 0.929234 | 0.806707 | 0.941963 | 0.790649 | 0.958021 | 0.74639 | 1.00228 |
| 40 | 0.849046 | 0.066671 | 2029 | ASR | 0.80411 | 0.893982 | 0.792976 | 0.905116 | 0.779975 | 0.918117 | 0.763574 | 0.934518 | 0.718371 | 0.979721 |
| 41 | 0.824024 | 0.067983 | 2030 | ASR | 0.778203 | 0.869845 | 0.76685 | 0.881198 | 0.753593 | 0.894455 | 0.73687 | 0.911179 | 0.690777 | 0.957271 |
| 42 | 0.799304 | 0.069219 | 2031 | ASR | 0.75265 | 0.845958 | 0.74109 | 0.857517 | 0.727593 | 0.871015 | 0.710565 | 0.888043 | 0.663634 | 0.934974 |
| 43 | 0.774912 | 0.070383 | 2032 | ASR | 0.727474 | 0.82235 | 0.71572 | 0.834104 | 0.701996 | 0.847829 | 0.684681 | 0.865143 | 0.636962 | 0.912863 |
| 44 | 0.750864 | 0.071474 | 2033 | ASR | 0.702691 | 0.799037 | 0.690755 | 0.810973 | 0.676817 | 0.824911 | 0.659235 | 0.842493 | 0.610776 | 0.890952 |
| 45 | 0.72718 | 0.072495 | 2034 | ASR | 0.678318 | 0.776042 | 0.666212 | 0.788149 | 0.652075 | 0.802285 | 0.634241 | 0.820119 | 0.585089 | 0.869271 |
| 46 | 0.703891 | 0.073454 | 2035 | ASR | 0.654383 | 0.753399 | 0.642116 | 0.765666 | 0.627792 | 0.77999 | 0.609723 | 0.798059 | 0.559921 | 0.847861 |
| **Thailand** | | | | | | | | | | | | | | |
| 1 | 3.515137 | 0.097894 | 1990 | ASR | 3.449156 | 3.581117 | 3.432808 | 3.597465 | 3.413719 | 3.616555 | 3.389637 | 3.640636 | 3.323265 | 3.707008 |
| 2 | 3.502286 | 0.084426 | 1991 | ASR | 3.445383 | 3.559189 | 3.431284 | 3.573288 | 3.414821 | 3.589751 | 3.394053 | 3.61052 | 3.336812 | 3.66776 |
| 3 | 3.500676 | 0.08093 | 1992 | ASR | 3.446129 | 3.555223 | 3.432614 | 3.568738 | 3.416832 | 3.58452 | 3.396924 | 3.604428 | 3.342053 | 3.659299 |
| 4 | 3.498517 | 0.079316 | 1993 | ASR | 3.445058 | 3.551976 | 3.431812 | 3.565222 | 3.416345 | 3.580688 | 3.396833 | 3.6002 | 3.343057 | 3.653976 |
| 5 | 3.486844 | 0.077931 | 1994 | ASR | 3.434319 | 3.53937 | 3.421304 | 3.552385 | 3.406108 | 3.567581 | 3.386937 | 3.586752 | 3.3341 | 3.639589 |
| 6 | 3.462976 | 0.076452 | 1995 | ASR | 3.411447 | 3.514505 | 3.39868 | 3.527273 | 3.383772 | 3.542181 | 3.364965 | 3.560988 | 3.31313 | 3.612822 |
| 7 | 3.428083 | 0.074847 | 1996 | ASR | 3.377636 | 3.47853 | 3.365137 | 3.49103 | 3.350542 | 3.505625 | 3.33213 | 3.524037 | 3.281383 | 3.574783 |
| 8 | 3.365377 | 0.07278 | 1997 | ASR | 3.316323 | 3.414431 | 3.304169 | 3.426585 | 3.289977 | 3.440778 | 3.272073 | 3.458682 | 3.222728 | 3.508027 |
| 9 | 3.321424 | 0.071087 | 1998 | ASR | 3.273511 | 3.369336 | 3.26164 | 3.381208 | 3.247778 | 3.395069 | 3.23029 | 3.412557 | 3.182094 | 3.460754 |
| 10 | 3.292308 | 0.069668 | 1999 | ASR | 3.245352 | 3.339264 | 3.233717 | 3.350898 | 3.220132 | 3.364484 | 3.202993 | 3.381622 | 3.155759 | 3.428857 |
| 11 | 3.281914 | 0.068521 | 2000 | ASR | 3.235731 | 3.328097 | 3.224288 | 3.33954 | 3.210926 | 3.352902 | 3.19407 | 3.369758 | 3.147612 | 3.416216 |
| 12 | 3.287113 | 0.06759 | 2001 | ASR | 3.241557 | 3.332668 | 3.23027 | 3.343956 | 3.21709 | 3.357136 | 3.200463 | 3.373763 | 3.154637 | 3.419589 |
| 13 | 3.293022 | 0.066723 | 2002 | ASR | 3.248051 | 3.337993 | 3.236908 | 3.349136 | 3.223897 | 3.362147 | 3.207484 | 3.378561 | 3.162245 | 3.423799 |
| 14 | 3.270997 | 0.06544 | 2003 | ASR | 3.22689 | 3.315103 | 3.215962 | 3.326032 | 3.203201 | 3.338792 | 3.187103 | 3.354891 | 3.142735 | 3.399259 |
| 15 | 3.243791 | 0.064268 | 2004 | ASR | 3.200474 | 3.287108 | 3.189742 | 3.297841 | 3.177209 | 3.310373 | 3.161399 | 3.326183 | 3.117826 | 3.369757 |
| 16 | 3.185253 | 0.062933 | 2005 | ASR | 3.142836 | 3.22767 | 3.132326 | 3.23818 | 3.120054 | 3.250451 | 3.104573 | 3.265933 | 3.061904 | 3.308601 |
| 17 | 2.990418 | 0.058703 | 2006 | ASR | 2.950852 | 3.029983 | 2.941049 | 3.039787 | 2.929602 | 3.051234 | 2.915161 | 3.065675 | 2.875361 | 3.105475 |
| 18 | 2.809947 | 0.055227 | 2007 | ASR | 2.772724 | 2.84717 | 2.763501 | 2.856393 | 2.752732 | 2.867162 | 2.739146 | 2.880748 | 2.701702 | 2.918191 |
| 19 | 2.64799 | 0.052106 | 2008 | ASR | 2.612871 | 2.68311 | 2.604169 | 2.691811 | 2.594009 | 2.701972 | 2.581191 | 2.71479 | 2.545863 | 2.750118 |
| 20 | 2.501489 | 0.049228 | 2009 | ASR | 2.46831 | 2.534669 | 2.460089 | 2.54289 | 2.450489 | 2.552489 | 2.438379 | 2.564599 | 2.405003 | 2.597976 |
| 21 | 2.385989 | 0.046814 | 2010 | ASR | 2.354436 | 2.417541 | 2.346618 | 2.425359 | 2.337489 | 2.434488 | 2.325973 | 2.446004 | 2.294233 | 2.477744 |
| 22 | 2.270026 | 0.044451 | 2011 | ASR | 2.240066 | 2.299986 | 2.232643 | 2.307409 | 2.223975 | 2.316077 | 2.21304 | 2.327012 | 2.182903 | 2.357149 |
| 23 | 2.163913 | 0.042259 | 2012 | ASR | 2.13543 | 2.192396 | 2.128373 | 2.199453 | 2.120132 | 2.207693 | 2.109736 | 2.218089 | 2.081085 | 2.246741 |
| 24 | 2.076357 | 0.040356 | 2013 | ASR | 2.049157 | 2.103557 | 2.042417 | 2.110297 | 2.034548 | 2.118166 | 2.02462 | 2.128094 | 1.997259 | 2.155455 |
| 25 | 2.020159 | 0.038869 | 2014 | ASR | 1.993961 | 2.046357 | 1.98747 | 2.052848 | 1.97989 | 2.060427 | 1.970329 | 2.069989 | 1.943975 | 2.096342 |
| 26 | 1.982388 | 0.037666 | 2015 | ASR | 1.957001 | 2.007775 | 1.950711 | 2.014065 | 1.943366 | 2.02141 | 1.9341 | 2.030676 | 1.908563 | 2.056213 |
| 27 | 1.952666 | 0.036631 | 2016 | ASR | 1.927976 | 1.977355 | 1.921859 | 1.983473 | 1.914716 | 1.990616 | 1.905704 | 1.999627 | 1.880868 | 2.024463 |
| 28 | 1.946958 | 0.035914 | 2017 | ASR | 1.922752 | 1.971164 | 1.916754 | 1.977162 | 1.909751 | 1.984165 | 1.900916 | 1.993 | 1.876566 | 2.017349 |
| 29 | 1.974974 | 0.035603 | 2018 | ASR | 1.950978 | 1.998971 | 1.945032 | 2.004917 | 1.938089 | 2.011859 | 1.929331 | 2.020618 | 1.905192 | 2.044757 |
| 30 | 2.005899 | 0.035435 | 2019 | ASR | 1.982016 | 2.029782 | 1.976099 | 2.0357 | 1.969189 | 2.04261 | 1.960472 | 2.051327 | 1.936447 | 2.075352 |
| 31 | 2.013163 | 0.035246 | 2020 | ASR | 1.989408 | 2.036919 | 1.983521 | 2.042805 | 1.976649 | 2.049677 | 1.967978 | 2.058348 | 1.944082 | 2.082244 |
| 32 | 2.025223 | 0.038299 | 2021 | ASR | 1.99941 | 2.051037 | 1.993014 | 2.057433 | 1.985546 | 2.064901 | 1.976125 | 2.074322 | 1.950158 | 2.100289 |
| 33 | 2.013631 | 0.081742 | 2022 | ASR | 1.958537 | 2.068724 | 1.944886 | 2.082375 | 1.928946 | 2.098315 | 1.908838 | 2.118423 | 1.853417 | 2.173844 |
| 34 | 2.008119 | 0.108324 | 2023 | ASR | 1.935109 | 2.081129 | 1.917019 | 2.099219 | 1.895896 | 2.120342 | 1.869248 | 2.14699 | 1.795805 | 2.220433 |
| 35 | 2.004044 | 0.130603 | 2024 | ASR | 1.916017 | 2.09207 | 1.894207 | 2.113881 | 1.868739 | 2.139348 | 1.836611 | 2.171477 | 1.748062 | 2.260025 |
| 36 | 2.00128 | 0.150568 | 2025 | ASR | 1.899797 | 2.102763 | 1.874653 | 2.127907 | 1.845292 | 2.157268 | 1.808252 | 2.194308 | 1.706167 | 2.296393 |
| 37 | 1.999713 | 0.169109 | 2026 | ASR | 1.885733 | 2.113692 | 1.857492 | 2.141933 | 1.824516 | 2.17491 | 1.782915 | 2.21651 | 1.668259 | 2.331166 |
| 38 | 1.999255 | 0.186717 | 2027 | ASR | 1.873407 | 2.125102 | 1.842226 | 2.156284 | 1.805816 | 2.192694 | 1.759883 | 2.238626 | 1.633289 | 2.365221 |
| 39 | 1.999857 | 0.203669 | 2028 | ASR | 1.862584 | 2.13713 | 1.828572 | 2.171143 | 1.788856 | 2.210858 | 1.738754 | 2.260961 | 1.600666 | 2.399049 |
| 40 | 2.001477 | 0.220161 | 2029 | ASR | 1.853088 | 2.149865 | 1.816321 | 2.186632 | 1.77339 | 2.229564 | 1.71923 | 2.283723 | 1.569961 | 2.432993 |
| 41 | 2.004091 | 0.236348 | 2030 | ASR | 1.844793 | 2.163389 | 1.805323 | 2.202859 | 1.759235 | 2.248947 | 1.701093 | 2.307089 | 1.540849 | 2.467333 |
| 42 | 2.0077 | 0.252356 | 2031 | ASR | 1.837613 | 2.177788 | 1.795469 | 2.219932 | 1.74626 | 2.269141 | 1.684181 | 2.33122 | 1.513083 | 2.502317 |
| 43 | 2.012288 | 0.268286 | 2032 | ASR | 1.831463 | 2.193113 | 1.786659 | 2.237917 | 1.734343 | 2.290233 | 1.668345 | 2.356231 | 1.486447 | 2.538129 |
| 44 | 2.017789 | 0.284195 | 2033 | ASR | 1.826242 | 2.209337 | 1.778782 | 2.256797 | 1.723364 | 2.312215 | 1.653452 | 2.382127 | 1.460768 | 2.574811 |
| 45 | 2.024197 | 0.300142 | 2034 | ASR | 1.821901 | 2.226492 | 1.771777 | 2.276616 | 1.71325 | 2.335144 | 1.639415 | 2.408979 | 1.435919 | 2.612475 |
| 46 | 2.031543 | 0.316196 | 2035 | ASR | 1.818427 | 2.244659 | 1.765622 | 2.297464 | 1.703964 | 2.359122 | 1.62618 | 2.436906 | 1.411799 | 2.651287 |
| **VietNam** | | | | | | | | | | | | | | |
| 1 | 8.323067 | 0.098571 | 1990 | ASR | 8.25663 | 8.389503 | 8.240168 | 8.405965 | 8.220947 | 8.425186 | 8.196699 | 8.449434 | 8.129868 | 8.516265 |
| 2 | 8.362953 | 0.090249 | 1991 | ASR | 8.302126 | 8.423781 | 8.287054 | 8.438852 | 8.269456 | 8.456451 | 8.247255 | 8.478652 | 8.186066 | 8.53984 |
| 3 | 8.403328 | 0.085205 | 1992 | ASR | 8.3459 | 8.460756 | 8.331671 | 8.474985 | 8.315056 | 8.4916 | 8.294096 | 8.512561 | 8.236327 | 8.570329 |
| 4 | 8.442509 | 0.082172 | 1993 | ASR | 8.387125 | 8.497893 | 8.373402 | 8.511615 | 8.357379 | 8.527639 | 8.337164 | 8.547853 | 8.281452 | 8.603566 |
| 5 | 8.484421 | 0.080367 | 1994 | ASR | 8.430254 | 8.538588 | 8.416833 | 8.55201 | 8.401161 | 8.567681 | 8.381391 | 8.587451 | 8.326903 | 8.64194 |
| 6 | 8.52195 | 0.079235 | 1995 | ASR | 8.468546 | 8.575355 | 8.455314 | 8.588587 | 8.439863 | 8.604038 | 8.420371 | 8.62353 | 8.36665 | 8.677251 |
| 7 | 8.552287 | 0.078482 | 1996 | ASR | 8.49939 | 8.605184 | 8.486284 | 8.61829 | 8.47098 | 8.633594 | 8.451673 | 8.652901 | 8.398462 | 8.706111 |
| 8 | 8.577822 | 0.077948 | 1997 | ASR | 8.525285 | 8.63036 | 8.512268 | 8.643377 | 8.497068 | 8.658577 | 8.477892 | 8.677752 | 8.425043 | 8.730601 |
| 9 | 8.595993 | 0.0775 | 1998 | ASR | 8.543758 | 8.648229 | 8.530816 | 8.661171 | 8.515703 | 8.676284 | 8.496638 | 8.695349 | 8.444093 | 8.747894 |
| 10 | 8.615297 | 0.077134 | 1999 | ASR | 8.563309 | 8.667285 | 8.550427 | 8.680166 | 8.535386 | 8.695207 | 8.516412 | 8.714182 | 8.464115 | 8.766479 |
| 11 | 8.632305 | 0.076757 | 2000 | ASR | 8.580571 | 8.684039 | 8.567752 | 8.696858 | 8.552785 | 8.711825 | 8.533902 | 8.730707 | 8.481861 | 8.782749 |
| 12 | 8.665618 | 0.076485 | 2001 | ASR | 8.614067 | 8.717169 | 8.601294 | 8.729942 | 8.58638 | 8.744856 | 8.567564 | 8.763671 | 8.515708 | 8.815528 |
| 13 | 8.698062 | 0.076229 | 2002 | ASR | 8.646684 | 8.74944 | 8.633953 | 8.76217 | 8.619089 | 8.777035 | 8.600336 | 8.795787 | 8.548653 | 8.84747 |
| 14 | 8.72155 | 0.075925 | 2003 | ASR | 8.670377 | 8.772724 | 8.657698 | 8.785403 | 8.642892 | 8.800209 | 8.624215 | 8.818886 | 8.572738 | 8.870363 |
| 15 | 8.735906 | 0.075539 | 2004 | ASR | 8.684993 | 8.786819 | 8.672378 | 8.799434 | 8.657647 | 8.814164 | 8.639065 | 8.832747 | 8.587849 | 8.883963 |
| 16 | 8.739067 | 0.075038 | 2005 | ASR | 8.688491 | 8.789643 | 8.67596 | 8.802175 | 8.661327 | 8.816807 | 8.642868 | 8.835267 | 8.591992 | 8.886143 |
| 17 | 8.732411 | 0.074471 | 2006 | ASR | 8.682217 | 8.782605 | 8.669781 | 8.795041 | 8.655259 | 8.809563 | 8.636939 | 8.827883 | 8.586448 | 8.878374 |
| 18 | 8.718982 | 0.073897 | 2007 | ASR | 8.669175 | 8.768788 | 8.656834 | 8.781129 | 8.642425 | 8.795539 | 8.624246 | 8.813717 | 8.574144 | 8.863819 |
| 19 | 8.706259 | 0.073355 | 2008 | ASR | 8.656818 | 8.7557 | 8.644568 | 8.76795 | 8.630264 | 8.782254 | 8.612218 | 8.800299 | 8.562484 | 8.850034 |
| 20 | 8.702245 | 0.072834 | 2009 | ASR | 8.653155 | 8.751335 | 8.640991 | 8.763499 | 8.626789 | 8.777701 | 8.608871 | 8.795618 | 8.55949 | 8.845 |
| 21 | 8.70348 | 0.072313 | 2010 | ASR | 8.654741 | 8.752219 | 8.642664 | 8.764295 | 8.628563 | 8.778396 | 8.610774 | 8.796185 | 8.561746 | 8.845213 |
| 22 | 8.711383 | 0.071809 | 2011 | ASR | 8.662983 | 8.759782 | 8.650991 | 8.771774 | 8.636988 | 8.785777 | 8.619323 | 8.803442 | 8.570637 | 8.852128 |
| 23 | 8.715112 | 0.0713 | 2012 | ASR | 8.667056 | 8.763167 | 8.655149 | 8.775074 | 8.641245 | 8.788978 | 8.623706 | 8.806518 | 8.575364 | 8.854859 |
| 24 | 8.717771 | 0.07082 | 2013 | ASR | 8.670039 | 8.765504 | 8.658212 | 8.777331 | 8.644402 | 8.791141 | 8.62698 | 8.808562 | 8.578965 | 8.856578 |
| 25 | 8.728827 | 0.070464 | 2014 | ASR | 8.681334 | 8.77632 | 8.669566 | 8.788087 | 8.655826 | 8.801828 | 8.638492 | 8.819162 | 8.590717 | 8.866937 |
| 26 | 8.735329 | 0.070194 | 2015 | ASR | 8.688018 | 8.78264 | 8.676296 | 8.794363 | 8.662608 | 8.808051 | 8.64534 | 8.825319 | 8.597748 | 8.872911 |
| 27 | 8.729305 | 0.069934 | 2016 | ASR | 8.682169 | 8.77644 | 8.67049 | 8.788119 | 8.656853 | 8.801756 | 8.63965 | 8.81896 | 8.592235 | 8.866375 |
| 28 | 8.715759 | 0.069791 | 2017 | ASR | 8.66872 | 8.762799 | 8.657065 | 8.774454 | 8.643455 | 8.788063 | 8.626287 | 8.805232 | 8.578968 | 8.852551 |
| 29 | 8.688911 | 0.069832 | 2018 | ASR | 8.641844 | 8.735978 | 8.630182 | 8.74764 | 8.616565 | 8.761257 | 8.599386 | 8.778436 | 8.55204 | 8.825782 |
| 30 | 8.654415 | 0.070399 | 2019 | ASR | 8.606966 | 8.701864 | 8.595209 | 8.71362 | 8.581482 | 8.727348 | 8.564163 | 8.744666 | 8.516433 | 8.792397 |
| 31 | 8.621968 | 0.072247 | 2020 | ASR | 8.573274 | 8.670663 | 8.561209 | 8.682728 | 8.547121 | 8.696816 | 8.529348 | 8.714589 | 8.480365 | 8.763572 |
| 32 | 8.56892 | 0.076508 | 2021 | ASR | 8.517354 | 8.620487 | 8.504577 | 8.633263 | 8.489658 | 8.648182 | 8.470838 | 8.667003 | 8.418965 | 8.718875 |
| 33 | 8.531087 | 0.106669 | 2022 | ASR | 8.459192 | 8.602982 | 8.441378 | 8.620796 | 8.420577 | 8.641596 | 8.394337 | 8.667837 | 8.322015 | 8.740159 |
| 34 | 8.491012 | 0.118774 | 2023 | ASR | 8.410958 | 8.571065 | 8.391123 | 8.5909 | 8.367962 | 8.614061 | 8.338744 | 8.64328 | 8.258215 | 8.723808 |
| 35 | 8.448874 | 0.130166 | 2024 | ASR | 8.361143 | 8.536606 | 8.339405 | 8.558343 | 8.314023 | 8.583726 | 8.282002 | 8.615746 | 8.19375 | 8.703999 |
| 36 | 8.404828 | 0.141061 | 2025 | ASR | 8.309753 | 8.499903 | 8.286196 | 8.52346 | 8.258689 | 8.550967 | 8.223989 | 8.585668 | 8.128349 | 8.681307 |
| 37 | 8.35889 | 0.151634 | 2026 | ASR | 8.256688 | 8.461091 | 8.231365 | 8.486414 | 8.201797 | 8.515982 | 8.164495 | 8.553284 | 8.061687 | 8.656092 |
| 38 | 8.31151 | 0.161976 | 2027 | ASR | 8.202338 | 8.420682 | 8.175288 | 8.447732 | 8.143702 | 8.479317 | 8.103856 | 8.519163 | 7.994036 | 8.628983 |
| 39 | 8.263116 | 0.172004 | 2028 | ASR | 8.147185 | 8.379046 | 8.118461 | 8.407771 | 8.08492 | 8.441312 | 8.042607 | 8.483625 | 7.925989 | 8.600243 |
| 40 | 8.214178 | 0.181751 | 2029 | ASR | 8.091677 | 8.336678 | 8.061325 | 8.36703 | 8.025883 | 8.402472 | 7.981173 | 8.447183 | 7.857945 | 8.57041 |
| 41 | 8.165018 | 0.191315 | 2030 | ASR | 8.036071 | 8.293964 | 8.004121 | 8.325914 | 7.966815 | 8.36322 | 7.919751 | 8.410284 | 7.790039 | 8.539996 |
| 42 | 8.115844 | 0.200798 | 2031 | ASR | 7.980506 | 8.251182 | 7.946972 | 8.284715 | 7.907817 | 8.323871 | 7.85842 | 8.373267 | 7.722279 | 8.509409 |
| 43 | 8.067266 | 0.210265 | 2032 | ASR | 7.925547 | 8.208984 | 7.890433 | 8.244098 | 7.849431 | 8.2851 | 7.797706 | 8.336825 | 7.655147 | 8.479385 |
| 44 | 8.019677 | 0.219653 | 2033 | ASR | 7.871631 | 8.167723 | 7.834949 | 8.204405 | 7.792117 | 8.247237 | 7.738083 | 8.301272 | 7.589158 | 8.450196 |
| 45 | 7.973518 | 0.228992 | 2034 | ASR | 7.819178 | 8.127859 | 7.780936 | 8.1661 | 7.736283 | 8.210753 | 7.679951 | 8.267085 | 7.524695 | 8.422342 |
| 46 | 7.929111 | 0.238363 | 2035 | ASR | 7.768455 | 8.089768 | 7.728648 | 8.129575 | 7.682167 | 8.176056 | 7.62353 | 8.234693 | 7.46192 | 8.396303 |

**Table S2: Prediction results of BAPC ASDR in the world, China and ASEAN countries to 2035**

|  | **val** | **sd** | **Time** | **group** | **low_50** | **up_50** | **low_60** | **up_60** | **low_70** | **up_70** | **low_80** | **up_80** | **low_95** | **up_95** |
| --- | --- | --- | --- | --- | --- | --- | --- | --- | --- | --- | --- | --- | --- | --- |
| **Global** | | | | | | | | | | | | | | |
| 1 | 143.7244 | 0.06761 | 1990 | ASR | 143.6788 | 143.7699 | 143.6675 | 143.7812 | 143.6543 | 143.7944 | 143.6377 | 143.811 | 143.5919 | 143.8569 |
| 2 | 143.0597 | 0.066328 | 1991 | ASR | 143.0149 | 143.1044 | 143.0039 | 143.1154 | 142.9909 | 143.1284 | 142.9746 | 143.1447 | 142.9297 | 143.1897 |
| 3 | 142.6781 | 0.065157 | 1992 | ASR | 142.6341 | 142.722 | 142.6233 | 142.7329 | 142.6106 | 142.7456 | 142.5945 | 142.7616 | 142.5504 | 142.8058 |
| 4 | 142.5641 | 0.064125 | 1993 | ASR | 142.5209 | 142.6074 | 142.5102 | 142.6181 | 142.4977 | 142.6306 | 142.4819 | 142.6464 | 142.4385 | 142.6898 |
| 5 | 142.29 | 0.063078 | 1994 | ASR | 142.2475 | 142.3325 | 142.2369 | 142.343 | 142.2246 | 142.3553 | 142.2091 | 142.3708 | 142.1663 | 142.4136 |
| 6 | 142.8311 | 0.062297 | 1995 | ASR | 142.7891 | 142.8731 | 142.7787 | 142.8835 | 142.7666 | 142.8956 | 142.7512 | 142.911 | 142.709 | 142.9532 |
| 7 | 142.3885 | 0.061314 | 1996 | ASR | 142.3472 | 142.4298 | 142.3369 | 142.4401 | 142.325 | 142.452 | 142.3099 | 142.4671 | 142.2683 | 142.5087 |
| 8 | 141.6022 | 0.060264 | 1997 | ASR | 141.5616 | 141.6429 | 141.5516 | 141.6529 | 141.5398 | 141.6647 | 141.525 | 141.6795 | 141.4841 | 141.7204 |
| 9 | 141.98 | 0.059495 | 1998 | ASR | 141.9399 | 142.0201 | 141.93 | 142.03 | 141.9184 | 142.0416 | 141.9037 | 142.0563 | 141.8634 | 142.0966 |
| 10 | 141.7571 | 0.058598 | 1999 | ASR | 141.7176 | 141.7966 | 141.7078 | 141.8064 | 141.6964 | 141.8178 | 141.682 | 141.8322 | 141.6423 | 141.872 |
| 11 | 142.0412 | 0.057815 | 2000 | ASR | 142.0022 | 142.0802 | 141.9926 | 142.0898 | 141.9813 | 142.1011 | 141.9671 | 142.1153 | 141.9279 | 142.1545 |
| 12 | 142.8559 | 0.05716 | 2001 | ASR | 142.8173 | 142.8944 | 142.8078 | 142.9039 | 142.7967 | 142.9151 | 142.7826 | 142.9291 | 142.7438 | 142.9679 |
| 13 | 142.9359 | 0.05638 | 2002 | ASR | 142.8979 | 142.9739 | 142.8885 | 142.9834 | 142.8775 | 142.9944 | 142.8637 | 143.0082 | 142.8254 | 143.0465 |
| 14 | 143.0625 | 0.055667 | 2003 | ASR | 143.025 | 143.1 | 143.0157 | 143.1093 | 143.0049 | 143.1202 | 142.9912 | 143.1339 | 142.9534 | 143.1716 |
| 15 | 142.0014 | 0.054684 | 2004 | ASR | 141.9645 | 142.0382 | 141.9554 | 142.0474 | 141.9447 | 142.058 | 141.9313 | 142.0715 | 141.8942 | 142.1086 |
| 16 | 141.138 | 0.05369 | 2005 | ASR | 141.1018 | 141.1742 | 141.0929 | 141.1832 | 141.0824 | 141.1936 | 141.0692 | 141.2068 | 141.0328 | 141.2432 |
| 17 | 140.2707 | 0.052677 | 2006 | ASR | 140.2352 | 140.3062 | 140.2264 | 140.315 | 140.2162 | 140.3253 | 140.2032 | 140.3383 | 140.1675 | 140.374 |
| 18 | 140.3584 | 0.051842 | 2007 | ASR | 140.3234 | 140.3933 | 140.3148 | 140.402 | 140.3047 | 140.4121 | 140.2919 | 140.4248 | 140.2568 | 140.46 |
| 19 | 140.3794 | 0.051007 | 2008 | ASR | 140.345 | 140.4138 | 140.3365 | 140.4223 | 140.3266 | 140.4323 | 140.314 | 140.4448 | 140.2794 | 140.4794 |
| 20 | 139.1439 | 0.049923 | 2009 | ASR | 139.1103 | 139.1776 | 139.1019 | 139.1859 | 139.0922 | 139.1956 | 139.0799 | 139.2079 | 139.0461 | 139.2418 |
| 21 | 138.5202 | 0.048971 | 2010 | ASR | 138.4872 | 138.5532 | 138.479 | 138.5614 | 138.4695 | 138.5709 | 138.4574 | 138.583 | 138.4242 | 138.6162 |
| 22 | 138.064 | 0.048059 | 2011 | ASR | 138.0316 | 138.0963 | 138.0235 | 138.1044 | 138.0142 | 138.1137 | 138.0023 | 138.1256 | 137.9698 | 138.1581 |
| 23 | 137.6122 | 0.047169 | 2012 | ASR | 137.5804 | 137.6439 | 137.5725 | 137.6518 | 137.5633 | 137.661 | 137.5517 | 137.6726 | 137.5197 | 137.7046 |
| 24 | 138.3876 | 0.046571 | 2013 | ASR | 138.3562 | 138.419 | 138.3484 | 138.4268 | 138.3393 | 138.4358 | 138.3279 | 138.4473 | 138.2963 | 138.4789 |
| 25 | 138.7333 | 0.045904 | 2014 | ASR | 138.7024 | 138.7642 | 138.6947 | 138.7719 | 138.6857 | 138.7809 | 138.6745 | 138.7922 | 138.6433 | 138.8233 |
| 26 | 138.2288 | 0.045084 | 2015 | ASR | 138.1984 | 138.2591 | 138.1908 | 138.2667 | 138.182 | 138.2755 | 138.171 | 138.2866 | 138.1404 | 138.3171 |
| 27 | 138.1865 | 0.044343 | 2016 | ASR | 138.1566 | 138.2164 | 138.1492 | 138.2238 | 138.1405 | 138.2324 | 138.1296 | 138.2433 | 138.0996 | 138.2734 |
| 28 | 138.3019 | 0.043638 | 2017 | ASR | 138.2725 | 138.3314 | 138.2652 | 138.3386 | 138.2567 | 138.3472 | 138.246 | 138.3579 | 138.2164 | 138.3875 |
| 29 | 138.2315 | 0.04292 | 2018 | ASR | 138.2026 | 138.2605 | 138.1954 | 138.2676 | 138.1871 | 138.276 | 138.1765 | 138.2866 | 138.1474 | 138.3157 |
| 30 | 138.1936 | 0.042225 | 2019 | ASR | 138.1651 | 138.222 | 138.1581 | 138.2291 | 138.1498 | 138.2373 | 138.1394 | 138.2477 | 138.1108 | 138.2763 |
| 31 | 138.5335 | 0.041687 | 2020 | ASR | 138.5054 | 138.5616 | 138.4985 | 138.5686 | 138.4904 | 138.5767 | 138.4801 | 138.587 | 138.4518 | 138.6152 |
| 32 | 137.5267 | 0.041036 | 2021 | ASR | 137.499 | 137.5543 | 137.4922 | 137.5612 | 137.4842 | 137.5692 | 137.4741 | 137.5793 | 137.4462 | 137.6071 |
| 33 | 136.0989 | 44.39502 | 2022 | ASR | 106.1767 | 166.0212 | 98.76271 | 173.4351 | 90.10568 | 182.0922 | 79.1845 | 193.0133 | 49.08468 | 223.1132 |
| 34 | 134.307 | 62.4265 | 2023 | ASR | 92.2315 | 176.3824 | 81.80628 | 186.8077 | 69.63311 | 198.9808 | 54.27619 | 214.3377 | 11.95102 | 256.6629 |
| 35 | 132.6596 | 75.73223 | 2024 | ASR | 81.6161 | 183.7031 | 68.96881 | 196.3504 | 54.20103 | 211.1182 | 35.5709 | 229.7483 | -15.7756 | 281.0948 |
| 36 | 130.8998 | 86.42179 | 2025 | ASR | 72.65148 | 189.1481 | 58.21904 | 203.5805 | 41.3668 | 220.4327 | 20.10703 | 241.6925 | -38.4869 | 300.2865 |
| 37 | 129.4693 | 95.76553 | 2026 | ASR | 64.92337 | 194.0153 | 48.93052 | 210.0081 | 30.25624 | 228.6824 | 6.697923 | 252.2407 | -58.2311 | 317.1698 |
| 38 | 127.3182 | 105.2946 | 2027 | ASR | 56.34963 | 198.2867 | 38.76543 | 215.8709 | 18.23299 | 236.4034 | -7.66948 | 262.3058 | -79.0592 | 333.6956 |
| 39 | 125.1831 | 113.689 | 2028 | ASR | 48.55672 | 201.8094 | 29.57066 | 220.7955 | 7.401311 | 242.9648 | -20.5662 | 270.9323 | -97.6473 | 348.0134 |
| 40 | 123.3182 | 121.1775 | 2029 | ASR | 41.64454 | 204.9919 | 21.40789 | 225.2285 | -2.22173 | 248.8581 | -32.0314 | 278.6678 | -114.19 | 360.8262 |
| 41 | 121.5096 | 127.7825 | 2030 | ASR | 35.3842 | 207.6351 | 14.04452 | 228.9747 | -10.8731 | 253.8923 | -42.3076 | 285.3268 | -128.944 | 371.9634 |
| 42 | 120.041 | 133.9296 | 2031 | ASR | 29.77244 | 210.3096 | 7.406185 | 232.6759 | -18.7101 | 258.7921 | -51.6568 | 291.7388 | -142.461 | 382.5431 |
| 43 | 117.5463 | 139.7543 | 2032 | ASR | 23.35188 | 211.7407 | 0.012902 | 235.0797 | -27.2392 | 262.3318 | -61.6188 | 296.7114 | -156.372 | 391.4648 |
| 44 | 115.2066 | 145.1607 | 2033 | ASR | 17.36831 | 213.0449 | -6.87352 | 237.2867 | -35.1798 | 265.5931 | -70.8894 | 301.3026 | -169.308 | 399.7215 |
| 45 | 113.2448 | 150.3098 | 2034 | ASR | 11.93601 | 214.5536 | -13.1657 | 239.6554 | -42.4761 | 268.9658 | -79.4523 | 305.942 | -181.362 | 407.852 |
| 46 | 111.5004 | 155.1122 | 2035 | ASR | 6.954806 | 216.0461 | -18.9489 | 241.9498 | -49.1958 | 272.1967 | -87.3534 | 310.3543 | -192.519 | 415.5203 |
| **China** | | | | | | | | | | | | | | |
| 1 | 107.4864 | 0.17582 | 1990 | ASR | 107.3679 | 107.6049 | 107.3385 | 107.6343 | 107.3043 | 107.6686 | 107.261 | 107.7118 | 107.1418 | 107.831 |
| 2 | 106.0597 | 0.170111 | 1991 | ASR | 105.945 | 106.1744 | 105.9166 | 106.2028 | 105.8835 | 106.2359 | 105.8416 | 106.2778 | 105.7263 | 106.3931 |
| 3 | 104.9365 | 0.164915 | 1992 | ASR | 104.8254 | 105.0477 | 104.7978 | 105.0752 | 104.7657 | 105.1074 | 104.7251 | 105.1479 | 104.6133 | 105.2597 |
| 4 | 103.9397 | 0.159869 | 1993 | ASR | 103.8319 | 104.0475 | 103.8053 | 104.0742 | 103.7741 | 104.1053 | 103.7347 | 104.1447 | 103.6264 | 104.253 |
| 5 | 103.2174 | 0.1551 | 1994 | ASR | 103.1129 | 103.322 | 103.087 | 103.3479 | 103.0568 | 103.3781 | 103.0186 | 103.4163 | 102.9134 | 103.5214 |
| 6 | 102.5417 | 0.150062 | 1995 | ASR | 102.4405 | 102.6428 | 102.4155 | 102.6679 | 102.3862 | 102.6971 | 102.3493 | 102.734 | 102.2475 | 102.8358 |
| 7 | 102.4041 | 0.145872 | 1996 | ASR | 102.3058 | 102.5024 | 102.2814 | 102.5267 | 102.2529 | 102.5552 | 102.2171 | 102.5911 | 102.1182 | 102.69 |
| 8 | 103.1434 | 0.142566 | 1997 | ASR | 103.0473 | 103.2395 | 103.0235 | 103.2633 | 102.9957 | 103.2911 | 102.9607 | 103.3262 | 102.864 | 103.4229 |
| 9 | 104.5597 | 0.139928 | 1998 | ASR | 104.4654 | 104.654 | 104.442 | 104.6773 | 104.4147 | 104.7046 | 104.3803 | 104.7391 | 104.2854 | 104.8339 |
| 10 | 106.0801 | 0.137523 | 1999 | ASR | 105.9874 | 106.1728 | 105.9645 | 106.1958 | 105.9377 | 106.2226 | 105.9038 | 106.2564 | 105.8106 | 106.3497 |
| 11 | 108.1606 | 0.136144 | 2000 | ASR | 108.0688 | 108.2523 | 108.0461 | 108.2751 | 108.0195 | 108.3016 | 107.986 | 108.3351 | 107.8937 | 108.4274 |
| 12 | 109.7179 | 0.135206 | 2001 | ASR | 109.6268 | 109.809 | 109.6042 | 109.8316 | 109.5778 | 109.858 | 109.5446 | 109.8912 | 109.4529 | 109.9829 |
| 13 | 109.0782 | 0.133547 | 2002 | ASR | 108.9882 | 109.1682 | 108.9659 | 109.1905 | 108.9399 | 109.2166 | 108.907 | 109.2494 | 108.8165 | 109.34 |
| 14 | 108.6184 | 0.133294 | 2003 | ASR | 108.5286 | 108.7082 | 108.5063 | 108.7305 | 108.4803 | 108.7565 | 108.4475 | 108.7893 | 108.3571 | 108.8797 |
| 15 | 107.7611 | 0.13173 | 2004 | ASR | 107.6723 | 107.8499 | 107.6503 | 107.8719 | 107.6246 | 107.8975 | 107.5922 | 107.93 | 107.5029 | 108.0193 |
| 16 | 105.7217 | 0.128256 | 2005 | ASR | 105.6353 | 105.8082 | 105.6139 | 105.8296 | 105.5888 | 105.8546 | 105.5573 | 105.8861 | 105.4703 | 105.9731 |
| 17 | 101.4323 | 0.121487 | 2006 | ASR | 101.3504 | 101.5142 | 101.3301 | 101.5345 | 101.3065 | 101.5582 | 101.2766 | 101.5881 | 101.1942 | 101.6704 |
| 18 | 99.21234 | 0.11578 | 2007 | ASR | 99.1343 | 99.29038 | 99.11497 | 99.30971 | 99.09239 | 99.33229 | 99.06391 | 99.36077 | 98.98541 | 99.43927 |
| 19 | 98.12383 | 0.111033 | 2008 | ASR | 98.04899 | 98.19867 | 98.03045 | 98.21721 | 98.0088 | 98.23886 | 97.98149 | 98.26617 | 97.90621 | 98.34145 |
| 20 | 97.0717 | 0.106469 | 2009 | ASR | 96.99994 | 97.14346 | 96.98216 | 97.16124 | 96.9614 | 97.182 | 96.93521 | 97.2082 | 96.86302 | 97.28038 |
| 21 | 96.21303 | 0.102211 | 2010 | ASR | 96.14414 | 96.28192 | 96.12707 | 96.29899 | 96.10714 | 96.31892 | 96.082 | 96.34407 | 96.0127 | 96.41337 |
| 22 | 96.24211 | 0.098602 | 2011 | ASR | 96.17565 | 96.30857 | 96.15919 | 96.32504 | 96.13996 | 96.34426 | 96.1157 | 96.36852 | 96.04885 | 96.43537 |
| 23 | 97.03441 | 0.095373 | 2012 | ASR | 96.97013 | 97.09869 | 96.9542 | 97.11462 | 96.9356 | 97.13321 | 96.91214 | 97.15668 | 96.84748 | 97.22134 |
| 24 | 99.25368 | 0.093428 | 2013 | ASR | 99.19071 | 99.31665 | 99.17511 | 99.33226 | 99.15689 | 99.35048 | 99.13391 | 99.37346 | 99.07057 | 99.4368 |
| 25 | 101.5661 | 0.091744 | 2014 | ASR | 101.5042 | 101.6279 | 101.4889 | 101.6432 | 101.471 | 101.6611 | 101.4484 | 101.6837 | 101.3862 | 101.7459 |
| 26 | 103.5261 | 0.089957 | 2015 | ASR | 103.4655 | 103.5867 | 103.4505 | 103.6018 | 103.4329 | 103.6193 | 103.4108 | 103.6414 | 103.3498 | 103.7024 |
| 27 | 105.6642 | 0.088448 | 2016 | ASR | 105.6046 | 105.7238 | 105.5898 | 105.7386 | 105.5726 | 105.7559 | 105.5508 | 105.7776 | 105.4909 | 105.8376 |
| 28 | 107.3438 | 0.08689 | 2017 | ASR | 107.2852 | 107.4023 | 107.2707 | 107.4169 | 107.2538 | 107.4338 | 107.2324 | 107.4552 | 107.1735 | 107.5141 |
| 29 | 108.5498 | 0.085189 | 2018 | ASR | 108.4924 | 108.6073 | 108.4782 | 108.6215 | 108.4616 | 108.6381 | 108.4406 | 108.6591 | 108.3829 | 108.7168 |
| 30 | 110.4757 | 0.083853 | 2019 | ASR | 110.4192 | 110.5322 | 110.4052 | 110.5462 | 110.3888 | 110.5626 | 110.3682 | 110.5832 | 110.3114 | 110.6401 |
| 31 | 114.4865 | 0.083422 | 2020 | ASR | 114.4303 | 114.5427 | 114.4164 | 114.5567 | 114.4001 | 114.5729 | 114.3796 | 114.5935 | 114.323 | 114.65 |
| 32 | 113.0924 | 0.081132 | 2021 | ASR | 113.0378 | 113.1471 | 113.0242 | 113.1607 | 113.0084 | 113.1765 | 112.9884 | 113.1964 | 112.9334 | 113.2515 |
| 33 | 114.1313 | 3.172391 | 2022 | ASR | 111.9931 | 116.2695 | 111.4633 | 116.7993 | 110.8447 | 117.4179 | 110.0643 | 118.1983 | 107.9134 | 120.3492 |
| 34 | 113.883 | 4.041185 | 2023 | ASR | 111.1593 | 116.6068 | 110.4844 | 117.2817 | 109.6964 | 118.0697 | 108.7022 | 119.0638 | 105.9623 | 121.8038 |
| 35 | 113.6284 | 4.786882 | 2024 | ASR | 110.4021 | 116.8548 | 109.6027 | 117.6542 | 108.6692 | 118.5876 | 107.4917 | 119.7652 | 104.2462 | 123.0107 |
| 36 | 113.3715 | 5.459895 | 2025 | ASR | 109.6915 | 117.0515 | 108.7797 | 117.9633 | 107.715 | 119.0279 | 106.3719 | 120.3711 | 102.6701 | 124.0729 |
| 37 | 113.1072 | 6.084282 | 2026 | ASR | 109.0064 | 117.208 | 107.9904 | 118.2241 | 106.8039 | 119.4106 | 105.3072 | 120.9073 | 101.182 | 125.0324 |
| 38 | 112.8337 | 6.673803 | 2027 | ASR | 108.3356 | 117.3319 | 107.2211 | 118.4464 | 105.9197 | 119.7478 | 104.2779 | 121.3895 | 99.75307 | 125.9144 |
| 39 | 112.5531 | 7.236075 | 2028 | ASR | 107.676 | 117.4302 | 106.4676 | 118.6387 | 105.0566 | 120.0497 | 103.2765 | 121.8298 | 98.37042 | 126.7358 |
| 40 | 112.2724 | 7.77701 | 2029 | ASR | 107.0307 | 117.5141 | 105.7319 | 118.8128 | 104.2154 | 120.3293 | 102.3022 | 122.2425 | 97.02943 | 127.5153 |
| 41 | 111.9942 | 8.301128 | 2030 | ASR | 106.3992 | 117.5891 | 105.0129 | 118.9754 | 103.3942 | 120.5941 | 101.3521 | 122.6362 | 95.72395 | 128.2644 |
| 42 | 111.7107 | 8.811338 | 2031 | ASR | 105.7718 | 117.6495 | 104.3003 | 119.121 | 102.5821 | 120.8392 | 100.4145 | 123.0068 | 94.44046 | 128.9809 |
| 43 | 111.4179 | 9.310112 | 2032 | ASR | 105.1429 | 117.6929 | 103.5881 | 119.2477 | 101.7726 | 121.0632 | 99.48232 | 123.3534 | 93.17006 | 129.6657 |
| 44 | 111.1209 | 9.799142 | 2033 | ASR | 104.5163 | 117.7255 | 102.8798 | 119.362 | 100.969 | 121.2728 | 98.55839 | 123.6834 | 91.91457 | 130.3272 |
| 45 | 110.8269 | 10.28052 | 2034 | ASR | 103.8978 | 117.756 | 102.181 | 119.4728 | 100.1763 | 121.4775 | 97.64729 | 124.0065 | 90.6771 | 130.9767 |
| 46 | 110.5381 | 10.75606 | 2035 | ASR | 103.2885 | 117.7877 | 101.4923 | 119.584 | 99.39486 | 121.6814 | 96.74887 | 124.3274 | 89.45626 | 131.62 |
| **Brunei Darussalam** | | | | | | | | | | | | | | |
| 1 | 114.6157 | 4.743408 | 1990 | ASR | 111.4186 | 117.8127 | 110.6265 | 118.6049 | 109.7015 | 119.5298 | 108.5346 | 120.6967 | 105.3186 | 123.9128 |
| 2 | 115.0623 | 4.398169 | 1991 | ASR | 112.098 | 118.0267 | 111.3635 | 118.7612 | 110.5058 | 119.6189 | 109.4239 | 120.7008 | 106.4419 | 123.6828 |
| 3 | 115.517 | 4.100897 | 1992 | ASR | 112.7529 | 118.281 | 112.0681 | 118.9658 | 111.2684 | 119.7655 | 110.2596 | 120.7743 | 107.4792 | 123.5547 |
| 4 | 115.8991 | 3.842935 | 1993 | ASR | 113.309 | 118.4892 | 112.6672 | 119.131 | 111.9178 | 119.8804 | 110.9725 | 120.8257 | 108.367 | 123.4313 |
| 5 | 116.2038 | 3.621218 | 1994 | ASR | 113.7631 | 118.6445 | 113.1583 | 119.2492 | 112.4522 | 119.9554 | 111.5614 | 120.8462 | 109.1062 | 123.3014 |
| 6 | 116.4774 | 3.431629 | 1995 | ASR | 114.1644 | 118.7903 | 113.5914 | 119.3634 | 112.9222 | 120.0325 | 112.078 | 120.8767 | 109.7514 | 123.2034 |
| 7 | 116.7242 | 3.271188 | 1996 | ASR | 114.5194 | 118.929 | 113.9731 | 119.4753 | 113.3352 | 120.1131 | 112.5305 | 120.9179 | 110.3127 | 123.1357 |
| 8 | 116.9437 | 3.136662 | 1997 | ASR | 114.8295 | 119.0578 | 114.3057 | 119.5816 | 113.6941 | 120.1932 | 112.9225 | 120.9649 | 110.7958 | 123.0915 |
| 9 | 117.1547 | 3.024862 | 1998 | ASR | 115.1159 | 119.1935 | 114.6108 | 119.6986 | 114.0209 | 120.2885 | 113.2768 | 121.0326 | 111.226 | 123.0834 |
| 10 | 117.3094 | 2.93127 | 1999 | ASR | 115.3338 | 119.2851 | 114.8442 | 119.7746 | 114.2726 | 120.3462 | 113.5515 | 121.0673 | 111.5641 | 123.0547 |
| 11 | 117.4145 | 2.852917 | 2000 | ASR | 115.4916 | 119.3374 | 115.0152 | 119.8138 | 114.4589 | 120.3701 | 113.757 | 121.0719 | 111.8228 | 123.0062 |
| 12 | 117.537 | 2.789242 | 2001 | ASR | 115.6571 | 119.417 | 115.1913 | 119.8828 | 114.6474 | 120.4267 | 113.9612 | 121.1129 | 112.0701 | 123.004 |
| 13 | 117.6243 | 2.737107 | 2002 | ASR | 115.7795 | 119.4691 | 115.3224 | 119.9262 | 114.7887 | 120.46 | 114.1154 | 121.1333 | 112.2596 | 122.9891 |
| 14 | 117.675 | 2.694396 | 2003 | ASR | 115.8589 | 119.491 | 115.409 | 119.9409 | 114.8836 | 120.4664 | 114.2207 | 121.1292 | 112.3939 | 122.956 |
| 15 | 117.7454 | 2.661428 | 2004 | ASR | 115.9516 | 119.5392 | 115.5071 | 119.9836 | 114.9881 | 120.5026 | 114.3334 | 121.1573 | 112.529 | 122.9618 |
| 16 | 117.7423 | 2.634089 | 2005 | ASR | 115.967 | 119.5177 | 115.5271 | 119.9576 | 115.0134 | 120.4713 | 114.3654 | 121.1192 | 112.5795 | 122.9052 |
| 17 | 117.7129 | 2.613259 | 2006 | ASR | 115.9516 | 119.4743 | 115.5152 | 119.9107 | 115.0056 | 120.4203 | 114.3627 | 121.0631 | 112.5909 | 122.8349 |
| 18 | 117.7093 | 2.599212 | 2007 | ASR | 115.9575 | 119.4612 | 115.5234 | 119.8953 | 115.0166 | 120.4021 | 114.3772 | 121.0415 | 112.6149 | 122.8038 |
| 19 | 117.7008 | 2.590345 | 2008 | ASR | 115.955 | 119.4467 | 115.5224 | 119.8793 | 115.0172 | 120.3844 | 114.38 | 121.0217 | 112.6238 | 122.7779 |
| 20 | 117.6494 | 2.586853 | 2009 | ASR | 115.9059 | 119.393 | 115.4739 | 119.825 | 114.9694 | 120.3294 | 114.3331 | 120.9658 | 112.5792 | 122.7197 |
| 21 | 117.5261 | 2.587179 | 2010 | ASR | 115.7823 | 119.2698 | 115.3503 | 119.7019 | 114.8458 | 120.2064 | 114.2093 | 120.8428 | 112.4552 | 122.5969 |
| 22 | 117.3774 | 2.593422 | 2011 | ASR | 115.6295 | 119.1254 | 115.1964 | 119.5585 | 114.6907 | 120.0642 | 114.0527 | 120.7022 | 112.2943 | 122.4606 |
| 23 | 117.1039 | 2.60322 | 2012 | ASR | 115.3493 | 118.8585 | 114.9146 | 119.2932 | 114.407 | 119.8008 | 113.7666 | 120.4412 | 112.0016 | 122.2062 |
| 24 | 116.8237 | 2.619162 | 2013 | ASR | 115.0584 | 118.589 | 114.621 | 119.0264 | 114.1102 | 119.5371 | 113.4659 | 120.1814 | 111.6901 | 121.9572 |
| 25 | 116.5124 | 2.642245 | 2014 | ASR | 114.7315 | 118.2933 | 114.2903 | 118.7345 | 113.775 | 119.2498 | 113.1251 | 119.8998 | 111.3336 | 121.6912 |
| 26 | 116.1622 | 2.671485 | 2015 | ASR | 114.3616 | 117.9628 | 113.9155 | 118.4089 | 113.3945 | 118.9299 | 112.7374 | 119.587 | 110.9261 | 121.3983 |
| 27 | 115.8335 | 2.711675 | 2016 | ASR | 114.0058 | 117.6611 | 113.5529 | 118.114 | 113.0242 | 118.6428 | 112.3571 | 119.3098 | 110.5186 | 121.1484 |
| 28 | 115.4305 | 2.76188 | 2017 | ASR | 113.5689 | 117.292 | 113.1077 | 117.7532 | 112.5691 | 118.2918 | 111.8897 | 118.9712 | 110.0172 | 120.8437 |
| 29 | 114.9423 | 2.824565 | 2018 | ASR | 113.0386 | 116.8461 | 112.5669 | 117.3178 | 112.0161 | 117.8686 | 111.3212 | 118.5634 | 109.4062 | 120.4785 |
| 30 | 114.4098 | 2.906122 | 2019 | ASR | 112.451 | 116.3685 | 111.9657 | 116.8538 | 111.399 | 117.4205 | 110.6841 | 118.1354 | 108.7138 | 120.1058 |
| 31 | 113.7809 | 3.013187 | 2020 | ASR | 111.75 | 115.8118 | 111.2468 | 116.315 | 110.6592 | 116.9025 | 109.918 | 117.6438 | 107.875 | 119.6867 |
| 32 | 113.2237 | 3.162885 | 2021 | ASR | 111.0919 | 115.3555 | 110.5637 | 115.8837 | 109.947 | 116.5004 | 109.1689 | 117.2785 | 107.0244 | 119.423 |
| 33 | 112.7847 | 3.490887 | 2022 | ASR | 110.4319 | 115.1376 | 109.8489 | 115.7206 | 109.1682 | 116.4013 | 108.3094 | 117.2601 | 105.9426 | 119.6269 |
| 34 | 112.2598 | 3.713878 | 2023 | ASR | 109.7566 | 114.7629 | 109.1364 | 115.3832 | 108.4122 | 116.1074 | 107.4986 | 117.021 | 104.9806 | 119.539 |
| 35 | 111.7252 | 3.938745 | 2024 | ASR | 109.0705 | 114.3799 | 108.4127 | 115.0377 | 107.6446 | 115.8057 | 106.6757 | 116.7747 | 104.0052 | 119.4451 |
| 36 | 111.1837 | 4.166805 | 2025 | ASR | 108.3753 | 113.9922 | 107.6795 | 114.688 | 106.8669 | 115.5005 | 105.8419 | 116.5256 | 103.0168 | 119.3507 |
| 37 | 110.6387 | 4.399483 | 2026 | ASR | 107.6735 | 113.604 | 106.9388 | 114.3387 | 106.0809 | 115.1966 | 104.9986 | 116.2789 | 102.0157 | 119.2617 |
| 38 | 110.0919 | 4.637985 | 2027 | ASR | 106.9659 | 113.2179 | 106.1913 | 113.9924 | 105.2869 | 114.8968 | 104.146 | 116.0378 | 101.0014 | 119.1823 |
| 39 | 109.5445 | 4.882915 | 2028 | ASR | 106.2534 | 112.8356 | 105.4379 | 113.651 | 104.4858 | 114.6032 | 103.2846 | 115.8044 | 99.97396 | 119.115 |
| 40 | 108.9982 | 5.135631 | 2029 | ASR | 105.5368 | 112.4596 | 104.6792 | 113.3173 | 103.6777 | 114.3187 | 102.4143 | 115.5821 | 98.93238 | 119.0641 |
| 41 | 108.4549 | 5.397919 | 2030 | ASR | 104.8167 | 112.0931 | 103.9153 | 112.9946 | 102.8627 | 114.0472 | 101.5348 | 115.3751 | 97.87502 | 119.0349 |
| 42 | 107.9163 | 5.671641 | 2031 | ASR | 104.0936 | 111.7389 | 103.1464 | 112.6861 | 102.0404 | 113.7921 | 100.6452 | 115.1873 | 96.79985 | 119.0327 |
| 43 | 107.3825 | 5.958455 | 2032 | ASR | 103.3665 | 111.3985 | 102.3714 | 112.3935 | 101.2095 | 113.5554 | 99.74374 | 115.0212 | 95.70391 | 119.0611 |
| 44 | 106.8536 | 6.259455 | 2033 | ASR | 102.6347 | 111.0725 | 101.5894 | 112.1178 | 100.3688 | 113.3384 | 98.82898 | 114.8782 | 94.58507 | 119.1221 |
| 45 | 106.3301 | 6.57638 | 2034 | ASR | 101.8976 | 110.7626 | 100.7993 | 111.8608 | 99.51694 | 113.1432 | 97.89915 | 114.761 | 93.44037 | 119.2198 |
| 46 | 105.8124 | 6.91131 | 2035 | ASR | 101.1542 | 110.4706 | 99.99999 | 111.6248 | 98.65229 | 112.9725 | 96.95211 | 114.6727 | 92.26624 | 119.3586 |
| **Cambodia** | | | | | | | | | | | | | | |
| 1 | 180.0811 | 1.825839 | 1990 | ASR | 178.8505 | 181.3117 | 178.5456 | 181.6166 | 178.1895 | 181.9727 | 177.7404 | 182.4218 | 176.5025 | 183.6598 |
| 2 | 181.2992 | 1.615294 | 1991 | ASR | 180.2105 | 182.3879 | 179.9407 | 182.6576 | 179.6257 | 182.9726 | 179.2284 | 183.37 | 178.1332 | 184.4652 |
| 3 | 182.2912 | 1.535454 | 1992 | ASR | 181.2563 | 183.3261 | 180.9999 | 183.5825 | 180.7005 | 183.8819 | 180.3227 | 184.2596 | 179.2817 | 185.3007 |
| 4 | 183.2609 | 1.498789 | 1993 | ASR | 182.2507 | 184.2711 | 182.0004 | 184.5214 | 181.7082 | 184.8137 | 181.3395 | 185.1824 | 180.3233 | 186.1985 |
| 5 | 184.2807 | 1.477964 | 1994 | ASR | 183.2845 | 185.2768 | 183.0377 | 185.5236 | 182.7495 | 185.8118 | 182.3859 | 186.1754 | 181.3839 | 187.1775 |
| 6 | 185.1147 | 1.462381 | 1995 | ASR | 184.129 | 186.1003 | 183.8848 | 186.3445 | 183.5997 | 186.6297 | 183.2399 | 186.9895 | 182.2484 | 187.9809 |
| 7 | 185.7207 | 1.448591 | 1996 | ASR | 184.7443 | 186.697 | 184.5024 | 186.9389 | 184.2199 | 187.2214 | 183.8636 | 187.5778 | 182.8814 | 188.5599 |
| 8 | 186.1441 | 1.435419 | 1997 | ASR | 185.1766 | 187.1115 | 184.9369 | 187.3513 | 184.657 | 187.6312 | 184.3039 | 187.9843 | 183.3307 | 188.9575 |
| 9 | 186.5009 | 1.422713 | 1998 | ASR | 185.542 | 187.4598 | 185.3044 | 187.6974 | 185.027 | 187.9748 | 184.677 | 188.3248 | 183.7124 | 189.2894 |
| 10 | 186.8071 | 1.410418 | 1999 | ASR | 185.8564 | 187.7577 | 185.6209 | 187.9932 | 185.3459 | 188.2682 | 184.9989 | 188.6152 | 184.0426 | 189.5715 |
| 11 | 187.004 | 1.397892 | 2000 | ASR | 186.0618 | 187.9462 | 185.8284 | 188.1796 | 185.5558 | 188.4522 | 185.2119 | 188.7961 | 184.2641 | 189.7439 |
| 12 | 187.239 | 1.385606 | 2001 | ASR | 186.3051 | 188.1729 | 186.0737 | 188.4043 | 185.8035 | 188.6745 | 185.4627 | 189.0154 | 184.5232 | 189.9548 |
| 13 | 187.5457 | 1.373536 | 2002 | ASR | 186.6199 | 188.4714 | 186.3905 | 188.7008 | 186.1227 | 188.9687 | 185.7848 | 189.3065 | 184.8535 | 190.2378 |
| 14 | 187.8907 | 1.36139 | 2003 | ASR | 186.9731 | 188.8082 | 186.7457 | 189.0356 | 186.4803 | 189.3011 | 186.1454 | 189.636 | 185.2223 | 190.559 |
| 15 | 188.5909 | 1.350633 | 2004 | ASR | 187.6805 | 189.5012 | 187.455 | 189.7267 | 187.1916 | 189.9901 | 186.8593 | 190.3224 | 185.9436 | 191.2381 |
| 16 | 189.5686 | 1.340862 | 2005 | ASR | 188.6649 | 190.4724 | 188.441 | 190.6963 | 188.1795 | 190.9578 | 187.8497 | 191.2876 | 186.9406 | 192.1967 |
| 17 | 190.8306 | 1.332268 | 2006 | ASR | 189.9327 | 191.7286 | 189.7102 | 191.9511 | 189.4504 | 192.2109 | 189.1227 | 192.5386 | 188.2194 | 193.4419 |
| 18 | 192.6903 | 1.32581 | 2007 | ASR | 191.7967 | 193.5839 | 191.5753 | 193.8053 | 191.3168 | 194.0638 | 190.9906 | 194.39 | 190.0917 | 195.2889 |
| 19 | 194.5845 | 1.319391 | 2008 | ASR | 193.6953 | 195.4738 | 193.4749 | 195.6942 | 193.2177 | 195.9514 | 192.8931 | 196.276 | 191.9985 | 197.1706 |
| 20 | 196.6247 | 1.314402 | 2009 | ASR | 195.7388 | 197.5106 | 195.5193 | 197.7301 | 195.263 | 197.9864 | 194.9397 | 198.3098 | 194.0485 | 199.2009 |
| 21 | 198.6804 | 1.309764 | 2010 | ASR | 197.7976 | 199.5632 | 197.5789 | 199.7819 | 197.3235 | 200.0373 | 197.0013 | 200.3595 | 196.1132 | 201.2475 |
| 22 | 200.8795 | 1.306071 | 2011 | ASR | 199.9992 | 201.7598 | 199.7811 | 201.9779 | 199.5264 | 202.2326 | 199.2051 | 202.5539 | 198.3196 | 203.4394 |
| 23 | 203.1715 | 1.302984 | 2012 | ASR | 202.2933 | 204.0498 | 202.0757 | 204.2674 | 201.8217 | 204.5214 | 201.5011 | 204.842 | 200.6177 | 205.7254 |
| 24 | 205.6039 | 1.300071 | 2013 | ASR | 204.7277 | 206.4802 | 204.5106 | 206.6973 | 204.2571 | 206.9508 | 203.9372 | 207.2706 | 203.0558 | 208.1521 |
| 25 | 208.1702 | 1.298483 | 2014 | ASR | 207.295 | 209.0454 | 207.0782 | 209.2622 | 206.825 | 209.5154 | 206.5055 | 209.8348 | 205.6252 | 210.7152 |
| 26 | 210.413 | 1.295417 | 2015 | ASR | 209.5399 | 211.2861 | 209.3235 | 211.5024 | 209.0709 | 211.755 | 208.7523 | 212.0737 | 207.874 | 212.952 |
| 27 | 212.5506 | 1.292037 | 2016 | ASR | 211.6798 | 213.4214 | 211.464 | 213.6372 | 211.2121 | 213.8892 | 210.8942 | 214.207 | 210.0182 | 215.083 |
| 28 | 214.6497 | 1.288831 | 2017 | ASR | 213.7811 | 215.5184 | 213.5658 | 215.7336 | 213.3145 | 215.985 | 212.9975 | 216.302 | 212.1236 | 217.1758 |
| 29 | 216.2059 | 1.282368 | 2018 | ASR | 215.3415 | 217.0702 | 215.1274 | 217.2843 | 214.8773 | 217.5344 | 214.5619 | 217.8499 | 213.6924 | 218.7193 |
| 30 | 217.3581 | 1.276428 | 2019 | ASR | 216.4978 | 218.2184 | 216.2846 | 218.4316 | 216.0357 | 218.6805 | 215.7217 | 218.9945 | 214.8563 | 219.8599 |
| 31 | 218.5337 | 1.282194 | 2020 | ASR | 217.6695 | 219.3979 | 217.4554 | 219.612 | 217.2054 | 219.8621 | 216.8899 | 220.1775 | 216.0206 | 221.0468 |
| 32 | 219.351 | 1.368882 | 2021 | ASR | 218.4284 | 220.2736 | 218.1998 | 220.5022 | 217.9329 | 220.7692 | 217.5961 | 221.1059 | 216.668 | 222.034 |
| 33 | 219.2799 | 2.773855 | 2022 | ASR | 217.4103 | 221.1495 | 216.9471 | 221.6127 | 216.4062 | 222.1536 | 215.7238 | 222.836 | 213.8432 | 224.7167 |
| 34 | 219.6615 | 3.507755 | 2023 | ASR | 217.2972 | 222.0257 | 216.7114 | 222.6115 | 216.0274 | 223.2955 | 215.1645 | 224.1584 | 212.7863 | 226.5367 |
| 35 | 220.0127 | 4.148095 | 2024 | ASR | 217.2169 | 222.8085 | 216.5241 | 223.5012 | 215.7153 | 224.3101 | 214.6948 | 225.3305 | 211.8824 | 228.1429 |
| 36 | 220.3316 | 4.733845 | 2025 | ASR | 217.1409 | 223.5222 | 216.3504 | 224.3127 | 215.4273 | 225.2358 | 214.2628 | 226.4003 | 211.0532 | 229.6099 |
| 37 | 220.6221 | 5.285269 | 2026 | ASR | 217.0598 | 224.1844 | 216.1772 | 225.067 | 215.1466 | 226.0976 | 213.8464 | 227.3978 | 210.263 | 230.9812 |
| 38 | 220.8877 | 5.814213 | 2027 | ASR | 216.969 | 224.8065 | 215.998 | 225.7775 | 214.8642 | 226.9113 | 213.4339 | 228.3416 | 209.4919 | 232.2836 |
| 39 | 221.1341 | 6.324415 | 2028 | ASR | 216.8715 | 225.3968 | 215.8153 | 226.453 | 214.582 | 227.6862 | 213.0262 | 229.242 | 208.7383 | 233.53 |
| 40 | 221.3672 | 6.819983 | 2029 | ASR | 216.7705 | 225.9638 | 215.6316 | 227.1028 | 214.3017 | 228.4327 | 212.624 | 230.1104 | 208 | 234.7343 |
| 41 | 221.589 | 7.305194 | 2030 | ASR | 216.6653 | 226.5127 | 215.4453 | 227.7327 | 214.0208 | 229.1572 | 212.2237 | 230.9543 | 207.2708 | 235.9072 |
| 42 | 221.8053 | 7.784393 | 2031 | ASR | 216.5586 | 227.052 | 215.2587 | 228.352 | 213.7407 | 229.87 | 211.8257 | 231.7849 | 206.5479 | 237.0627 |
| 43 | 222.017 | 8.261102 | 2032 | ASR | 216.449 | 227.585 | 215.0694 | 228.9646 | 213.4585 | 230.5755 | 211.4262 | 232.6077 | 205.8252 | 238.2087 |
| 44 | 222.2222 | 8.734943 | 2033 | ASR | 216.3348 | 228.1095 | 214.8761 | 229.5682 | 213.1728 | 231.2716 | 211.024 | 233.4204 | 205.1017 | 239.3426 |
| 45 | 222.4259 | 9.207312 | 2034 | ASR | 216.2202 | 228.6317 | 214.6826 | 230.1693 | 212.8872 | 231.9647 | 210.6222 | 234.2297 | 204.3796 | 240.4723 |
| 46 | 222.6331 | 9.680733 | 2035 | ASR | 216.1083 | 229.1579 | 214.4916 | 230.7746 | 212.6039 | 232.6623 | 210.2224 | 235.0438 | 203.6589 | 241.6073 |
| **Indonesia** | | | | | | | | | | | | | | |
| 1 | 129.4738 | 0.428307 | 1990 | ASR | 129.1851 | 129.7624 | 129.1136 | 129.834 | 129.03 | 129.9175 | 128.9247 | 130.0228 | 128.6343 | 130.3132 |
| 2 | 131.242 | 0.416271 | 1991 | ASR | 130.9615 | 131.5226 | 130.892 | 131.5921 | 130.8108 | 131.6733 | 130.7084 | 131.7757 | 130.4261 | 132.0579 |
| 3 | 132.7979 | 0.411262 | 1992 | ASR | 132.5207 | 133.0751 | 132.452 | 133.1437 | 132.3718 | 133.2239 | 132.2706 | 133.3251 | 131.9918 | 133.6039 |
| 4 | 134.2814 | 0.407047 | 1993 | ASR | 134.007 | 134.5557 | 133.939 | 134.6237 | 133.8597 | 134.7031 | 133.7595 | 134.8032 | 133.4835 | 135.0792 |
| 5 | 135.2484 | 0.402504 | 1994 | ASR | 134.9771 | 135.5197 | 134.9099 | 135.5869 | 134.8314 | 135.6654 | 134.7324 | 135.7644 | 134.4595 | 136.0373 |
| 6 | 135.7688 | 0.397575 | 1995 | ASR | 135.5009 | 136.0368 | 135.4345 | 136.1032 | 135.3569 | 136.1807 | 135.2591 | 136.2785 | 134.9896 | 136.5481 |
| 7 | 136.2255 | 0.393524 | 1996 | ASR | 135.9603 | 136.4907 | 135.8946 | 136.5565 | 135.8178 | 136.6332 | 135.721 | 136.73 | 135.4542 | 136.9968 |
| 8 | 136.6709 | 0.388627 | 1997 | ASR | 136.409 | 136.9329 | 136.3441 | 136.9978 | 136.2683 | 137.0735 | 136.1727 | 137.1691 | 135.9092 | 137.4326 |
| 9 | 137.1711 | 0.384044 | 1998 | ASR | 136.9123 | 137.43 | 136.8481 | 137.4941 | 136.7732 | 137.569 | 136.6788 | 137.6635 | 136.4184 | 137.9238 |
| 10 | 137.7449 | 0.379928 | 1999 | ASR | 137.4888 | 138.001 | 137.4254 | 138.0644 | 137.3513 | 138.1385 | 137.2578 | 138.232 | 137.0002 | 138.4896 |
| 11 | 138.1913 | 0.37602 | 2000 | ASR | 137.9379 | 138.4448 | 137.8751 | 138.5076 | 137.8018 | 138.5809 | 137.7093 | 138.6734 | 137.4543 | 138.9283 |
| 12 | 138.3535 | 0.372318 | 2001 | ASR | 138.1025 | 138.6044 | 138.0404 | 138.6666 | 137.9678 | 138.7392 | 137.8762 | 138.8308 | 137.6237 | 139.0832 |
| 13 | 136.8329 | 0.365553 | 2002 | ASR | 136.5866 | 137.0793 | 136.5255 | 137.1404 | 136.4542 | 137.2116 | 136.3643 | 137.3016 | 136.1165 | 137.5494 |
| 14 | 135.4811 | 0.359411 | 2003 | ASR | 135.2388 | 135.7233 | 135.1788 | 135.7833 | 135.1087 | 135.8534 | 135.0203 | 135.9418 | 134.7766 | 136.1855 |
| 15 | 134.4778 | 0.354322 | 2004 | ASR | 134.239 | 134.7166 | 134.1798 | 134.7758 | 134.1108 | 134.8449 | 134.0236 | 134.9321 | 133.7834 | 135.1723 |
| 16 | 133.6566 | 0.349883 | 2005 | ASR | 133.4208 | 133.8924 | 133.3623 | 133.9508 | 133.2941 | 134.0191 | 133.208 | 134.1051 | 132.9708 | 134.3424 |
| 17 | 133.1037 | 0.346186 | 2006 | ASR | 132.8703 | 133.337 | 132.8125 | 133.3948 | 132.745 | 133.4623 | 132.6599 | 133.5475 | 132.4251 | 133.7822 |
| 18 | 132.1475 | 0.341636 | 2007 | ASR | 131.9173 | 132.3778 | 131.8602 | 132.4348 | 131.7936 | 132.5014 | 131.7095 | 132.5855 | 131.4779 | 132.8171 |
| 19 | 126.3198 | 0.330093 | 2008 | ASR | 126.0973 | 126.5423 | 126.0422 | 126.5974 | 125.9778 | 126.6617 | 125.8966 | 126.7429 | 125.6728 | 126.9668 |
| 20 | 122.2155 | 0.321377 | 2009 | ASR | 121.9989 | 122.4321 | 121.9452 | 122.4858 | 121.8826 | 122.5485 | 121.8035 | 122.6275 | 121.5856 | 122.8454 |
| 21 | 119.0259 | 0.314178 | 2010 | ASR | 118.8142 | 119.2377 | 118.7617 | 119.2902 | 118.7005 | 119.3514 | 118.6232 | 119.4287 | 118.4102 | 119.6417 |
| 22 | 116.4204 | 0.308114 | 2011 | ASR | 116.2127 | 116.628 | 116.1613 | 116.6795 | 116.1012 | 116.7396 | 116.0254 | 116.8154 | 115.8165 | 117.0243 |
| 23 | 117.0805 | 0.306261 | 2012 | ASR | 116.8741 | 117.287 | 116.823 | 117.3381 | 116.7632 | 117.3978 | 116.6879 | 117.4732 | 116.4803 | 117.6808 |
| 24 | 116.8363 | 0.303256 | 2013 | ASR | 116.6319 | 117.0407 | 116.5812 | 117.0913 | 116.5221 | 117.1505 | 116.4475 | 117.2251 | 116.2419 | 117.4307 |
| 25 | 117.0484 | 0.300937 | 2014 | ASR | 116.8456 | 117.2512 | 116.7953 | 117.3015 | 116.7366 | 117.3602 | 116.6626 | 117.4342 | 116.4586 | 117.6382 |
| 26 | 116.251 | 0.297325 | 2015 | ASR | 116.0506 | 116.4514 | 116.0009 | 116.501 | 115.943 | 116.559 | 115.8698 | 116.6322 | 115.6682 | 116.8337 |
| 27 | 115.8348 | 0.293519 | 2016 | ASR | 115.6369 | 116.0326 | 115.5879 | 116.0816 | 115.5307 | 116.1389 | 115.4585 | 116.2111 | 115.2595 | 116.4101 |
| 28 | 115.62 | 0.289891 | 2017 | ASR | 115.4246 | 115.8153 | 115.3762 | 115.8638 | 115.3196 | 115.9203 | 115.2483 | 115.9916 | 115.0518 | 116.1881 |
| 29 | 115.4032 | 0.286368 | 2018 | ASR | 115.2102 | 115.5963 | 115.1624 | 115.6441 | 115.1066 | 115.6999 | 115.0361 | 115.7704 | 114.842 | 115.9645 |
| 30 | 115.2237 | 0.282982 | 2019 | ASR | 115.033 | 115.4145 | 114.9858 | 115.4617 | 114.9306 | 115.5169 | 114.861 | 115.5865 | 114.6691 | 115.7784 |
| 31 | 115.2641 | 0.280596 | 2020 | ASR | 115.075 | 115.4532 | 115.0281 | 115.5001 | 114.9734 | 115.5548 | 114.9044 | 115.6238 | 114.7141 | 115.8141 |
| 32 | 114.7818 | 0.280584 | 2021 | ASR | 114.5927 | 114.9709 | 114.5458 | 115.0178 | 114.4911 | 115.0725 | 114.4221 | 115.1415 | 114.2318 | 115.3317 |
| 33 | 112.5816 | 1.686119 | 2022 | ASR | 111.4452 | 113.7181 | 111.1636 | 113.9996 | 110.8348 | 114.3284 | 110.42 | 114.7432 | 109.2768 | 115.8864 |
| 34 | 111.3951 | 2.238266 | 2023 | ASR | 109.8865 | 112.9037 | 109.5127 | 113.2775 | 109.0763 | 113.714 | 108.5257 | 114.2646 | 107.0081 | 115.7821 |
| 35 | 110.1942 | 2.684493 | 2024 | ASR | 108.3849 | 112.0036 | 107.9365 | 112.4519 | 107.4131 | 112.9753 | 106.7527 | 113.6357 | 104.9326 | 115.4558 |
| 36 | 108.9797 | 3.070101 | 2025 | ASR | 106.9104 | 111.0489 | 106.3977 | 111.5616 | 105.7991 | 112.1603 | 105.0438 | 112.9156 | 102.9623 | 114.9971 |
| 37 | 107.7658 | 3.415478 | 2026 | ASR | 105.4638 | 110.0679 | 104.8934 | 110.6382 | 104.2274 | 111.3043 | 103.3872 | 112.1445 | 101.0715 | 114.4602 |
| 38 | 106.5531 | 3.731831 | 2027 | ASR | 104.0378 | 109.0683 | 103.4146 | 109.6916 | 102.6869 | 110.4193 | 101.7689 | 111.3373 | 99.2387 | 113.8675 |
| 39 | 105.3378 | 4.024375 | 2028 | ASR | 102.6254 | 108.0502 | 101.9533 | 108.7223 | 101.1686 | 109.5071 | 100.1786 | 110.4971 | 97.45004 | 113.2256 |
| 40 | 104.1236 | 4.297141 | 2029 | ASR | 101.2273 | 107.0198 | 100.5097 | 107.7374 | 99.67171 | 108.5754 | 98.61462 | 109.6325 | 95.70116 | 112.546 |
| 41 | 102.9145 | 4.553414 | 2030 | ASR | 99.84552 | 105.9835 | 99.0851 | 106.7439 | 98.19718 | 107.6319 | 97.07704 | 108.752 | 93.98983 | 111.8392 |
| 42 | 101.7264 | 4.796531 | 2031 | ASR | 98.49357 | 104.9593 | 97.69255 | 105.7603 | 96.75723 | 106.6956 | 95.57728 | 107.8756 | 92.32523 | 111.1276 |
| 43 | 100.5579 | 5.028843 | 2032 | ASR | 97.16847 | 103.9474 | 96.32865 | 104.7872 | 95.34803 | 105.7678 | 94.11093 | 107.0049 | 90.70138 | 110.4144 |
| 44 | 99.3992 | 5.250625 | 2033 | ASR | 95.86028 | 102.9381 | 94.98342 | 103.815 | 93.95955 | 104.8388 | 92.6679 | 106.1305 | 89.10797 | 109.6904 |
| 45 | 98.25305 | 5.462932 | 2034 | ASR | 94.57104 | 101.9351 | 93.65873 | 102.8474 | 92.59346 | 103.9127 | 91.24958 | 105.2565 | 87.54571 | 108.9604 |
| 46 | 97.12571 | 5.667205 | 2035 | ASR | 93.30602 | 100.9454 | 92.35959 | 101.8918 | 91.25449 | 102.9969 | 89.86036 | 104.3911 | 86.01799 | 108.2334 |
| **Lao People's Democratic Republic** | | | | | | | | | | | | | | |
| 1 | 89.14087 | 1.438671 | 1990 | ASR | 88.1712 | 90.11053 | 87.93095 | 90.35079 | 87.65041 | 90.63133 | 87.29649 | 90.98525 | 86.32107 | 91.96067 |
| 2 | 89.63953 | 1.299266 | 1991 | ASR | 88.76383 | 90.51524 | 88.54685 | 90.73221 | 88.29349 | 90.98557 | 87.97387 | 91.30519 | 87.09297 | 92.18609 |
| 3 | 90.08758 | 1.194429 | 1992 | ASR | 89.28254 | 90.89263 | 89.08307 | 91.0921 | 88.85015 | 91.32501 | 88.55632 | 91.61884 | 87.7465 | 92.42866 |
| 4 | 90.48938 | 1.115985 | 1993 | ASR | 89.73721 | 91.24156 | 89.55084 | 91.42793 | 89.33322 | 91.64555 | 89.05869 | 91.92008 | 88.30205 | 92.67672 |
| 5 | 90.83197 | 1.057385 | 1994 | ASR | 90.11929 | 91.54464 | 89.94271 | 91.72123 | 89.73652 | 91.92742 | 89.4764 | 92.18753 | 88.75949 | 92.90444 |
| 6 | 91.14545 | 1.014093 | 1995 | ASR | 90.46195 | 91.82895 | 90.2926 | 91.99831 | 90.09485 | 92.19605 | 89.84539 | 92.44552 | 89.15783 | 93.13308 |
| 7 | 91.42577 | 0.982928 | 1996 | ASR | 90.76328 | 92.08827 | 90.59913 | 92.25241 | 90.40746 | 92.44409 | 90.16566 | 92.68589 | 89.49923 | 93.35231 |
| 8 | 91.71379 | 0.96065 | 1997 | ASR | 91.06631 | 92.36127 | 90.90588 | 92.5217 | 90.71856 | 92.70902 | 90.48224 | 92.94534 | 89.83092 | 93.59666 |
| 9 | 91.96767 | 0.944334 | 1998 | ASR | 91.33119 | 92.60415 | 91.17348 | 92.76185 | 90.98934 | 92.946 | 90.75703 | 93.17831 | 90.11677 | 93.81856 |
| 10 | 92.21114 | 0.932343 | 1999 | ASR | 91.58274 | 92.83954 | 91.42704 | 92.99524 | 91.24523 | 93.17704 | 91.01587 | 93.4064 | 90.38375 | 94.03853 |
| 11 | 92.42778 | 0.92318 | 2000 | ASR | 91.80555 | 93.05 | 91.65138 | 93.20417 | 91.47136 | 93.38419 | 91.24426 | 93.61129 | 90.61834 | 94.23721 |
| 12 | 92.68405 | 0.916657 | 2001 | ASR | 92.06623 | 93.30188 | 91.91314 | 93.45496 | 91.7344 | 93.63371 | 91.5089 | 93.85921 | 90.8874 | 94.4807 |
| 13 | 92.94033 | 0.911488 | 2002 | ASR | 92.32599 | 93.55467 | 92.17377 | 93.70689 | 91.99603 | 93.88463 | 91.7718 | 94.10886 | 91.15381 | 94.72684 |
| 14 | 93.20584 | 0.907212 | 2003 | ASR | 92.59438 | 93.8173 | 92.44288 | 93.96881 | 92.26597 | 94.14571 | 92.04279 | 94.36889 | 91.4277 | 94.98398 |
| 15 | 93.452 | 0.903342 | 2004 | ASR | 92.84314 | 94.06085 | 92.69228 | 94.21171 | 92.51613 | 94.38786 | 92.29391 | 94.61008 | 91.68145 | 95.22255 |
| 16 | 93.69035 | 0.899785 | 2005 | ASR | 93.08389 | 94.2968 | 92.93363 | 94.44707 | 92.75817 | 94.62252 | 92.53682 | 94.84387 | 91.92677 | 95.45392 |
| 17 | 93.91022 | 0.896667 | 2006 | ASR | 93.30587 | 94.51457 | 93.15612 | 94.66432 | 92.98127 | 94.83917 | 92.76069 | 95.05975 | 92.15275 | 95.66769 |
| 18 | 94.09969 | 0.893686 | 2007 | ASR | 93.49734 | 94.70203 | 93.3481 | 94.85128 | 93.17383 | 95.02555 | 92.95398 | 95.2454 | 92.34806 | 95.85131 |
| 19 | 94.25203 | 0.890621 | 2008 | ASR | 93.65175 | 94.85231 | 93.50302 | 95.00104 | 93.32935 | 95.17471 | 93.11025 | 95.39381 | 92.50641 | 95.99765 |
| 20 | 94.339 | 0.887166 | 2009 | ASR | 93.74105 | 94.93695 | 93.5929 | 95.08511 | 93.4199 | 95.25811 | 93.20166 | 95.47635 | 92.60016 | 96.07785 |
| 21 | 94.3598 | 0.883171 | 2010 | ASR | 93.76454 | 94.95506 | 93.61705 | 95.10255 | 93.44484 | 95.27477 | 93.22758 | 95.49203 | 92.62879 | 96.09082 |
| 22 | 94.35436 | 0.879383 | 2011 | ASR | 93.76166 | 94.94706 | 93.6148 | 95.09392 | 93.44332 | 95.2654 | 93.22699 | 95.48173 | 92.63077 | 96.07795 |
| 23 | 94.3204 | 0.875829 | 2012 | ASR | 93.73009 | 94.91071 | 93.58383 | 95.05698 | 93.41304 | 95.22776 | 93.19759 | 95.44322 | 92.60378 | 96.03703 |
| 24 | 94.2903 | 0.872738 | 2013 | ASR | 93.70208 | 94.87853 | 93.55633 | 95.02428 | 93.38615 | 95.19446 | 93.17145 | 95.40916 | 92.57974 | 96.00087 |
| 25 | 94.28191 | 0.87043 | 2014 | ASR | 93.69524 | 94.86858 | 93.54988 | 95.01395 | 93.38015 | 95.18368 | 93.16602 | 95.39781 | 92.57587 | 95.98796 |
| 26 | 94.24246 | 0.868722 | 2015 | ASR | 93.65694 | 94.82798 | 93.51187 | 94.97306 | 93.34247 | 95.14246 | 93.12876 | 95.35616 | 92.53977 | 95.94516 |
| 27 | 94.1712 | 0.868761 | 2016 | ASR | 93.58565 | 94.75674 | 93.44057 | 94.90183 | 93.27116 | 95.07124 | 93.05745 | 95.28495 | 92.46843 | 95.87397 |
| 28 | 94.12134 | 0.872224 | 2017 | ASR | 93.53346 | 94.70922 | 93.3878 | 94.85488 | 93.21772 | 95.02496 | 93.00315 | 95.23953 | 92.41178 | 95.8309 |
| 29 | 94.00721 | 0.880407 | 2018 | ASR | 93.41382 | 94.60061 | 93.26679 | 94.74763 | 93.09511 | 94.91931 | 92.87853 | 95.13589 | 92.28161 | 95.73281 |
| 30 | 93.91842 | 0.898337 | 2019 | ASR | 93.31294 | 94.52389 | 93.16291 | 94.67392 | 92.98774 | 94.84909 | 92.76675 | 95.07008 | 92.15767 | 95.67916 |
| 31 | 93.83 | 0.933966 | 2020 | ASR | 93.20051 | 94.4595 | 93.04454 | 94.61547 | 92.86242 | 94.79759 | 92.63266 | 95.02735 | 91.99943 | 95.66058 |
| 32 | 93.56979 | 1.002563 | 2021 | ASR | 92.89406 | 94.24552 | 92.72663 | 94.41294 | 92.53113 | 94.60844 | 92.2845 | 94.85507 | 91.60477 | 95.53481 |
| 33 | 93.19559 | 1.270278 | 2022 | ASR | 92.33943 | 94.05176 | 92.12729 | 94.2639 | 91.87958 | 94.5116 | 91.5671 | 94.82409 | 90.70585 | 95.68534 |
| 34 | 92.8698 | 1.416043 | 2023 | ASR | 91.91539 | 93.82422 | 91.67891 | 94.06069 | 91.40278 | 94.33682 | 91.05443 | 94.68517 | 90.09436 | 95.64525 |
| 35 | 92.52714 | 1.555052 | 2024 | ASR | 91.47904 | 93.57525 | 91.21934 | 93.83494 | 90.91611 | 94.13817 | 90.53356 | 94.52072 | 89.47924 | 95.57504 |
| 36 | 92.16785 | 1.689674 | 2025 | ASR | 91.02901 | 93.30669 | 90.74683 | 93.58886 | 90.41735 | 93.91835 | 90.00169 | 94.33401 | 88.85609 | 95.47961 |
| 37 | 91.79297 | 1.821793 | 2026 | ASR | 90.56508 | 93.02086 | 90.26084 | 93.3251 | 89.90559 | 93.68035 | 89.45743 | 94.12851 | 88.22226 | 95.36368 |
| 38 | 91.40466 | 1.952647 | 2027 | ASR | 90.08858 | 92.72074 | 89.76248 | 93.04683 | 89.38172 | 93.4276 | 88.90137 | 93.90795 | 87.57747 | 95.23185 |
| 39 | 91.00519 | 2.082312 | 2028 | ASR | 89.60171 | 92.40867 | 89.25396 | 92.75641 | 88.84791 | 93.16246 | 88.33566 | 93.67471 | 86.92386 | 95.08652 |
| 40 | 90.59584 | 2.211663 | 2029 | ASR | 89.10518 | 92.0865 | 88.73583 | 92.45585 | 88.30456 | 92.88712 | 87.76049 | 93.43119 | 86.26098 | 94.9307 |
| 41 | 90.17708 | 2.341975 | 2030 | ASR | 88.59859 | 91.75557 | 88.20748 | 92.14668 | 87.75079 | 92.60337 | 87.17467 | 93.17949 | 85.58681 | 94.76735 |
| 42 | 89.75017 | 2.474571 | 2031 | ASR | 88.08231 | 91.41803 | 87.66906 | 91.83129 | 87.18652 | 92.31383 | 86.57777 | 92.92257 | 84.90001 | 94.60033 |
| 43 | 89.31711 | 2.610523 | 2032 | ASR | 87.55762 | 91.0766 | 87.12166 | 91.51256 | 86.61261 | 92.02161 | 85.97042 | 92.6638 | 84.20048 | 94.43373 |
| 44 | 88.87968 | 2.750081 | 2033 | ASR | 87.02613 | 90.73324 | 86.56686 | 91.1925 | 86.0306 | 91.72877 | 85.35408 | 92.40529 | 83.48952 | 94.26984 |
| 45 | 88.43899 | 2.894231 | 2034 | ASR | 86.48827 | 90.3897 | 86.00494 | 90.87303 | 85.44056 | 91.43741 | 84.72858 | 92.14939 | 82.76629 | 94.11168 |
| 46 | 87.99538 | 3.044375 | 2035 | ASR | 85.94347 | 90.04728 | 85.43506 | 90.5557 | 84.8414 | 91.14935 | 84.09249 | 91.89827 | 82.0284 | 93.96235 |
| **Malaysia** | | | | | | | | | | | | | | |
| 1 | 78.29583 | 0.729007 | 1990 | ASR | 77.80448 | 78.78718 | 77.68274 | 78.90893 | 77.54058 | 79.05108 | 77.36125 | 79.23042 | 76.86698 | 79.72469 |
| 2 | 78.2283 | 0.649149 | 1991 | ASR | 77.79077 | 78.66582 | 77.68236 | 78.77423 | 77.55578 | 78.90082 | 77.39609 | 79.06051 | 76.95597 | 79.50063 |
| 3 | 78.37899 | 0.62349 | 1992 | ASR | 77.95876 | 78.79923 | 77.85464 | 78.90335 | 77.73306 | 79.02493 | 77.57968 | 79.17831 | 77.15696 | 79.60103 |
| 4 | 78.59544 | 0.614247 | 1993 | ASR | 78.18144 | 79.00944 | 78.07886 | 79.11202 | 77.95908 | 79.2318 | 77.80797 | 79.3829 | 77.39151 | 79.79936 |
| 5 | 78.41698 | 0.609475 | 1994 | ASR | 78.00619 | 78.82777 | 77.90441 | 78.92955 | 77.78556 | 79.0484 | 77.63563 | 79.19833 | 77.22241 | 79.61155 |
| 6 | 78.75642 | 0.605937 | 1995 | ASR | 78.34802 | 79.16482 | 78.24682 | 79.26601 | 78.12867 | 79.38417 | 77.97961 | 79.53323 | 77.56878 | 79.94405 |
| 7 | 79.10024 | 0.60083 | 1996 | ASR | 78.69528 | 79.5052 | 78.59494 | 79.60553 | 78.47778 | 79.7227 | 78.32997 | 79.8705 | 77.92261 | 80.27786 |
| 8 | 79.68592 | 0.597845 | 1997 | ASR | 79.28298 | 80.08887 | 79.18314 | 80.18871 | 79.06656 | 80.30529 | 78.91949 | 80.45236 | 78.51415 | 80.8577 |
| 9 | 80.34112 | 0.598634 | 1998 | ASR | 79.93764 | 80.7446 | 79.83767 | 80.84457 | 79.72093 | 80.9613 | 79.57367 | 81.10857 | 79.1678 | 81.51444 |
| 10 | 81.18892 | 0.606472 | 1999 | ASR | 80.78016 | 81.59768 | 80.67888 | 81.69896 | 80.56061 | 81.81722 | 80.41142 | 81.96641 | 80.00023 | 82.3776 |
| 11 | 80.50432 | 0.591735 | 2000 | ASR | 80.10549 | 80.90315 | 80.00667 | 81.00197 | 79.89128 | 81.11736 | 79.74572 | 81.26292 | 79.34452 | 81.66412 |
| 12 | 79.76128 | 0.580247 | 2001 | ASR | 79.37019 | 80.15237 | 79.27329 | 80.24927 | 79.16014 | 80.36242 | 79.0174 | 80.50516 | 78.624 | 80.89856 |
| 13 | 79.38077 | 0.574081 | 2002 | ASR | 78.99384 | 79.7677 | 78.89797 | 79.86357 | 78.78602 | 79.97552 | 78.6448 | 80.11674 | 78.25557 | 80.50597 |
| 14 | 79.48006 | 0.569469 | 2003 | ASR | 79.09624 | 79.86389 | 79.00114 | 79.95899 | 78.89009 | 80.07003 | 78.75001 | 80.21012 | 78.36391 | 80.59622 |
| 15 | 79.22355 | 0.563908 | 2004 | ASR | 78.84348 | 79.60363 | 78.74931 | 79.6978 | 78.63935 | 79.80776 | 78.50062 | 79.94648 | 78.11829 | 80.32881 |
| 16 | 78.85696 | 0.557061 | 2005 | ASR | 78.4815 | 79.23242 | 78.38847 | 79.32545 | 78.27985 | 79.43408 | 78.14281 | 79.57111 | 77.76512 | 79.9488 |
| 17 | 78.49184 | 0.548351 | 2006 | ASR | 78.12225 | 78.86143 | 78.03068 | 78.95301 | 77.92375 | 79.05993 | 77.78886 | 79.19483 | 77.41707 | 79.56661 |
| 18 | 78.31502 | 0.541442 | 2007 | ASR | 77.95009 | 78.67995 | 77.85966 | 78.77037 | 77.75408 | 78.87595 | 77.62089 | 79.00915 | 77.25379 | 79.37624 |
| 19 | 78.45991 | 0.535764 | 2008 | ASR | 78.09881 | 78.82102 | 78.00934 | 78.91049 | 77.90486 | 79.01497 | 77.77307 | 79.14676 | 77.40982 | 79.51001 |
| 20 | 78.64179 | 0.531351 | 2009 | ASR | 78.28366 | 78.99992 | 78.19492 | 79.08865 | 78.09131 | 79.19227 | 77.96059 | 79.32298 | 77.60034 | 79.68323 |
| 21 | 78.60072 | 0.524976 | 2010 | ASR | 78.24689 | 78.95456 | 78.15922 | 79.04223 | 78.05685 | 79.1446 | 77.9277 | 79.27374 | 77.57177 | 79.62968 |
| 22 | 78.15194 | 0.514381 | 2011 | ASR | 77.80524 | 78.49863 | 77.71934 | 78.58453 | 77.61904 | 78.68483 | 77.4925 | 78.81137 | 77.14375 | 79.16012 |
| 23 | 77.59062 | 0.505918 | 2012 | ASR | 77.24963 | 77.93161 | 77.16514 | 78.0161 | 77.06649 | 78.11475 | 76.94203 | 78.23921 | 76.59902 | 78.58222 |
| 24 | 77.25227 | 0.498384 | 2013 | ASR | 76.91636 | 77.58818 | 76.83313 | 77.67141 | 76.73594 | 77.7686 | 76.61334 | 77.8912 | 76.27544 | 78.2291 |
| 25 | 77.2186 | 0.490936 | 2014 | ASR | 76.88771 | 77.54949 | 76.80572 | 77.63148 | 76.70999 | 77.72721 | 76.58922 | 77.84798 | 76.25637 | 78.18084 |
| 26 | 77.40719 | 0.484845 | 2015 | ASR | 77.0804 | 77.73397 | 76.99943 | 77.81494 | 76.90489 | 77.90949 | 76.78562 | 78.02876 | 76.45689 | 78.35748 |
| 27 | 77.16094 | 0.476691 | 2016 | ASR | 76.83965 | 77.48223 | 76.76004 | 77.56184 | 76.66709 | 77.65479 | 76.54982 | 77.77206 | 76.22663 | 78.09526 |
| 28 | 76.98373 | 0.470582 | 2017 | ASR | 76.66655 | 77.3009 | 76.58797 | 77.37949 | 76.4962 | 77.47125 | 76.38044 | 77.58701 | 76.06139 | 77.90607 |
| 29 | 76.43527 | 0.462062 | 2018 | ASR | 76.12384 | 76.7467 | 76.04668 | 76.82387 | 75.95658 | 76.91397 | 75.84291 | 77.02764 | 75.52963 | 77.34091 |
| 30 | 75.65839 | 0.452905 | 2019 | ASR | 75.35313 | 75.96365 | 75.2775 | 76.03928 | 75.18918 | 76.1276 | 75.07776 | 76.23901 | 74.77069 | 76.54608 |
| 31 | 73.44768 | 0.450616 | 2020 | ASR | 73.14396 | 73.75139 | 73.06871 | 73.82664 | 72.98084 | 73.91451 | 72.86999 | 74.02536 | 72.56447 | 74.33088 |
| 32 | 75.16551 | 0.477701 | 2021 | ASR | 74.84354 | 75.48748 | 74.76376 | 75.56725 | 74.67061 | 75.6604 | 74.55309 | 75.77792 | 74.22921 | 76.1018 |
| 33 | 74.27334 | 0.932814 | 2022 | ASR | 73.64463 | 74.90206 | 73.48885 | 75.05784 | 73.30695 | 75.23974 | 73.07748 | 75.46921 | 72.44503 | 76.10166 |
| 34 | 73.84693 | 1.148924 | 2023 | ASR | 73.07256 | 74.62131 | 72.88069 | 74.81318 | 72.65665 | 75.03722 | 72.37401 | 75.31985 | 71.59504 | 76.09882 |
| 35 | 73.42864 | 1.335826 | 2024 | ASR | 72.52829 | 74.32898 | 72.30521 | 74.55207 | 72.04472 | 74.81255 | 71.71611 | 75.14117 | 70.81042 | 76.04686 |
| 36 | 73.02012 | 1.505384 | 2025 | ASR | 72.00549 | 74.03475 | 71.75409 | 74.28615 | 71.46054 | 74.5797 | 71.09022 | 74.95002 | 70.06957 | 75.97067 |
| 37 | 72.64051 | 1.664788 | 2026 | ASR | 71.51845 | 73.76258 | 71.24043 | 74.0406 | 70.91579 | 74.36523 | 70.50626 | 74.77477 | 69.37753 | 75.9035 |
| 38 | 72.26556 | 1.817471 | 2027 | ASR | 71.04059 | 73.49054 | 70.73707 | 73.79406 | 70.38266 | 74.14846 | 69.93557 | 74.59556 | 68.70332 | 75.82781 |
| 39 | 71.90093 | 1.963736 | 2028 | ASR | 70.57737 | 73.22449 | 70.24943 | 73.55243 | 69.8665 | 73.93536 | 69.38342 | 74.41844 | 68.05201 | 75.74985 |
| 40 | 71.54643 | 2.105064 | 2029 | ASR | 70.12762 | 72.96524 | 69.77607 | 73.31679 | 69.36558 | 73.72727 | 68.84774 | 74.24512 | 67.4205 | 75.67235 |
| 41 | 71.20184 | 2.243544 | 2030 | ASR | 69.68969 | 72.71398 | 69.31502 | 73.08866 | 68.87752 | 73.52615 | 68.32561 | 74.07806 | 66.80449 | 75.59918 |
| 42 | 70.88143 | 2.381625 | 2031 | ASR | 69.27621 | 72.48664 | 68.87848 | 72.88437 | 68.41406 | 73.34879 | 67.82818 | 73.93467 | 66.21344 | 75.54941 |
| 43 | 70.5698 | 2.520481 | 2032 | ASR | 68.871 | 72.26861 | 68.45008 | 72.68953 | 67.95858 | 73.18102 | 67.33855 | 73.80106 | 65.62966 | 75.50995 |
| 44 | 70.27482 | 2.660002 | 2033 | ASR | 68.48198 | 72.06766 | 68.03776 | 72.51188 | 67.51906 | 73.03058 | 66.86469 | 73.68494 | 65.06121 | 75.48842 |
| 45 | 69.99584 | 2.801017 | 2034 | ASR | 68.10796 | 71.88373 | 67.64019 | 72.3515 | 67.09399 | 72.8977 | 66.40494 | 73.58675 | 64.50585 | 75.48584 |
| 46 | 69.73078 | 2.945012 | 2035 | ASR | 67.74584 | 71.71572 | 67.25403 | 72.20754 | 66.67975 | 72.78181 | 65.95528 | 73.50629 | 63.95856 | 75.503 |
| **Myanmar** | | | | | | | | | | | | | | |
| 1 | 139.9263 | 0.777151 | 1990 | ASR | 139.4025 | 140.4501 | 139.2727 | 140.5799 | 139.1212 | 140.7314 | 138.93 | 140.9226 | 138.4031 | 141.4495 |
| 2 | 140.5381 | 0.703038 | 1991 | ASR | 140.0643 | 141.0119 | 139.9468 | 141.1294 | 139.8098 | 141.2664 | 139.6368 | 141.4394 | 139.1601 | 141.9161 |
| 3 | 141.0814 | 0.680793 | 1992 | ASR | 140.6225 | 141.5402 | 140.5088 | 141.6539 | 140.376 | 141.7867 | 140.2086 | 141.9541 | 139.747 | 142.4157 |
| 4 | 141.5759 | 0.669759 | 1993 | ASR | 141.1245 | 142.0273 | 141.0126 | 142.1392 | 140.882 | 142.2698 | 140.7173 | 142.4345 | 140.2632 | 142.8886 |
| 5 | 141.9843 | 0.661735 | 1994 | ASR | 141.5383 | 142.4304 | 141.4278 | 142.5409 | 141.2988 | 142.6699 | 141.136 | 142.8327 | 140.6873 | 143.2813 |
| 6 | 142.3175 | 0.655042 | 1995 | ASR | 141.876 | 142.759 | 141.7666 | 142.8684 | 141.6389 | 142.9961 | 141.4777 | 143.1573 | 141.0336 | 143.6014 |
| 7 | 142.6604 | 0.649129 | 1996 | ASR | 142.2229 | 143.0979 | 142.1145 | 143.2063 | 141.9879 | 143.3329 | 141.8282 | 143.4926 | 141.3881 | 143.9327 |
| 8 | 142.9765 | 0.64364 | 1997 | ASR | 142.5427 | 143.4103 | 142.4352 | 143.5178 | 142.3097 | 143.6433 | 142.1513 | 143.8016 | 141.7149 | 144.238 |
| 9 | 143.2549 | 0.63836 | 1998 | ASR | 142.8247 | 143.6852 | 142.7181 | 143.7918 | 142.5936 | 143.9163 | 142.4366 | 144.0733 | 142.0037 | 144.5061 |
| 10 | 143.4216 | 0.632876 | 1999 | ASR | 142.9951 | 143.8482 | 142.8894 | 143.9539 | 142.766 | 144.0773 | 142.6103 | 144.233 | 142.1812 | 144.6621 |
| 11 | 143.4548 | 0.627341 | 2000 | ASR | 143.032 | 143.8776 | 142.9272 | 143.9824 | 142.8049 | 144.1047 | 142.6506 | 144.2591 | 142.2252 | 144.6844 |
| 12 | 143.3561 | 0.621371 | 2001 | ASR | 142.9373 | 143.7749 | 142.8336 | 143.8787 | 142.7124 | 143.9999 | 142.5595 | 144.1527 | 142.1383 | 144.574 |
| 13 | 143.1403 | 0.615158 | 2002 | ASR | 142.7256 | 143.5549 | 142.6229 | 143.6576 | 142.503 | 143.7776 | 142.3516 | 143.9289 | 141.9346 | 144.346 |
| 14 | 142.8322 | 0.608768 | 2003 | ASR | 142.4219 | 143.2426 | 142.3203 | 143.3442 | 142.2016 | 143.4629 | 142.0518 | 143.6127 | 141.6391 | 144.0254 |
| 15 | 142.2909 | 0.601761 | 2004 | ASR | 141.8853 | 142.6965 | 141.7848 | 142.7969 | 141.6674 | 142.9143 | 141.5194 | 143.0623 | 141.1114 | 143.4703 |
| 16 | 141.3528 | 0.593655 | 2005 | ASR | 140.9527 | 141.7529 | 140.8535 | 141.852 | 140.7378 | 141.9678 | 140.5917 | 142.1138 | 140.1892 | 142.5163 |
| 17 | 140.457 | 0.585476 | 2006 | ASR | 140.0624 | 140.8516 | 139.9646 | 140.9494 | 139.8504 | 141.0635 | 139.7064 | 141.2076 | 139.3094 | 141.6045 |
| 18 | 139.385 | 0.576839 | 2007 | ASR | 138.9962 | 139.7738 | 138.8999 | 139.8702 | 138.7874 | 139.9826 | 138.6455 | 140.1245 | 138.2544 | 140.5156 |
| 19 | 138.419 | 0.569728 | 2008 | ASR | 138.035 | 138.803 | 137.9398 | 138.8981 | 137.8287 | 139.0092 | 137.6886 | 139.1493 | 137.3023 | 139.5356 |
| 20 | 137.5034 | 0.562653 | 2009 | ASR | 137.1242 | 137.8826 | 137.0302 | 137.9766 | 136.9205 | 138.0863 | 136.7821 | 138.2247 | 136.4006 | 138.6062 |
| 21 | 136.5951 | 0.554716 | 2010 | ASR | 136.2212 | 136.9689 | 136.1285 | 137.0616 | 136.0204 | 137.1697 | 135.8839 | 137.3062 | 135.5078 | 137.6823 |
| 22 | 136.0131 | 0.547149 | 2011 | ASR | 135.6443 | 136.3818 | 135.5529 | 136.4732 | 135.4462 | 136.5799 | 135.3116 | 136.7145 | 134.9406 | 137.0855 |
| 23 | 135.1534 | 0.539252 | 2012 | ASR | 134.7899 | 135.5168 | 134.6998 | 135.6069 | 134.5947 | 135.712 | 134.462 | 135.8447 | 134.0964 | 136.2103 |
| 24 | 134.8663 | 0.532649 | 2013 | ASR | 134.5073 | 135.2253 | 134.4184 | 135.3143 | 134.3145 | 135.4182 | 134.1835 | 135.5492 | 133.8223 | 135.9103 |
| 25 | 134.7718 | 0.526477 | 2014 | ASR | 134.4169 | 135.1266 | 134.329 | 135.2145 | 134.2263 | 135.3172 | 134.0968 | 135.4467 | 133.7399 | 135.8037 |
| 26 | 134.8248 | 0.521433 | 2015 | ASR | 134.4734 | 135.1763 | 134.3863 | 135.2633 | 134.2846 | 135.365 | 134.1563 | 135.4933 | 133.8028 | 135.8468 |
| 27 | 134.9635 | 0.51644 | 2016 | ASR | 134.6154 | 135.3116 | 134.5292 | 135.3978 | 134.4285 | 135.4985 | 134.3014 | 135.6256 | 133.9513 | 135.9757 |
| 28 | 135.3313 | 0.512233 | 2017 | ASR | 134.986 | 135.6765 | 134.9005 | 135.762 | 134.8006 | 135.8619 | 134.6746 | 135.9879 | 134.3273 | 136.3352 |
| 29 | 135.7664 | 0.508503 | 2018 | ASR | 135.4237 | 136.1092 | 135.3388 | 136.1941 | 135.2396 | 136.2933 | 135.1145 | 136.4183 | 134.7698 | 136.7631 |
| 30 | 136.3923 | 0.505683 | 2019 | ASR | 136.0515 | 136.7332 | 135.9671 | 136.8176 | 135.8684 | 136.9162 | 135.744 | 137.0406 | 135.4012 | 137.3835 |
| 31 | 137.2264 | 0.507613 | 2020 | ASR | 136.8843 | 137.5685 | 136.7995 | 137.6533 | 136.7005 | 137.7523 | 136.5756 | 137.8772 | 136.2315 | 138.2213 |
| 32 | 137.6682 | 0.535398 | 2021 | ASR | 137.3074 | 138.0291 | 137.2179 | 138.1185 | 137.1135 | 138.2229 | 136.9818 | 138.3546 | 136.6188 | 138.7176 |
| 33 | 136.535 | 1.25877 | 2022 | ASR | 135.6866 | 137.3834 | 135.4764 | 137.5937 | 135.2309 | 137.8391 | 134.9213 | 138.1488 | 134.0678 | 139.0022 |
| 34 | 135.8617 | 1.590311 | 2023 | ASR | 134.7899 | 136.9336 | 134.5243 | 137.1992 | 134.2142 | 137.5093 | 133.823 | 137.9005 | 132.7447 | 138.9788 |
| 35 | 135.1755 | 1.872758 | 2024 | ASR | 133.9133 | 136.4377 | 133.6005 | 136.7505 | 133.2353 | 137.1157 | 132.7746 | 137.5764 | 131.5049 | 138.8461 |
| 36 | 134.4732 | 2.12595 | 2025 | ASR | 133.0403 | 135.9061 | 132.6853 | 136.2611 | 132.2707 | 136.6757 | 131.7478 | 137.1987 | 130.3064 | 138.6401 |
| 37 | 133.7589 | 2.360337 | 2026 | ASR | 132.168 | 135.3497 | 131.7738 | 135.7439 | 131.3136 | 136.2042 | 130.7329 | 136.7848 | 129.1326 | 138.3851 |
| 38 | 133.0307 | 2.582059 | 2027 | ASR | 131.2904 | 134.771 | 130.8592 | 135.2022 | 130.3557 | 135.7057 | 129.7205 | 136.3409 | 127.9699 | 138.0915 |
| 39 | 132.2983 | 2.792745 | 2028 | ASR | 130.416 | 134.1806 | 129.9496 | 134.647 | 129.405 | 135.1916 | 128.718 | 135.8786 | 126.8246 | 137.7721 |
| 40 | 131.5634 | 2.994402 | 2029 | ASR | 129.5451 | 133.5816 | 129.0451 | 134.0816 | 128.4612 | 134.6656 | 127.7245 | 135.4022 | 125.6943 | 137.4324 |
| 41 | 130.8243 | 3.189275 | 2030 | ASR | 128.6747 | 132.9738 | 128.1421 | 133.5064 | 127.5202 | 134.1284 | 126.7356 | 134.9129 | 124.5733 | 137.0752 |
| 42 | 130.0859 | 3.379803 | 2031 | ASR | 127.8079 | 132.3638 | 127.2434 | 132.9283 | 126.5844 | 133.5873 | 125.7529 | 134.4188 | 123.4614 | 136.7103 |
| 43 | 129.3472 | 3.567995 | 2032 | ASR | 126.9424 | 131.752 | 126.3465 | 132.3479 | 125.6507 | 133.0436 | 124.773 | 133.9214 | 122.3539 | 136.3405 |
| 44 | 128.6131 | 3.753714 | 2033 | ASR | 126.0831 | 131.1431 | 125.4562 | 131.77 | 124.7242 | 132.5019 | 123.8008 | 133.4253 | 121.2558 | 135.9704 |
| 45 | 127.8853 | 3.937696 | 2034 | ASR | 125.2313 | 130.5393 | 124.5737 | 131.1969 | 123.8059 | 131.9648 | 122.8372 | 132.9334 | 120.1674 | 135.6032 |
| 46 | 127.1638 | 4.121298 | 2035 | ASR | 124.386 | 129.9415 | 123.6977 | 130.6298 | 122.8941 | 131.4334 | 121.8803 | 132.4473 | 119.086 | 135.2415 |
| **Philippines** | | | | | | | | | | | | | | |
| 1 | 72.47496 | 0.510122 | 1990 | ASR | 72.13114 | 72.81879 | 72.04595 | 72.90398 | 71.94648 | 73.00345 | 71.82099 | 73.12894 | 71.47512 | 73.4748 |
| 2 | 71.78191 | 0.443778 | 1991 | ASR | 71.48281 | 72.08102 | 71.4087 | 72.15513 | 71.32216 | 72.24167 | 71.21299 | 72.35084 | 70.91211 | 72.65172 |
| 3 | 71.48355 | 0.418195 | 1992 | ASR | 71.20169 | 71.76541 | 71.13185 | 71.83525 | 71.0503 | 71.9168 | 70.94742 | 72.01968 | 70.66389 | 72.30321 |
| 4 | 71.19893 | 0.402798 | 1993 | ASR | 70.92744 | 71.47042 | 70.86018 | 71.53768 | 70.78163 | 71.61623 | 70.68254 | 71.71532 | 70.40945 | 71.98842 |
| 5 | 70.96302 | 0.391932 | 1994 | ASR | 70.69886 | 71.22719 | 70.63341 | 71.29264 | 70.55698 | 71.36907 | 70.46057 | 71.46548 | 70.19484 | 71.73121 |
| 6 | 70.72727 | 0.383609 | 1995 | ASR | 70.46871 | 70.98582 | 70.40465 | 71.04988 | 70.32985 | 71.12469 | 70.23548 | 71.21905 | 69.97539 | 71.47914 |
| 7 | 70.47105 | 0.376751 | 1996 | ASR | 70.21712 | 70.72498 | 70.15421 | 70.7879 | 70.08074 | 70.86137 | 69.98806 | 70.95405 | 69.73262 | 71.20948 |
| 8 | 69.97362 | 0.369828 | 1997 | ASR | 69.72436 | 70.22289 | 69.6626 | 70.28465 | 69.59048 | 70.35677 | 69.49951 | 70.44774 | 69.24876 | 70.69849 |
| 9 | 69.63184 | 0.364105 | 1998 | ASR | 69.38643 | 69.87725 | 69.32563 | 69.93805 | 69.25462 | 70.00905 | 69.16505 | 70.09862 | 68.91819 | 70.34548 |
| 10 | 69.26949 | 0.358631 | 1999 | ASR | 69.02777 | 69.5112 | 68.96788 | 69.5711 | 68.89795 | 69.64103 | 68.80972 | 69.72925 | 68.56657 | 69.9724 |
| 11 | 69.08541 | 0.35389 | 2000 | ASR | 68.84689 | 69.32393 | 68.78779 | 69.38303 | 68.71878 | 69.45204 | 68.63172 | 69.5391 | 68.39179 | 69.77903 |
| 12 | 68.97514 | 0.349587 | 2001 | ASR | 68.73951 | 69.21076 | 68.68113 | 69.26914 | 68.61296 | 69.33731 | 68.52697 | 69.42331 | 68.28995 | 69.66033 |
| 13 | 68.86805 | 0.345556 | 2002 | ASR | 68.63515 | 69.10096 | 68.57744 | 69.15867 | 68.51006 | 69.22605 | 68.42505 | 69.31106 | 68.19076 | 69.54534 |
| 14 | 68.53704 | 0.340919 | 2003 | ASR | 68.30726 | 68.76682 | 68.25033 | 68.82375 | 68.18385 | 68.89023 | 68.09998 | 68.9741 | 67.86884 | 69.20524 |
| 15 | 68.35771 | 0.336839 | 2004 | ASR | 68.13068 | 68.58474 | 68.07443 | 68.64099 | 68.00874 | 68.70668 | 67.92588 | 68.78954 | 67.6975 | 69.01791 |
| 16 | 68.53453 | 0.333592 | 2005 | ASR | 68.30969 | 68.75937 | 68.25398 | 68.81508 | 68.18893 | 68.88013 | 68.10687 | 68.9622 | 67.88069 | 69.18837 |
| 17 | 68.88567 | 0.330985 | 2006 | ASR | 68.66259 | 69.10876 | 68.60731 | 69.16403 | 68.54277 | 69.22857 | 68.46135 | 69.31 | 68.23694 | 69.5344 |
| 18 | 69.32079 | 0.328628 | 2007 | ASR | 69.0993 | 69.54229 | 69.04442 | 69.59717 | 68.98033 | 69.66125 | 68.89949 | 69.74209 | 68.67668 | 69.9649 |
| 19 | 69.98535 | 0.326745 | 2008 | ASR | 69.76512 | 70.20558 | 69.71056 | 70.26014 | 69.64684 | 70.32386 | 69.56646 | 70.40424 | 69.34493 | 70.62577 |
| 20 | 70.54258 | 0.324615 | 2009 | ASR | 70.32379 | 70.76137 | 70.26958 | 70.81558 | 70.20628 | 70.87888 | 70.12642 | 70.95874 | 69.90633 | 71.17882 |
| 21 | 70.89259 | 0.321802 | 2010 | ASR | 70.6757 | 71.10949 | 70.62196 | 71.16323 | 70.5592 | 71.22598 | 70.48004 | 71.30514 | 70.26186 | 71.52332 |
| 22 | 71.02921 | 0.318321 | 2011 | ASR | 70.81466 | 71.24376 | 70.7615 | 71.29692 | 70.69943 | 71.35899 | 70.62112 | 71.4373 | 70.4053 | 71.65312 |
| 23 | 71.1356 | 0.314868 | 2012 | ASR | 70.92338 | 71.34782 | 70.87079 | 71.4004 | 70.80939 | 71.4618 | 70.73194 | 71.53926 | 70.51846 | 71.75274 |
| 24 | 71.08637 | 0.311123 | 2013 | ASR | 70.87667 | 71.29606 | 70.82471 | 71.34802 | 70.76404 | 71.40869 | 70.68751 | 71.48523 | 70.47656 | 71.69617 |
| 25 | 70.89902 | 0.307183 | 2014 | ASR | 70.69197 | 71.10606 | 70.64068 | 71.15736 | 70.58077 | 71.21726 | 70.50521 | 71.29283 | 70.29694 | 71.5011 |
| 26 | 71.24623 | 0.304371 | 2015 | ASR | 71.04109 | 71.45138 | 70.99026 | 71.50221 | 70.93091 | 71.56156 | 70.85603 | 71.63644 | 70.64967 | 71.8428 |
| 27 | 71.65671 | 0.301869 | 2016 | ASR | 71.45325 | 71.86017 | 71.40284 | 71.91058 | 71.34397 | 71.96945 | 71.26971 | 72.04371 | 71.06505 | 72.24837 |
| 28 | 72.05158 | 0.299266 | 2017 | ASR | 71.84987 | 72.25328 | 71.7999 | 72.30326 | 71.74154 | 72.36162 | 71.66792 | 72.43524 | 71.46502 | 72.63814 |
| 29 | 72.52554 | 0.296736 | 2018 | ASR | 72.32554 | 72.72555 | 72.27599 | 72.7751 | 72.21813 | 72.83296 | 72.14513 | 72.90596 | 71.94394 | 73.10715 |
| 30 | 72.83631 | 0.294059 | 2019 | ASR | 72.63812 | 73.03451 | 72.58901 | 73.08362 | 72.53167 | 73.14096 | 72.45933 | 73.2133 | 72.25996 | 73.41267 |
| 31 | 72.34617 | 0.290239 | 2020 | ASR | 72.15054 | 72.54179 | 72.10207 | 72.59026 | 72.04548 | 72.64685 | 71.97408 | 72.71825 | 71.7773 | 72.91504 |
| 32 | 72.63371 | 0.303264 | 2021 | ASR | 72.42931 | 72.83811 | 72.37867 | 72.88876 | 72.31953 | 72.9479 | 72.24493 | 73.0225 | 72.03932 | 73.22811 |
| 33 | 72.41486 | 0.78174 | 2022 | ASR | 71.88797 | 72.94176 | 71.75742 | 73.07231 | 71.60498 | 73.22475 | 71.41267 | 73.41705 | 70.88265 | 73.94707 |
| 34 | 72.22086 | 1.003575 | 2023 | ASR | 71.54445 | 72.89727 | 71.37685 | 73.06486 | 71.18115 | 73.26056 | 70.93427 | 73.50744 | 70.25385 | 74.18787 |
| 35 | 72.01028 | 1.192044 | 2024 | ASR | 71.20684 | 72.81371 | 71.00777 | 73.01279 | 70.77532 | 73.24523 | 70.48208 | 73.53848 | 69.67387 | 74.34668 |
| 36 | 71.78503 | 1.36109 | 2025 | ASR | 70.86765 | 72.7024 | 70.64035 | 72.92971 | 70.37494 | 73.19512 | 70.04011 | 73.52995 | 69.11729 | 74.45276 |
| 37 | 71.55001 | 1.517793 | 2026 | ASR | 70.52702 | 72.57301 | 70.27355 | 72.82648 | 69.97758 | 73.12245 | 69.6042 | 73.49582 | 68.57514 | 74.52489 |
| 38 | 71.30376 | 1.666261 | 2027 | ASR | 70.1807 | 72.42682 | 69.90244 | 72.70509 | 69.57751 | 73.03001 | 69.16761 | 73.43991 | 68.03789 | 74.56963 |
| 39 | 71.04461 | 1.807637 | 2028 | ASR | 69.82626 | 72.26295 | 69.52438 | 72.56483 | 69.1719 | 72.91732 | 68.72722 | 73.362 | 67.50164 | 74.58758 |
| 40 | 70.77124 | 1.943145 | 2029 | ASR | 69.46156 | 72.08092 | 69.13706 | 72.40542 | 68.75814 | 72.78434 | 68.28013 | 73.26235 | 66.96268 | 74.5798 |
| 41 | 70.48551 | 2.074162 | 2030 | ASR | 69.08752 | 71.88349 | 68.74114 | 72.22988 | 68.33668 | 72.63434 | 67.82643 | 73.14458 | 66.42015 | 74.55087 |
| 42 | 70.1916 | 2.202169 | 2031 | ASR | 68.70734 | 71.67586 | 68.33958 | 72.04362 | 67.91015 | 72.47305 | 67.36842 | 73.01478 | 65.87535 | 74.50785 |
| 43 | 69.88869 | 2.328375 | 2032 | ASR | 68.31937 | 71.45802 | 67.93053 | 71.84685 | 67.47649 | 72.30089 | 66.90371 | 72.87367 | 65.32508 | 74.45231 |
| 44 | 69.57616 | 2.452661 | 2033 | ASR | 67.92307 | 71.22926 | 67.51347 | 71.63885 | 67.0352 | 72.11712 | 66.43185 | 72.72047 | 64.76895 | 74.38338 |
| 45 | 69.25362 | 2.575376 | 2034 | ASR | 67.51782 | 70.98942 | 67.08773 | 71.41951 | 66.58553 | 71.92171 | 65.95199 | 72.55525 | 64.20588 | 74.30136 |
| 46 | 68.92275 | 2.697286 | 2035 | ASR | 67.10477 | 70.74072 | 66.65433 | 71.19116 | 66.12836 | 71.71713 | 65.46482 | 72.38067 | 63.63606 | 74.20943 |
| **Singapore** | | | | | | | | | | | | | | |
| 1 | 93.60583 | 1.574451 | 1990 | ASR | 92.54465 | 94.66701 | 92.28172 | 94.92994 | 91.9747 | 95.23696 | 91.58738 | 95.62428 | 90.5199 | 96.69175 |
| 2 | 94.52862 | 1.365762 | 1991 | ASR | 93.6081 | 95.44914 | 93.38001 | 95.67723 | 93.11369 | 95.94355 | 92.77771 | 96.27953 | 91.85173 | 97.20551 |
| 3 | 95.73486 | 1.300369 | 1992 | ASR | 94.85841 | 96.61131 | 94.64125 | 96.82847 | 94.38768 | 97.08204 | 94.06779 | 97.40194 | 93.18614 | 98.28359 |
| 4 | 96.46895 | 1.274585 | 1993 | ASR | 95.60988 | 97.32802 | 95.39702 | 97.54088 | 95.14848 | 97.78942 | 94.83493 | 98.10297 | 93.97076 | 98.96714 |
| 5 | 97.37938 | 1.262064 | 1994 | ASR | 96.52875 | 98.23001 | 96.31798 | 98.44078 | 96.07188 | 98.68688 | 95.76141 | 98.99735 | 94.90574 | 99.85302 |
| 6 | 98.75773 | 1.258189 | 1995 | ASR | 97.90971 | 99.60575 | 97.69959 | 99.81587 | 97.45425 | 100.0612 | 97.14473 | 100.3707 | 96.29168 | 101.2238 |
| 7 | 99.62538 | 1.249774 | 1996 | ASR | 98.78303 | 100.4677 | 98.57432 | 100.6764 | 98.33061 | 100.9201 | 98.02317 | 101.2276 | 97.17582 | 102.0749 |
| 8 | 100.5315 | 1.242762 | 1997 | ASR | 99.69383 | 101.3691 | 99.48629 | 101.5766 | 99.24395 | 101.819 | 98.93823 | 102.1247 | 98.09564 | 102.9673 |
| 9 | 100.9016 | 1.229674 | 1998 | ASR | 100.0728 | 101.7304 | 99.86747 | 101.9358 | 99.62769 | 102.1756 | 99.32519 | 102.4781 | 98.49147 | 103.3118 |
| 10 | 101.7 | 1.223025 | 1999 | ASR | 100.8757 | 102.5244 | 100.6715 | 102.7286 | 100.433 | 102.9671 | 100.1321 | 103.268 | 99.30291 | 104.0972 |
| 11 | 102.5845 | 1.217318 | 2000 | ASR | 101.764 | 103.405 | 101.5607 | 103.6083 | 101.3234 | 103.8456 | 101.0239 | 104.1451 | 100.1986 | 104.9704 |
| 12 | 103.1397 | 1.207739 | 2001 | ASR | 102.3256 | 103.9537 | 102.124 | 104.1554 | 101.8884 | 104.3909 | 101.5913 | 104.688 | 100.7725 | 105.5068 |
| 13 | 103.2723 | 1.195349 | 2002 | ASR | 102.4667 | 104.078 | 102.267 | 104.2776 | 102.034 | 104.5107 | 101.7399 | 104.8048 | 100.9294 | 105.6152 |
| 14 | 103.312 | 1.185049 | 2003 | ASR | 102.5133 | 104.1108 | 102.3154 | 104.3087 | 102.0843 | 104.5397 | 101.7928 | 104.8313 | 100.9893 | 105.6347 |
| 15 | 103.0629 | 1.169037 | 2004 | ASR | 102.2749 | 103.8508 | 102.0797 | 104.046 | 101.8517 | 104.274 | 101.5642 | 104.5616 | 100.7716 | 105.3542 |
| 16 | 102.693 | 1.151175 | 2005 | ASR | 101.9171 | 103.4689 | 101.7249 | 103.6612 | 101.5004 | 103.8856 | 101.2172 | 104.1688 | 100.4367 | 104.9493 |
| 17 | 102.244 | 1.13194 | 2006 | ASR | 101.4811 | 103.0069 | 101.292 | 103.196 | 101.0713 | 103.4167 | 100.7929 | 103.6951 | 100.0254 | 104.4626 |
| 18 | 101.355 | 1.106978 | 2007 | ASR | 100.6089 | 102.1011 | 100.424 | 102.286 | 100.2082 | 102.5018 | 99.93585 | 102.7741 | 99.18532 | 103.5247 |
| 19 | 99.9912 | 1.076397 | 2008 | ASR | 99.26571 | 100.7167 | 99.08595 | 100.8964 | 98.87605 | 101.1063 | 98.61126 | 101.3711 | 97.88146 | 102.1009 |
| 20 | 98.65283 | 1.048941 | 2009 | ASR | 97.94585 | 99.35982 | 97.77068 | 99.53499 | 97.56613 | 99.73954 | 97.30809 | 99.99758 | 96.59691 | 100.7088 |
| 21 | 97.26962 | 1.023553 | 2010 | ASR | 96.57975 | 97.9595 | 96.40881 | 98.13043 | 96.20922 | 98.33002 | 95.95743 | 98.58182 | 95.26346 | 99.27579 |
| 22 | 95.89206 | 0.998476 | 2011 | ASR | 95.21909 | 96.56503 | 95.05234 | 96.73178 | 94.85764 | 96.92648 | 94.61201 | 97.17211 | 93.93505 | 97.84907 |
| 23 | 93.9512 | 0.967389 | 2012 | ASR | 93.29918 | 94.60322 | 93.13763 | 94.76478 | 92.94899 | 94.95342 | 92.71101 | 95.1914 | 92.05512 | 95.84729 |
| 24 | 91.41959 | 0.932839 | 2013 | ASR | 90.79085 | 92.04832 | 90.63507 | 92.2041 | 90.45317 | 92.38601 | 90.22369 | 92.61549 | 89.59122 | 93.24795 |
| 25 | 88.92474 | 0.900911 | 2014 | ASR | 88.31753 | 89.53196 | 88.16708 | 89.68241 | 87.9914 | 89.85809 | 87.76977 | 90.07971 | 87.15896 | 90.69053 |
| 26 | 87.04988 | 0.874384 | 2015 | ASR | 86.46055 | 87.63922 | 86.31452 | 87.78524 | 86.14402 | 87.95574 | 85.92892 | 88.17084 | 85.33609 | 88.76367 |
| 27 | 85.58661 | 0.850283 | 2016 | ASR | 85.01351 | 86.1597 | 84.87152 | 86.30169 | 84.70571 | 86.4675 | 84.49654 | 86.67667 | 83.92005 | 87.25316 |
| 28 | 84.64949 | 0.830195 | 2017 | ASR | 84.08994 | 85.20904 | 83.9513 | 85.34768 | 83.78941 | 85.50957 | 83.58518 | 85.7138 | 83.02231 | 86.27667 |
| 29 | 83.7735 | 0.811169 | 2018 | ASR | 83.22677 | 84.32023 | 83.09131 | 84.4557 | 82.93313 | 84.61387 | 82.73358 | 84.81342 | 82.18361 | 85.36339 |
| 30 | 82.94766 | 0.793112 | 2019 | ASR | 82.4131 | 83.48222 | 82.28065 | 83.61467 | 82.126 | 83.76933 | 81.93089 | 83.96443 | 81.39316 | 84.50216 |
| 31 | 82.49753 | 0.786715 | 2020 | ASR | 81.96728 | 83.02778 | 81.8359 | 83.15916 | 81.68249 | 83.31257 | 81.48896 | 83.5061 | 80.95557 | 84.03949 |
| 32 | 81.7752 | 0.846492 | 2021 | ASR | 81.20466 | 82.34574 | 81.0633 | 82.4871 | 80.89823 | 82.65217 | 80.69 | 82.8604 | 80.11608 | 83.43432 |
| 33 | 81.20748 | 1.66699 | 2022 | ASR | 80.08393 | 82.33103 | 79.80554 | 82.60942 | 79.48048 | 82.93448 | 79.0704 | 83.34456 | 77.94018 | 84.47478 |
| 34 | 80.6191 | 2.147624 | 2023 | ASR | 79.1716 | 82.0666 | 78.81295 | 82.42525 | 78.39416 | 82.84404 | 77.86584 | 83.37235 | 76.40976 | 84.82844 |
| 35 | 80.02944 | 2.550376 | 2024 | ASR | 78.31048 | 81.74839 | 77.88457 | 82.1743 | 77.38725 | 82.67163 | 76.75986 | 83.29902 | 75.0307 | 85.02817 |
| 36 | 79.44114 | 2.907596 | 2025 | ASR | 77.48142 | 81.40086 | 76.99585 | 81.88642 | 76.42887 | 82.45341 | 75.7136 | 83.16867 | 73.74225 | 85.14002 |
| 37 | 78.85303 | 3.234548 | 2026 | ASR | 76.67295 | 81.03312 | 76.13278 | 81.57329 | 75.50204 | 82.20402 | 74.70634 | 82.99972 | 72.51332 | 85.19275 |
| 38 | 78.26461 | 3.539843 | 2027 | ASR | 75.87875 | 80.65046 | 75.2876 | 81.24161 | 74.59733 | 81.93188 | 73.72653 | 82.80269 | 71.32652 | 85.2027 |
| 39 | 77.67607 | 3.827956 | 2028 | ASR | 75.09603 | 80.25611 | 74.45676 | 80.89538 | 73.71031 | 81.64183 | 72.76863 | 82.58351 | 70.17327 | 85.17886 |
| 40 | 77.0898 | 4.102408 | 2029 | ASR | 74.32477 | 79.85482 | 73.63967 | 80.53992 | 72.8397 | 81.33989 | 71.83051 | 82.34908 | 69.04908 | 85.13052 |
| 41 | 76.50791 | 4.366128 | 2030 | ASR | 73.56514 | 79.45068 | 72.83599 | 80.17982 | 71.9846 | 81.03121 | 70.91053 | 82.10528 | 67.95029 | 85.06552 |
| 42 | 75.92855 | 4.621415 | 2031 | ASR | 72.81372 | 79.04339 | 72.04194 | 79.81516 | 71.14077 | 80.71634 | 70.0039 | 81.85321 | 66.87058 | 84.98653 |
| 43 | 75.35089 | 4.870158 | 2032 | ASR | 72.06841 | 78.63338 | 71.25509 | 79.44669 | 70.30541 | 80.39638 | 69.10735 | 81.59443 | 65.80538 | 84.8964 |
| 44 | 74.77511 | 5.113238 | 2033 | ASR | 71.32879 | 78.22143 | 70.47488 | 79.07534 | 69.4778 | 80.07243 | 68.21994 | 81.33028 | 64.75316 | 84.79706 |
| 45 | 74.20308 | 5.35185 | 2034 | ASR | 70.59593 | 77.81022 | 69.70217 | 78.70398 | 68.65856 | 79.74759 | 67.342 | 81.06415 | 63.71345 | 84.6927 |
| 46 | 73.63622 | 5.587338 | 2035 | ASR | 69.87036 | 77.40209 | 68.93727 | 78.33517 | 67.84774 | 79.4247 | 66.47326 | 80.79919 | 62.68504 | 84.5874 |
| **Thailand** | | | | | | | | | | | | | | |
| 1 | 91.4917 | 0.554852 | 1990 | ASR | 91.11773 | 91.86567 | 91.02507 | 91.95833 | 90.91687 | 92.06653 | 90.78038 | 92.20302 | 90.40419 | 92.57921 |
| 2 | 92.35734 | 0.529114 | 1991 | ASR | 92.00071 | 92.71396 | 91.91235 | 92.80232 | 91.80917 | 92.9055 | 91.67901 | 93.03566 | 91.32027 | 93.3944 |
| 3 | 92.96686 | 0.519768 | 1992 | ASR | 92.61653 | 93.31718 | 92.52973 | 93.40398 | 92.42838 | 93.50534 | 92.30051 | 93.6332 | 91.94811 | 93.9856 |
| 4 | 93.55732 | 0.511697 | 1993 | ASR | 93.21243 | 93.9022 | 93.12698 | 93.98765 | 93.0272 | 94.08744 | 92.90132 | 94.21331 | 92.55439 | 94.56024 |
| 5 | 93.94791 | 0.503466 | 1994 | ASR | 93.60857 | 94.28724 | 93.52449 | 94.37132 | 93.42632 | 94.4695 | 93.30246 | 94.59335 | 92.96111 | 94.9347 |
| 6 | 93.98878 | 0.494782 | 1995 | ASR | 93.6553 | 94.32226 | 93.57267 | 94.40489 | 93.47619 | 94.50137 | 93.35447 | 94.62309 | 93.01901 | 94.95855 |
| 7 | 94.10179 | 0.486509 | 1996 | ASR | 93.77388 | 94.4297 | 93.69264 | 94.51094 | 93.59777 | 94.60581 | 93.47809 | 94.7255 | 93.14823 | 95.05535 |
| 8 | 92.99357 | 0.474898 | 1997 | ASR | 92.67348 | 93.31365 | 92.59418 | 93.39295 | 92.50157 | 93.48556 | 92.38475 | 93.60238 | 92.06277 | 93.92436 |
| 9 | 92.85704 | 0.465693 | 1998 | ASR | 92.54316 | 93.17092 | 92.46539 | 93.24869 | 92.37458 | 93.3395 | 92.26002 | 93.45406 | 91.94428 | 93.7698 |
| 10 | 93.03205 | 0.458022 | 1999 | ASR | 92.72335 | 93.34076 | 92.64686 | 93.41725 | 92.55754 | 93.50656 | 92.44487 | 93.61924 | 92.13433 | 93.92978 |
| 11 | 93.43181 | 0.450913 | 2000 | ASR | 93.1279 | 93.73573 | 93.05259 | 93.81103 | 92.96467 | 93.89896 | 92.85374 | 94.00988 | 92.54802 | 94.3156 |
| 12 | 94.16068 | 0.444148 | 2001 | ASR | 93.86132 | 94.46003 | 93.78715 | 94.5342 | 93.70054 | 94.62081 | 93.59128 | 94.73007 | 93.29015 | 95.03121 |
| 13 | 95.3027 | 0.43785 | 2002 | ASR | 95.00759 | 95.59781 | 94.93447 | 95.67094 | 94.84909 | 95.75632 | 94.74138 | 95.86403 | 94.44452 | 96.16089 |
| 14 | 95.725 | 0.429663 | 2003 | ASR | 95.43541 | 96.0146 | 95.36366 | 96.08635 | 95.27987 | 96.17014 | 95.17418 | 96.27583 | 94.88287 | 96.56714 |
| 15 | 96.28773 | 0.422212 | 2004 | ASR | 96.00316 | 96.57231 | 95.93265 | 96.64281 | 95.85032 | 96.72515 | 95.74646 | 96.82901 | 95.4602 | 97.11527 |
| 16 | 96.39299 | 0.41445 | 2005 | ASR | 96.11365 | 96.67233 | 96.04444 | 96.74154 | 95.96362 | 96.82236 | 95.86167 | 96.92431 | 95.58067 | 97.20531 |
| 17 | 93.22508 | 0.396419 | 2006 | ASR | 92.95789 | 93.49227 | 92.89169 | 93.55847 | 92.81439 | 93.63577 | 92.71687 | 93.73329 | 92.4481 | 94.00206 |
| 18 | 90.87516 | 0.381746 | 2007 | ASR | 90.61787 | 91.13246 | 90.55412 | 91.19621 | 90.47967 | 91.27065 | 90.38577 | 91.36456 | 90.12694 | 91.62339 |
| 19 | 88.66027 | 0.367845 | 2008 | ASR | 88.41234 | 88.9082 | 88.35091 | 88.96963 | 88.27918 | 89.04136 | 88.18869 | 89.13185 | 87.9393 | 89.38125 |
| 20 | 86.52931 | 0.354444 | 2009 | ASR | 86.29042 | 86.76821 | 86.23122 | 86.8274 | 86.16211 | 86.89651 | 86.07491 | 86.98371 | 85.8346 | 87.22402 |
| 21 | 85.14523 | 0.343376 | 2010 | ASR | 84.91379 | 85.37666 | 84.85645 | 85.43401 | 84.78949 | 85.50097 | 84.70502 | 85.58544 | 84.47221 | 85.81825 |
| 22 | 83.29839 | 0.331768 | 2011 | ASR | 83.07478 | 83.522 | 83.01937 | 83.5774 | 82.95468 | 83.6421 | 82.87306 | 83.72371 | 82.64812 | 83.94865 |
| 23 | 81.45052 | 0.320425 | 2012 | ASR | 81.23455 | 81.66649 | 81.18104 | 81.72 | 81.11856 | 81.78248 | 81.03973 | 81.8613 | 80.82249 | 82.07855 |
| 24 | 79.68776 | 0.309566 | 2013 | ASR | 79.47911 | 79.89641 | 79.42741 | 79.94811 | 79.36705 | 80.00847 | 79.2909 | 80.08462 | 79.08101 | 80.29451 |
| 25 | 78.59984 | 0.300584 | 2014 | ASR | 78.39725 | 78.80243 | 78.34705 | 78.85263 | 78.28844 | 78.91125 | 78.21449 | 78.98519 | 78.0107 | 79.18899 |
| 26 | 77.84878 | 0.292693 | 2015 | ASR | 77.6515 | 78.04605 | 77.60262 | 78.09493 | 77.54555 | 78.15201 | 77.47354 | 78.22401 | 77.2751 | 78.42246 |
| 27 | 77.42039 | 0.285677 | 2016 | ASR | 77.22784 | 77.61294 | 77.18014 | 77.66065 | 77.12443 | 77.71635 | 77.05415 | 77.78663 | 76.86046 | 77.98032 |
| 28 | 77.23228 | 0.279464 | 2017 | ASR | 77.04392 | 77.42064 | 76.99725 | 77.46731 | 76.94276 | 77.5218 | 76.87401 | 77.59055 | 76.68453 | 77.78003 |
| 29 | 77.83097 | 0.275214 | 2018 | ASR | 77.64548 | 78.01647 | 77.59952 | 78.06243 | 77.54585 | 78.11609 | 77.47815 | 78.1838 | 77.29155 | 78.37039 |
| 30 | 78.56776 | 0.271679 | 2019 | ASR | 78.38465 | 78.75087 | 78.33928 | 78.79624 | 78.2863 | 78.84922 | 78.21947 | 78.91605 | 78.03527 | 79.10025 |
| 31 | 78.5403 | 0.26736 | 2020 | ASR | 78.3601 | 78.7205 | 78.31545 | 78.76515 | 78.26332 | 78.81729 | 78.19755 | 78.88306 | 78.01628 | 79.06433 |
| 32 | 79.13029 | 0.267637 | 2021 | ASR | 78.9499 | 79.31068 | 78.90521 | 79.35537 | 78.85302 | 79.40756 | 78.78718 | 79.4734 | 78.60572 | 79.65486 |
| 33 | 79.46837 | 1.632298 | 2022 | ASR | 78.3682 | 80.56854 | 78.09561 | 80.84113 | 77.77731 | 81.15943 | 77.37576 | 81.56097 | 76.26906 | 82.66767 |
| 34 | 79.81302 | 2.199639 | 2023 | ASR | 78.33046 | 81.29558 | 77.96312 | 81.66292 | 77.53419 | 82.09185 | 76.99308 | 82.63296 | 75.50173 | 84.12431 |
| 35 | 80.18222 | 2.679915 | 2024 | ASR | 78.37596 | 81.98848 | 77.92841 | 82.43603 | 77.40583 | 82.95861 | 76.74657 | 83.61787 | 74.92959 | 85.43486 |
| 36 | 80.5784 | 3.115442 | 2025 | ASR | 78.47859 | 82.6782 | 77.95831 | 83.19848 | 77.3508 | 83.80599 | 76.5844 | 84.57239 | 74.47213 | 86.68466 |
| 37 | 80.99962 | 3.524947 | 2026 | ASR | 78.6238 | 83.37543 | 78.03514 | 83.9641 | 77.34777 | 84.65146 | 76.48064 | 85.5186 | 74.09072 | 87.90852 |
| 38 | 81.44181 | 3.918786 | 2027 | ASR | 78.80055 | 84.08308 | 78.14611 | 84.73751 | 77.38195 | 85.50168 | 76.41793 | 86.4657 | 73.76099 | 89.12263 |
| 39 | 81.89953 | 4.301796 | 2028 | ASR | 79.00012 | 84.79894 | 78.28172 | 85.51734 | 77.44287 | 86.35619 | 76.38463 | 87.41443 | 73.46801 | 90.33105 |
| 40 | 82.37297 | 4.677764 | 2029 | ASR | 79.22016 | 85.52578 | 78.43897 | 86.30697 | 77.52681 | 87.21913 | 76.37608 | 88.36986 | 73.20455 | 91.54139 |
| 41 | 82.86694 | 5.050168 | 2030 | ASR | 79.46313 | 86.27076 | 78.61975 | 87.11413 | 77.63497 | 88.09892 | 76.39263 | 89.34126 | 72.96861 | 92.76527 |
| 42 | 83.38556 | 5.422136 | 2031 | ASR | 79.73104 | 87.04008 | 78.82555 | 87.94558 | 77.76823 | 89.0029 | 76.43439 | 90.33674 | 72.75818 | 94.01295 |
| 43 | 83.9248 | 5.796017 | 2032 | ASR | 80.01829 | 87.83132 | 79.05035 | 88.79925 | 77.92013 | 89.92948 | 76.49431 | 91.3553 | 72.56461 | 95.28499 |
| 44 | 84.47178 | 6.171934 | 2033 | ASR | 80.31189 | 88.63166 | 79.28118 | 89.66237 | 78.07765 | 90.8659 | 76.55936 | 92.3842 | 72.37478 | 96.56877 |
| 45 | 85.02482 | 6.550943 | 2034 | ASR | 80.60948 | 89.44015 | 79.51547 | 90.53416 | 78.23804 | 91.81159 | 76.62651 | 93.42312 | 72.18497 | 97.86466 |
| 46 | 85.59079 | 6.935058 | 2035 | ASR | 80.91656 | 90.26502 | 79.7584 | 91.42317 | 78.40607 | 92.77551 | 76.70004 | 94.48153 | 71.99807 | 99.1835 |
| **Viet Nam** | | | | | | | | | | | | | | |
| 1 | 506.3945 | 1.921496 | 1990 | ASR | 505.0995 | 507.6896 | 504.7786 | 508.0105 | 504.4039 | 508.3852 | 503.9312 | 508.8579 | 502.6284 | 510.1607 |
| 2 | 506.4881 | 1.794684 | 1991 | ASR | 505.2785 | 507.6977 | 504.9787 | 507.9974 | 504.6288 | 508.3474 | 504.1873 | 508.7889 | 502.9705 | 510.0057 |
| 3 | 506.625 | 1.766605 | 1992 | ASR | 505.4343 | 507.8157 | 505.1393 | 508.1107 | 504.7948 | 508.4552 | 504.3602 | 508.8898 | 503.1625 | 510.0876 |
| 4 | 507.019 | 1.750618 | 1993 | ASR | 505.8391 | 508.199 | 505.5468 | 508.4913 | 505.2054 | 508.8327 | 504.7748 | 509.2633 | 503.5878 | 510.4503 |
| 5 | 508.0042 | 1.737207 | 1994 | ASR | 506.8333 | 509.1751 | 506.5432 | 509.4652 | 506.2045 | 509.804 | 505.7771 | 510.2313 | 504.5993 | 511.4091 |
| 6 | 508.871 | 1.723807 | 1995 | ASR | 507.7092 | 510.0329 | 507.4213 | 510.3208 | 507.0852 | 510.6569 | 506.6611 | 511.081 | 505.4924 | 512.2497 |
| 7 | 509.0688 | 1.708515 | 1996 | ASR | 507.9172 | 510.2203 | 507.6319 | 510.5056 | 507.2988 | 510.8388 | 506.8785 | 511.2591 | 505.7201 | 512.4175 |
| 8 | 509.4546 | 1.693431 | 1997 | ASR | 508.3132 | 510.596 | 508.0304 | 510.8788 | 507.7002 | 511.209 | 507.2836 | 511.6256 | 506.1355 | 512.7737 |
| 9 | 509.585 | 1.677401 | 1998 | ASR | 508.4544 | 510.7155 | 508.1743 | 510.9957 | 507.8472 | 511.3228 | 507.4345 | 511.7354 | 506.2973 | 512.8727 |
| 10 | 509.9464 | 1.661221 | 1999 | ASR | 508.8267 | 511.066 | 508.5493 | 511.3435 | 508.2254 | 511.6674 | 507.8167 | 512.0761 | 506.6904 | 513.2024 |
| 11 | 510.0291 | 1.64411 | 2000 | ASR | 508.921 | 511.1372 | 508.6464 | 511.4118 | 508.3258 | 511.7324 | 507.9213 | 512.1368 | 506.8066 | 513.2515 |
| 12 | 512.5055 | 1.629727 | 2001 | ASR | 511.4071 | 513.604 | 511.1349 | 513.8761 | 510.8171 | 514.1939 | 510.4162 | 514.5948 | 509.3113 | 515.6998 |
| 13 | 515.0003 | 1.61565 | 2002 | ASR | 513.9113 | 516.0892 | 513.6415 | 516.359 | 513.3265 | 516.6741 | 512.929 | 517.0715 | 511.8336 | 518.1669 |
| 14 | 516.6108 | 1.600026 | 2003 | ASR | 515.5324 | 517.6892 | 515.2652 | 517.9565 | 514.9532 | 518.2685 | 514.5596 | 518.6621 | 513.4748 | 519.7469 |
| 15 | 517.4557 | 1.582914 | 2004 | ASR | 516.3889 | 518.5226 | 516.1245 | 518.787 | 515.8158 | 519.0956 | 515.4264 | 519.485 | 514.3532 | 520.5582 |
| 16 | 517.8389 | 1.56544 | 2005 | ASR | 516.7838 | 518.894 | 516.5224 | 519.1555 | 516.2171 | 519.4607 | 515.832 | 519.8458 | 514.7707 | 520.9072 |
| 17 | 518.0108 | 1.546417 | 2006 | ASR | 516.9685 | 519.0531 | 516.7103 | 519.3113 | 516.4087 | 519.6129 | 516.0283 | 519.9933 | 514.9798 | 521.0418 |
| 18 | 518.2038 | 1.528118 | 2007 | ASR | 517.1738 | 519.2337 | 516.9186 | 519.4889 | 516.6206 | 519.7869 | 516.2447 | 520.1628 | 515.2086 | 521.1989 |
| 19 | 519.4266 | 1.512066 | 2008 | ASR | 518.4075 | 520.4457 | 518.155 | 520.6983 | 517.8601 | 520.9931 | 517.4881 | 521.3651 | 516.463 | 522.3903 |
| 20 | 522.5166 | 1.499027 | 2009 | ASR | 521.5062 | 523.5269 | 521.2559 | 523.7772 | 520.9636 | 524.0695 | 520.5948 | 524.4383 | 519.5785 | 525.4546 |
| 21 | 526.144 | 1.488608 | 2010 | ASR | 525.1407 | 527.1473 | 524.8921 | 527.3959 | 524.6018 | 527.6862 | 524.2356 | 528.0524 | 523.2263 | 529.0617 |
| 22 | 530.3457 | 1.476071 | 2011 | ASR | 529.3508 | 531.3405 | 529.1043 | 531.5871 | 528.8165 | 531.8749 | 528.4534 | 532.238 | 527.4526 | 533.2388 |
| 23 | 534.4821 | 1.464022 | 2012 | ASR | 533.4954 | 535.4689 | 533.2509 | 535.7134 | 532.9654 | 535.9988 | 532.6052 | 536.359 | 531.6126 | 537.3516 |
| 24 | 539.0095 | 1.45304 | 2013 | ASR | 538.0302 | 539.9888 | 537.7875 | 540.2315 | 537.5042 | 540.5148 | 537.1467 | 540.8723 | 536.1615 | 541.8575 |
| 25 | 544.4848 | 1.443828 | 2014 | ASR | 543.5117 | 545.458 | 543.2706 | 545.6991 | 542.989 | 545.9806 | 542.6339 | 546.3358 | 541.6549 | 547.3147 |
| 26 | 549.758 | 1.436833 | 2015 | ASR | 548.7896 | 550.7264 | 548.5496 | 550.9664 | 548.2694 | 551.2466 | 547.916 | 551.6 | 546.9418 | 552.5742 |
| 27 | 553.2795 | 1.423565 | 2016 | ASR | 552.32 | 554.239 | 552.0823 | 554.4767 | 551.8047 | 554.7543 | 551.4545 | 555.1045 | 550.4893 | 556.0697 |
| 28 | 556.6773 | 1.411378 | 2017 | ASR | 555.726 | 557.6286 | 555.4903 | 557.8643 | 555.2151 | 558.1395 | 554.8679 | 558.4867 | 553.911 | 559.4436 |
| 29 | 558.7653 | 1.397841 | 2018 | ASR | 557.8232 | 559.7075 | 557.5897 | 559.9409 | 557.3172 | 560.2135 | 556.9733 | 560.5573 | 556.0255 | 561.5051 |
| 30 | 559.6817 | 1.383245 | 2019 | ASR | 558.7494 | 560.614 | 558.5184 | 560.845 | 558.2486 | 561.1147 | 557.9084 | 561.455 | 556.9705 | 562.3928 |
| 31 | 560.6619 | 1.374803 | 2020 | ASR | 559.7353 | 561.5885 | 559.5057 | 561.8181 | 559.2376 | 562.0862 | 558.8994 | 562.4244 | 557.9673 | 563.3565 |
| 32 | 558.6391 | 1.394192 | 2021 | ASR | 557.6994 | 559.5788 | 557.4666 | 559.8116 | 557.1947 | 560.0835 | 556.8517 | 560.4264 | 555.9065 | 561.3717 |
| 33 | 557.0797 | 4.711428 | 2022 | ASR | 553.9042 | 560.2552 | 553.1174 | 561.042 | 552.1986 | 561.9607 | 551.0396 | 563.1197 | 547.8453 | 566.3141 |
| 34 | 557.0478 | 6.045342 | 2023 | ASR | 552.9732 | 561.1223 | 551.9636 | 562.1319 | 550.7848 | 563.3107 | 549.2976 | 564.7979 | 545.1989 | 568.8966 |
| 35 | 557.0238 | 7.188547 | 2024 | ASR | 552.1788 | 561.8689 | 550.9783 | 563.0694 | 549.5765 | 564.4712 | 547.8081 | 566.2396 | 542.9343 | 571.1134 |
| 36 | 556.9785 | 8.222201 | 2025 | ASR | 551.4367 | 562.5203 | 550.0636 | 563.8934 | 548.4603 | 565.4967 | 546.4376 | 567.5194 | 540.863 | 573.094 |
| 37 | 556.8647 | 9.187389 | 2026 | ASR | 550.6724 | 563.057 | 549.1381 | 564.5913 | 547.3466 | 566.3828 | 545.0865 | 568.6429 | 538.8574 | 574.872 |
| 38 | 556.7298 | 10.10997 | 2027 | ASR | 549.9157 | 563.5439 | 548.2273 | 565.2323 | 546.2558 | 567.2037 | 543.7688 | 569.6908 | 536.9142 | 576.5453 |
| 39 | 556.6204 | 10.99518 | 2028 | ASR | 549.2097 | 564.0312 | 547.3735 | 565.8674 | 545.2294 | 568.0114 | 542.5246 | 570.7163 | 535.0699 | 578.171 |
| 40 | 556.5477 | 11.84986 | 2029 | ASR | 548.5609 | 564.5345 | 546.582 | 566.5135 | 544.2713 | 568.8242 | 541.3562 | 571.7392 | 533.322 | 579.7735 |
| 41 | 556.49 | 12.68166 | 2030 | ASR | 547.9426 | 565.0374 | 545.8247 | 567.1553 | 543.3518 | 569.6282 | 540.2321 | 572.7479 | 531.6339 | 581.3461 |
| 42 | 556.4225 | 13.49867 | 2031 | ASR | 547.3244 | 565.5206 | 545.0701 | 567.7749 | 542.4379 | 570.4071 | 539.1172 | 573.7278 | 529.9651 | 582.8799 |
| 43 | 556.4005 | 14.31027 | 2032 | ASR | 546.7554 | 566.0456 | 544.3655 | 568.4354 | 541.575 | 571.2259 | 538.0547 | 574.7463 | 528.3523 | 584.4486 |
| 44 | 556.4307 | 15.11397 | 2033 | ASR | 546.2439 | 566.6175 | 543.7198 | 569.1415 | 540.7726 | 572.0887 | 537.0546 | 575.8068 | 526.8073 | 586.054 |
| 45 | 556.5134 | 15.91086 | 2034 | ASR | 545.7894 | 567.2373 | 543.1323 | 569.8944 | 540.0297 | 572.997 | 536.1156 | 576.9111 | 525.3281 | 587.6987 |
| 46 | 556.6353 | 16.70452 | 2035 | ASR | 545.3765 | 567.8942 | 542.5868 | 570.6838 | 539.3294 | 573.9412 | 535.2201 | 578.0505 | 523.8945 | 589.3762 |

**Table S3:** **Joinpoint regression analysis results of ASMR from falls attributed to LBMD from 1990 to 2021**

| **Location** | **Start year** | **End year** | **APC (95% CI) (%)** | ***P* value** |
| --- | --- | --- | --- | --- |
| **ASMR** | | | | |
| Global | 1990 | 2000 | -0.51(-0.80, -0.23) | < 0.001 |
|  | 2000 | 2003 | 2.01(0.17, 3.88) | < 0.001 |
|  | 2003 | 2019 | 0.18(0.04, 0.32) | < 0.001 |
|  | 2019 | 2021 | -1.43(-3.21, 0.38) | >0.05 |
| China | 1990 | 1998 | -0.88(-1.16, -0.59) | < 0.001 |
|  | 1998 | 2004 | 6.72(6.17, 7.28) | < 0.001 |
|  | 2004 | 2012 | -2.66(-3.06, -2.25) | < 0.001 |
|  | 2012 | 2021 | -0.97(-1.21, -0.73) | < 0.001 |
| Brunei Darussalam | 1990 | 1997 | -1.59(-2.22, -0.95) | < 0.001 |
|  | 1997 | 2007 | -0.28(-0.74, 0.18) | >0.05 |
|  | 2007 | 2017 | 2.53(2.06, 3.00) | < 0.001 |
|  | 2017 | 2021 | -8.79(-10.48, -7.08) | < 0.001 |
| Cambodia | 1990 | 1996 | 0.32(0.27, 0.38) | < 0.001 |
|  | 1996 | 2005 | -0.30(-0.34, -0.27) | < 0.001 |
|  | 2005 | 2017 | 1.17(1.14, 1.20) | < 0.001 |
|  | 2017 | 2021 | 0.36(0.27, 0.46) | < 0.001 |
| Indonesia | 1990 | 2006 | 0.85(0.75, 0.95) | < 0.001 |
|  | 2006 | 2010 | -4.53(-5.37, -3.68) | < 0.001 |
|  | 2010 | 2014 | 1.80(0.91, 2.71) | < 0.001 |
|  | 2014 | 2021 | -0.40(-0.77, -0.03) | < 0.001 |
| Laos | 1990 | 2001 | 0.22(0.15, 0.28) | < 0.001 |
|  | 2001 | 2004 | 0.46(0.16, 0.75) | < 0.001 |
|  | 2004 | 2017 | -0.13(-0.18, -0.08) | < 0.001 |
|  | 2017 | 2021 | -0.36(-0.65, -0.07) | < 0.001 |
| Malaysia | 1990 | 1999 | 0.83(-0.03, 1.71) | >0.05 |
|  | 1999 | 2010 | 0.07(-0.48, 0.63) | >0.05 |
|  | 2010 | 2015 | -0.68(-2.76, 1.43) | >0.05 |
|  | 2015 | 2021 | -2.40(-3.94, -0.83) | < 0.001 |
| Myanmar | 1990 | 1998 | 0.28(0.20, 0.36) | < 0.001 |
|  | 1998 | 2003 | -0.28(-0.41, -0.16) | < 0.001 |
|  | 2003 | 2013 | -1.41(-1.48, -1.34) | < 0.001 |
|  | 2013 | 2021 | -0.11(-0.18, -0.04) | < 0.001 |
| Philippines | 1990 | 1992 | -9.14(-11.48, -6.73) | < 0.001 |
|  | 1992 | 2004 | 0.09(-0.06, 0.25) | >0.05 |
|  | 2004 | 2009 | 1.41(0.96, 1.86) | < 0.001 |
|  | 2009 | 2021 | 0.36(0.21, 0.52) | < 0.001 |
| Singapore | 1990 | 1992 | 6.08(-1.03, 13.70) | >0.05 |
|  | 1992 | 2001 | 0.65(0.11, 1.20) | < 0.001 |
|  | 2001 | 2016 | -2.41(-2.73, -2.10) | < 0.001 |
|  | 2016 | 2021 | -1.76(-2.90, -0.60) | < 0.001 |
| Thailand | 1990 | 2005 | -0.46(-0.61, -0.31) | < 0.001 |
|  | 2005 | 2012 | -6.07(-6.52, -5.61) | < 0.001 |
|  | 2012 | 2016 | -2.27(-3.07, -1.48) | < 0.001 |
|  | 2016 | 2021 | 1.26(0.44, 2.09) | < 0.001 |
| Viet Nam | 1990 | 2005 | 0.36(0.32, 0.40) | < 0.001 |
|  | 2005 | 2008 | -0.54(-0.82, -0.25) | < 0.001 |
|  | 2008 | 2017 | 0.19(0.10, 0.27) | < 0.001 |
|  | 2017 | 2021 | -0.62(-0.91, -0.33) | < 0.001 |

The data in parentheses for APC represents the 95% CI. Abbreviations: LBMD, Low bone mineral density, ASDR: Age-Standardized Death Rate.

**Table S4: Joinpoint regression analysis results of ASDR from falls attributed to LBMD from 1990 to 2021**

| **Location** | **Start year** | **End year** | **APC (95% CI) (%)** | ***P* value** |
| --- | --- | --- | --- | --- |
| **Age-standardized DALYs rate** | | | | |
| Global | 1990 | 1999 | -0.13(-0.19, -0.07) | < 0.001 |
|  | 1999 | 2002 | 0.57(-0.14, 1.28) | >0.05 |
|  | 2002 | 2011 | -0.40(-0.47, -0.34) | < 0.001 |
|  | 2011 | 2021 | -0.01(-0.05, 0.04) | >0.05 |
| China | 1990 | 1996 | -0.94(-1.32, -0.55) | < 0.001 |
|  | 1996 | 2002 | 1.44(1.04, 1.83) | < 0.001 |
|  | 2002 | 2010 | -1.81(-2.01, -1.61) | < 0.001 |
|  | 2010 | 2021 | 1.80(1.64, 1.96) | < 0.001 |
| Brunei Darussalam | 1990 | 1997 | -0.41(-0.60, -0.21) | < 0.001 |
|  | 1997 | 2005 | 0.09(-0.05, 0.22) | >0.05 |
|  | 2005 | 2017 | 0.25(0.16, 0.33) | < 0.001 |
|  | 2017 | 2021 | -2.31(-2.76, -1.85) | < 0.001 |
| Cambodia | 1990 | 1996 | 0.40(0.33, 0.46) | < 0.001 |
|  | 1996 | 2005 | 0.07(0.04, 0.10) | < 0.001 |
|  | 2005 | 2017 | 1.22(1.19, 1.24) | < 0.001 |
|  | 2017 | 2021 | 0.54(0.45, 0.63) | < 0.001 |
| Indonesia | 1990 | 2000 | 0.62(0.51, 0.73) | < 0.001 |
|  | 2000 | 2007 | -0.72(-0.91, -0.54) | < 0.001 |
|  | 2007 | 2010 | -3.49(-3.91, -3.06) | < 0.001 |
|  | 2010 | 2021 | -0.17(-0.26, -0.07) | < 0.001 |
| Laos | 1990 | 2000 | 0.14(0.11, 0.16) | < 0.001 |
|  | 2000 | 2008 | 0.36(0.33, 0.40) | < 0.001 |
|  | 2008 | 2012 | -0.14(-0.29, 0.02) | >0.05 |
|  | 2012 | 2021 | 0.04(0.02, 0.07) | < 0.001 |
| Malaysia | 1990 | 1995 | -0.47(-1.30, 0.36) | >0.05 |
|  | 1995 | 1998 | 1.03(-0.15, 2.23) | >0.05 |
|  | 1998 | 2016 | -0.24(-0.36, -0.12) | < 0.001 |
|  | 2016 | 2021 | -0.99(-1.81, -0.17) | < 0.001 |
| Myanmar | 1990 | 1998 | 0.24(0.19, 0.30) | < 0.001 |
|  | 1998 | 2003 | -0.09(-0.28, 0.10) | >0.05 |
|  | 2003 | 2013 | -0.63(-0.67, -0.59) | < 0.001 |
|  | 2013 | 2021 | 0.39(0.32, 0.46) | < 0.001 |
| Philippines | 1990 | 1992 | -2.81(-3.94, -1.67) | < 0.001 |
|  | 1992 | 2004 | -0.39(-0.45, -0.33) | < 0.001 |
|  | 2004 | 2009 | 0.86(0.49, 1.23) | < 0.001 |
|  | 2009 | 2021 | 0.25(0.19, 0.31) | < 0.001 |
| Singapore | 1990 | 2001 | 1.11(0.95, 1.27) | < 0.001 |
|  | 2001 | 2009 | -0.46(-0.72, -0.21) | < 0.001 |
|  | 2009 | 2016 | -2.22(-2.47, -1.97) | < 0.001 |
|  | 2016 | 2021 | -0.81(-1.33, -0.29) | < 0.001 |
| Thailand | 1990 | 2005 | 0.32(0.23, 0.40) | < 0.001 |
|  | 2005 | 2009 | -2.63(-3.06, -2.19) | < 0.001 |
|  | 2009 | 2015 | -1.81(-2.14, -1.49) | < 0.001 |
|  | 2015 | 2021 | 0.47(0.13, 0.81) | < 0.001 |
| Viet Nam | 1990 | 1992 | -0.14(-0.73, 0.45) | >0.05 |
|  | 1992 | 2009 | 0.17(0.15, 0.19) | < 0.001 |
|  | 2009 | 2017 | 0.84(0.79, 0.90) | < 0.001 |
|  | 2017 | 2021 | 0.01(-0.18, 0.20) | >0.05 |

The data in parentheses for APC represents the 95% CI. Abbreviations: LBMD, Low bone mineral density.

##
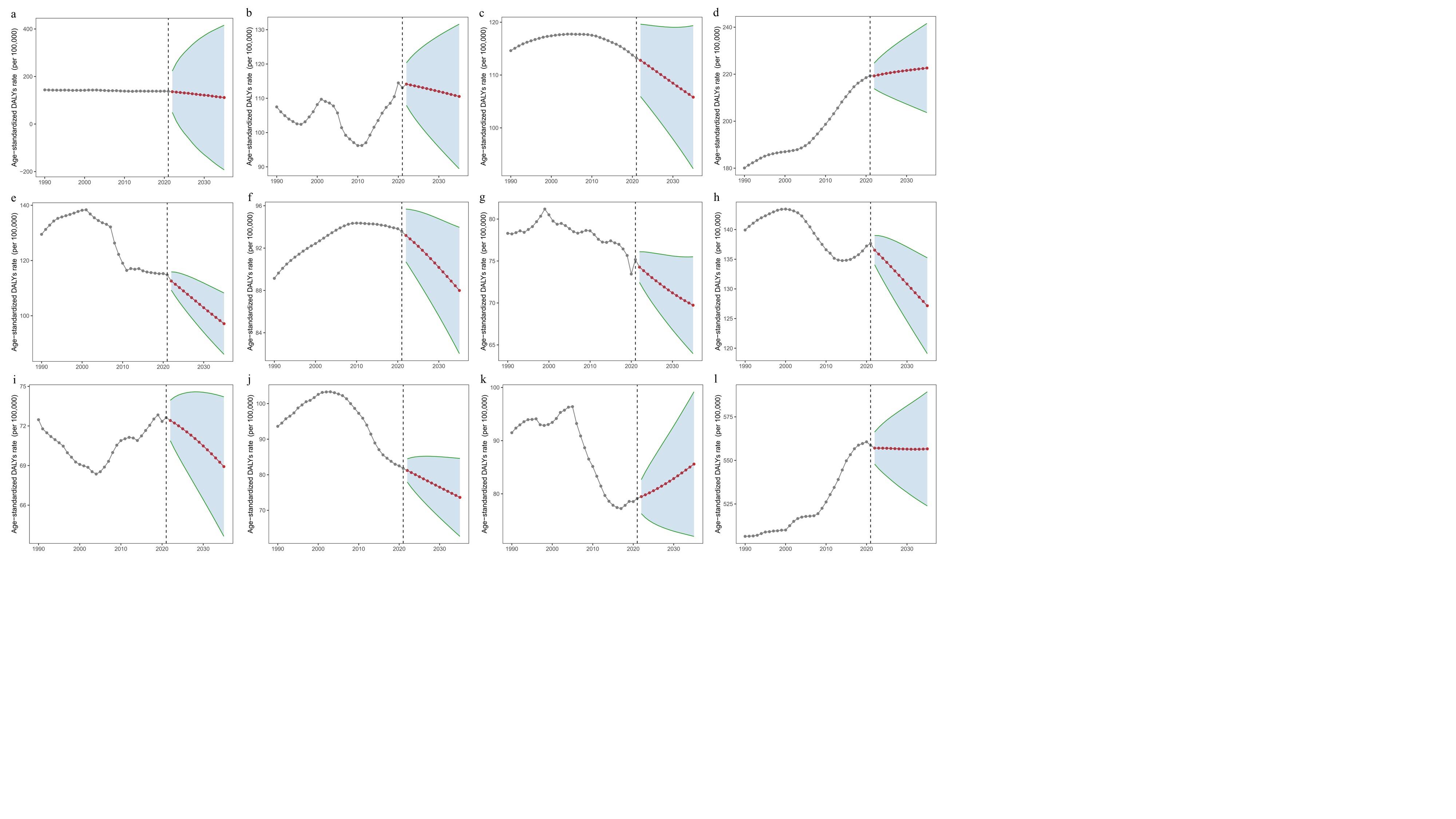


**Figure. S1. The predicted ASDR globally from 1990 to 2035, based on the BAPC model.** a: Global, b: China c: Brunei Darussalam, d: Cambodia, e: Indonesia, f: Lao People's Democratic Republic, g: Malaysia, h: Myanmar, i: Philippines, j: Singapore, k: Thailand, l: Viet Nam, Abbreviations: ASDR: Age-Standardized DALY rates


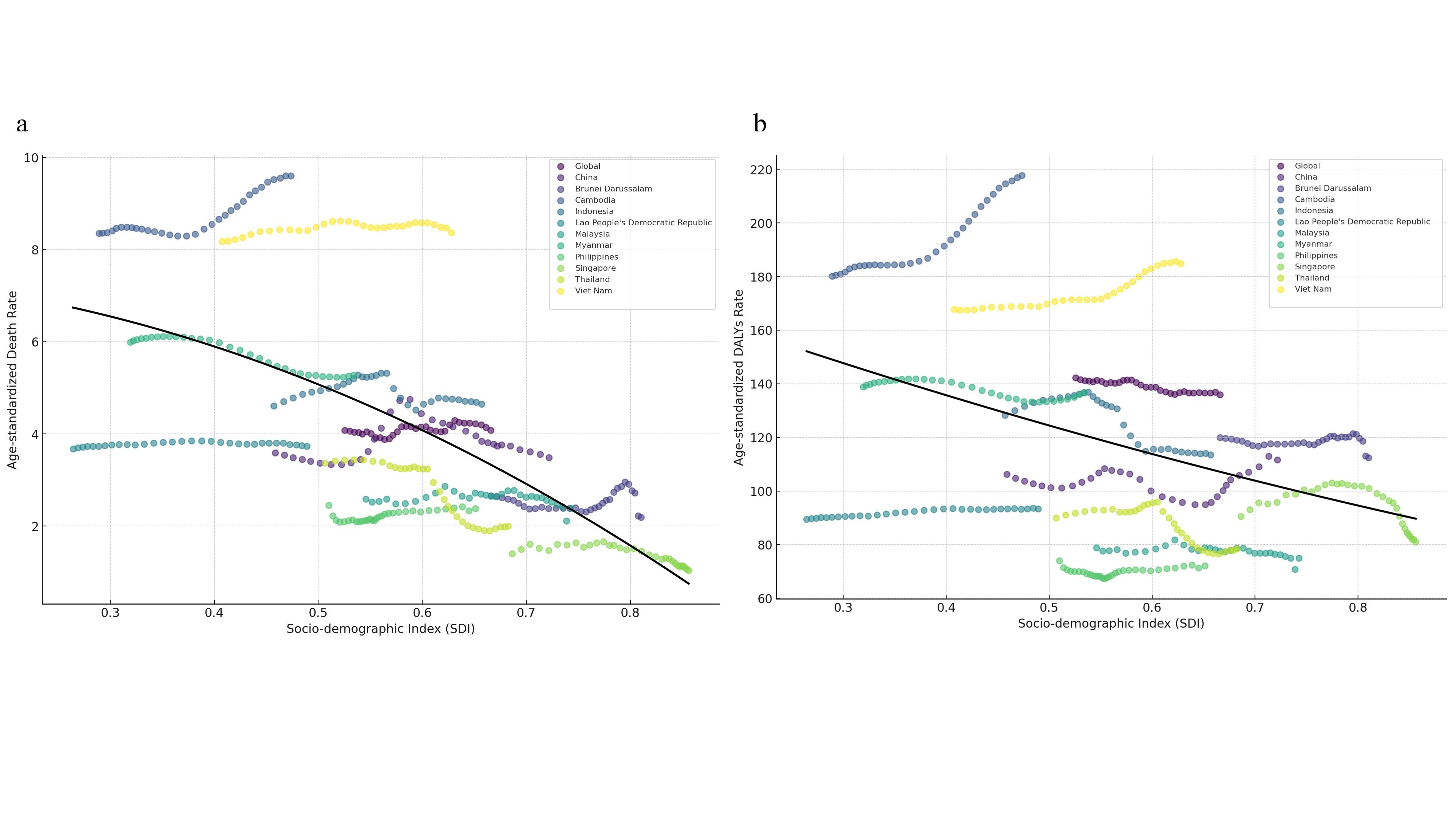


**Figure S2: Association Between SDI, ASMR, and ASDR for Selected Countries, 1990-2021** (a). ASMR (b) ASDR, Abbreviations: ASMR: Age-Standardized Mortality Rate, ASDR: Age-Standardized DALY rates, DALYs, disability-adjusted life years. SDI: Socio-demographic Index.


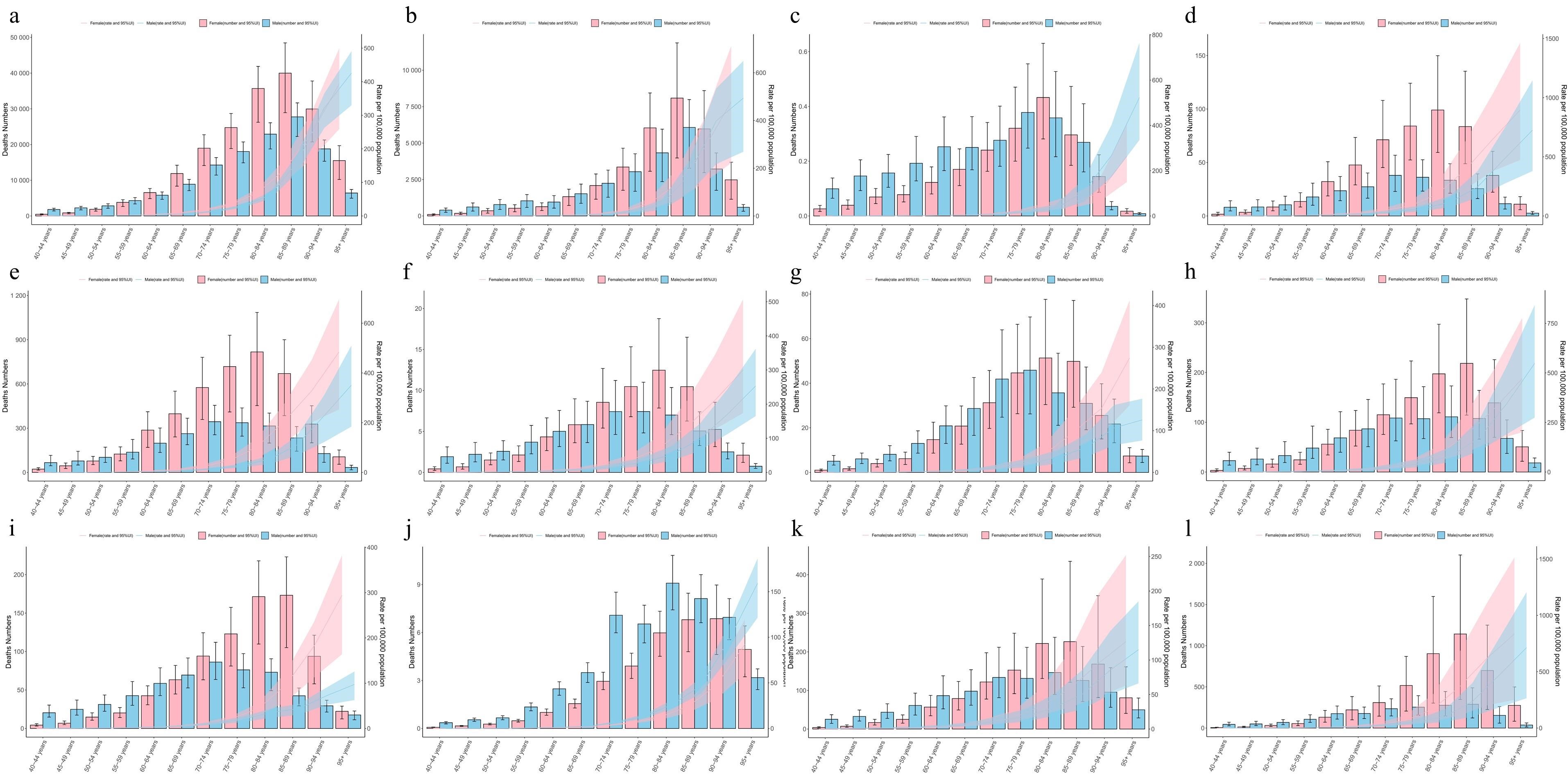


**Figure S3: Age- and sex-specific analysis of ASMR in the world, China, and the ASEAN countries (2021)** a: Global, b: China c: Brunei Darussalam, d: Cambodia, e: Indonesia, f: Lao People's Democratic Republic, g: Malaysia, h: Myanmar, i: Philippines, j: Singapore, k: Thailand, l: Viet Nam, Abbreviations: ASMR: Age-Standardized Mortality Rate.


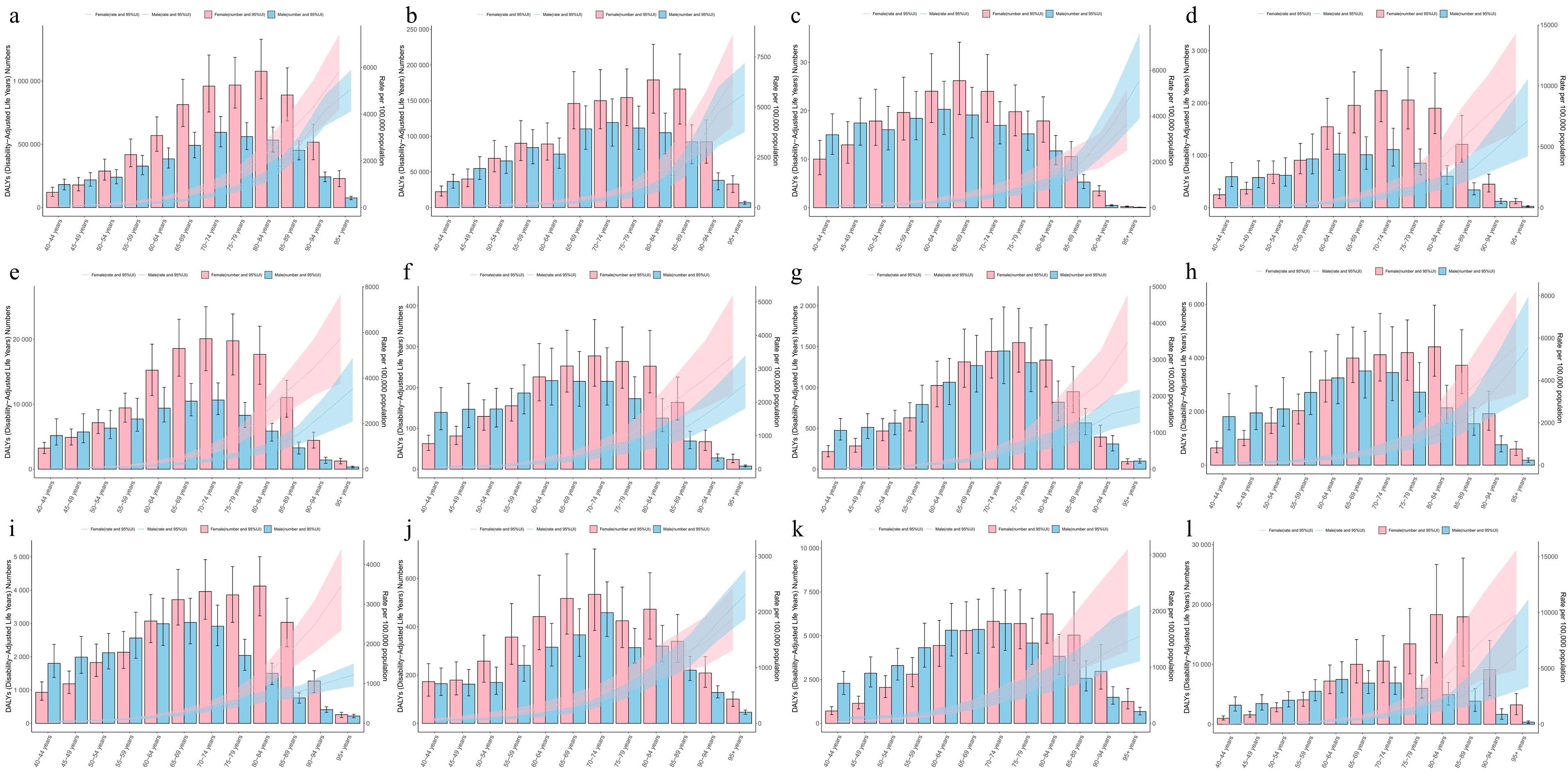


**Figure S4: Age- and sex-specific analysis of ASDR in the world, China, and the ASEAN countries (2021)** a: Global, b: China c: Brunei Darussalam, d: Cambodia, e: Indonesia, f: Lao People's Democratic Republic, g: Malaysia, h: Myanmar, i: Philippines, j: Singapore, k: Thailand, l: Viet Nam, Abbreviations: ASDR: Age-Standardized DALY rates.

## Appendix 1 Overview of data processing workflow and methodological enhancements in GBD 2021

In GBD 2021, the data processing workflow is a crucial step to ensure the accuracy and consistency of global disease burden estimates. This process includes systematic adjustments to epidemiological data to correct for biases arising from differences in data sources, definitions, and measurement methods. These adjustments are implemented using complex statistical models such as MR-BRT and DisMod-MR 2.1, ensuring internal consistency in estimates of incidence, prevalence, years lived with disability (YLDs), years of life lost (YLLs), and disability-adjusted life years (DALYs) across different regions, ages, genders, and years. This process aims to minimize heterogeneity in the study results through standardization and correction steps.

**1. Identification and extraction of data**

In GBD 2021, estimating the nonfatal disease burden begins with the systematic identification and extraction of diverse data sources. This process includes not only systematic reviews of 77 diseases and risk factors but also the extraction of key data from surveys, disease registries, case reports, and hospitalization data. Particularly crucial are the survey data extracted from the Global Health Data Exchange (GHDx), such as the Demographic and Health Surveys (DHS), which capture a wide range of health outcomes across different regions and populations. The estimation of DALYs in GBD 2021 is based on 100,983 data sources, of which 19,189 were newly added in 2021. These sources include 75,459 data sources for nonfatal causes, covering incidence, prevalence, and other epidemiological indicators. Data sources for fatality estimation include death registrations, verbal autopsies, registries, surveys, and police or surveillance data, covering all countries and regions globally. GBD research efforts incorporated systematic reviews, proactive data collection, and contributions from a global network of collaborators to include as much data as possible from around the world.

**2. Data processing and heterogeneity control**

In GBD 2021, adjustments were made to epidemiological data known to have biases, such as those using alternative case definitions or measurement methods. These adjustments were made using correction factors estimated by MR-BRT (Meta-regression—Bayesian, regularized, trimmed), a collection of statistical models including linear and nonlinear mixed-effects models. The input data included paired estimates of two case definitions or measurement methods for the same age, sex, region, and year. MR-BRT also controlled for heterogeneity through network meta-regression and performed sex-splitting for inputs not reported by sex, and age-sex splitting for data not reported by either. These processes ensured data standardization and consistency, reducing heterogeneity issues caused by varying data sources and definitions. Additionally, input data spanning more than 25 years were disaggregated into finer age-specific estimates using alternative age patterns estimated from other available data sources.

**3. Epidemiological estimation and YLD calculation**

The prevalence and incidence estimates for most diseases and injuries were derived using DisMod-MR 2.1 (Disease Modeling Meta-Regression Tool, version 2.1). This Bayesian disease modeling tool generates internally consistent prevalence, incidence, remission, and mortality estimates stratified by sex, region, year, and age group. For areas lacking original epidemiological data, DisMod-MR 2.1 utilizes data from higher hierarchical levels as prior information to estimate parameters for lower levels. For certain causes, the Space-Time Gaussian Process Regression (ST-GPR) model was used as an alternative estimation method. For nonfatal causes, prevalence and incidence were further divided into specific sequelae estimates based on their severity. Sequela categories for nonfatal causes can range from asymptomatic to severe, depending on the disease. For most nonfatal causes, the proportion of cases in each sequela category was calculated using Medical Expenditure Panel Survey (MEPS) analysis. Crude YLD rates were estimated by multiplying the sequela-specific prevalence by the corresponding disability weights.

**4. Comorbidity adjustment**

GBD 2021 adjusted YLDs for comorbidities to account for the coexistence of nonfatal causes in the population, allowing YLDs to be additive within the GBD 2021 cause hierarchy. The coexistence of comorbidities was estimated through simulations involving 20,000 hypothetical individuals for each age, sex, region, and year. Each simulated individual was assigned the probability of having each sequela based on its prevalence. Subsequently, a cumulative disability weight was assigned to each individual by multiplying the disability weights of all assigned sequelae, with appropriate adjustments made to the sequela-specific disability weights.

**5. YLLs and DALYs estimation process**

In GBD 2021, YLLs were calculated by multiplying the estimated number of deaths by the standard life expectancy at the age of death, stratified by age, sex, region, and year. To ensure accurate attribution of causes of death, GBD 2021 employed the principles of the 11th edition of the International Classification of Diseases (ICD-11), assigning each death to the underlying cause that initiated the chain of events leading to death. For deaths recorded with non-specific, unreliable, or intermediate cause codes, reallocation algorithms were applied to reassign these "garbage codes" to the most probable causes of death. These algorithms were derived from published studies, expert consultations, or regression-based adjustments using data from sources reporting multiple causes of death. The cause of death for most diseases and injuries is estimated using the Cause of Death Ensemble model (CODEm). CODEm employs a set of statistical models, systematically testing the predictive validity of different covariate combinations, then combining the results to estimate the number of deaths for specific causes by location, age, sex, and year. For a small number of causes with sparse data or significant changes in reporting practices, GBD 2021 adopted customized modeling strategies, including the use of prevalence, incidence, case fatality data, or data related to sub-causes to infer causes of death. Through this process, GBD 2021 achieved progress in controlling data heterogeneity and reducing uncertainty. To estimate DALYs in GBD 2021, specific cause mortality rates and YLDs were first estimated. DALYs for each year were then calculated by adding YLLs to YLDs. The uncertainty of YLLs was assumed to be independent of the uncertainty of YLDs. By summing the first set of YLLs and YLDs across 500 simulations, and repeating the process for subsequent simulations, the 95% uncertainty interval for DALYs was ultimately calculated. The estimation of DALYs covered every cause, location, age group, sex, and year, providing a comprehensive assessment of the global health burden.

**6. Uncertainty and heterogeneity analysis**

GBD 2021 included extensive uncertainty analysis to ensure the robustness of the estimates. By generating multiple simulations (usually 500) for each population group, accounting for uncertainties in prevalence and DWs, and reporting 95% uncertainty intervals, GBD 2021 provided reliable ranges for disease burden estimates. Additionally, GBD 2021 addressed heterogeneity by adjusting for known biases in the data, ensuring that changes in estimates reflected actual differences in disease burden rather than inconsistencies in data sources. This rigorous approach to uncertainty and heterogeneity analysis is crucial for making health policy decisions based on GBD results.

**7. Data release and application**

GBD 2021's findings were disseminated through various channels, including scientific publications, interactive visualization tools, and the GHDx. These tools enable researchers, policymakers, and the public to explore and compare health data across different regions and time periods. The ultimate goal of GBD 2021 is to provide a comprehensive framework for understanding global and local health trends, thereby supporting evidence-based decision-making and effective allocation of public health resources.

**8. Frontier analysis**

Estimate the frontier through Bootstrap resampling and determine whether each point exceeds efficiency. The purpose of this analysis is to calculate a representative efficiency frontier value for each observation point and to assess which points lie beyond or on that frontier line.

### 1. Calculate the frontier points:

For each point *i*, compare its value with all previous points and use the minimum value among them as the frontier value.

$$\mathrm{frontier}_{i}=\min_{j=1,\ldots,i-1} v_{i}$$

frontier_i_: Indicates the frontier point;

*j*: Indicates j frontier points;

*v*: Indicates the value of the frontier point.

### 2. Fitting efficiency along the front

To plot efficiency frontiers using the relationship between the Socio-Demographic Index (SDI) and the ASR of disease burden, we can follow these steps using the local polynomial regression (Loess) method:

$$y=f\left( x \right)+\epsilon$$

$y$: Indicates the ASR;

$x$: Indicates the SDI;

$f(x)$: Indicates the frontier function.

The frontier function describes the optimal frontier, which represents the maximum output or minimum input achievable given the inputs, and measures the degree of efficiency and deviation. The frontier is represented by a black curve.

### 3. Super efficiency point evaluation:

If the *v_i_* of a point is less than or equal to the frontier value before it, it is considered a frontier point; otherwise, it is an over-efficient point.

$${super}_{i}=\left\{ \begin{aligned} 1, if v_{i}\geq\mathrm{frontier}_{i-1} \\ 0, \mathrm{otherwise} \end{aligned} \right.$$

### 4. Averaging Bootstrap Results:

After multiple Bootstrap samplings, the average of the frontier values for each point is taken to obtain the final frontier estimate.

$$\overline{\mathrm{frontier}}=\frac{1}{\mathrm{num}}\sum_{k=1}^{\mathrm{num}} \mathrm{frontier}_{i}^{\left( k \right)}$$

$\overline{\mathrm{frontier}}$: Indicates the average value of the frontier points;

Num: Indicates the number of multiple sampling instances.

### 5. Explanation of Frontier analysis

By using multiple resampling (Bootstrap) and DEA frontier analysis, calculate the frontier values for each point and identify which points are super-efficient points. In each resampling, the data is reordered, evaluated, and the frontier is updated by excluding certain points, resulting in robust estimates.

**9. New additions in GBD 2021**

In GBD 2021, estimates for all age groups under 5 years were further subdivided into 0-6 days, 7-27 days, 1-5 months, 6-11 months, 12-23 months, and 2-4 years. Additionally, GBD 2021 for the first time reported five additional Level 3 causes and five additional Level 4 causes. The modeling and reporting of new causes considered data availability, policy concerns, research priorities, and methodological improvements. COVID-19-related causes were included for the first time, encompassing long COVID burden and other pandemic-related outcomes. To better capture the impact of the pandemic, prevalence estimates for severe depression and anxiety were adjusted accordingly.

Through this comprehensive process, GBD 2021 ensured the scientific rigor and global comparability of disease burden estimates, providing a reliable foundation for global health policy and planning by controlling heterogeneity and uncertainty. The rigorous methods and advanced modeling techniques employed by GBD 2021 reflect the high standards and precision necessary to understand and address global health challenges.
